# Morphological and functional characteristics of mitral annular calcification and their relationship to stroke

**DOI:** 10.1371/journal.pone.0227753

**Published:** 2020-01-13

**Authors:** Darae Kim, Chi Young Shim, Geu-Ru Hong, Hyeonju Jeong, Jong-Won Ha

**Affiliations:** 1 Division of Cardiology, Department of Medicine, Heart Vascular Stroke Institute, Samsung Medical Center, Sungkyunkwan University School of Medicine, Seoul, Republic of Korea; 2 Division of Cardiology, Severance Cardiovascular Hospital, Yonsei University College of Medicine, Seoul, Republic of Korea; University of Messina, ITALY

## Abstract

**Background:**

Mitral annular calcification (MAC) is associated with risk of stroke. This study aimed to define the morphological and functional characteristics of MAC that are related to stroke.

**Methods:**

A total of 460 subjects with MAC from transthoracic echocardiography in a single center from 2012 to 2016 was retrospectively reviewed. Subjects were classified into two groups according to history of stroke [Group 1 (n = 366): without stroke; Group 2 (n = 94): with stroke]. Morphological and functional features of MAC on echocardiogram were scored from 0 to 3 according to MAC mobility, presence of echodense mass with central echolucencies in the periannular region suggesting caseous necrosis, and functional stenosis.

**Results:**

Significantly more patients in group 2 were men and had history of diabetes mellitus, dyslipidemia, atrial fibrillation, or infective endocarditis. Although MAC thickness and extent did not differ between the two groups, group 2 showed a considerably higher MAC score than group 1 (0.50 ± 0.77 vs. 0.23 ±0.52 p<0.001) as a result of the higher prevalence of each component in group 2 [mobility (22 vs. 11%, p = 0.003), echodense mass with central areas of echolucencies suggesting caseous necrosis (23 vs. 7%, p<0.001), and functional mitral stenosis (12 vs. 7%, p = 0.042)]. On logistic regression analysis, MAC score was independently associated with stroke and showed significant incremental value to demographic factors and comorbidities in association with stroke in a consecutive manner.

**Conclusions:**

In conclusion, morphological and functional characteristics of MAC had incremental value in association with stroke over traditional risk factors. MAC score consisting of MAC mobility, typical echodense mass with central echolucencies suggesting caseous necrosis, and functional mitral stenosis was independently associated with stroke. MAC with high-risk features may act as a source of stroke or more potent composite surrogate markers for stroke-related risk factors.

## Introduction

Mitral annular calcification (MAC) is a chronic degenerative process of the fibrous skeleton of the mitral ring [1]. MAC was considered a benign, incidental finding in elderly women and those with chronic kidney disease [2,3]. However, growing evidence shows that MAC is associated with cardiovascular morbidity and mortality [4–9]. Previous cohort studies suggested that MAC was associated with stroke [10–12]. MAC was independently associated with aortic atheroma and coronary artery disease [13,14]. The MESA study showed a strong correlation between atrial fibrillation and MAC and increased risk of atrial fibrillation in patients with MAC progression [15,16]. In addition, MAC is suggested as a possible nidus for infective endocarditis and associated vegetation that would increase risk of stroke [17]. Some studies, however, suggest an association between MAC and stroke as resulting from shared risk factors [18,19].

In this study, we hypothesized that MAC associated with stroke would have distinctive characteristics compared to quiescent, benign MAC. Therefore, we aimed to explore if structural and functional characteristics of MAC are associated with stroke and suggest high-risk features as scores to facilitate risk stratification for stroke in MAC patients.

## Materials and methods

### Study population

The study population comprised 460 patients with incidentally diagnosed MAC from transthoracic echocardiography from 2012 to 2016 from Severance Cardiovascular Hospital, Seoul, Korea. To define characteristics of MAC associated with stroke, patients were classified into two groups according to previous history of stroke [Group 1 (n = 366): without stroke; Group 2 (n = 94): with stroke]. Pertinent clinical data were obtained retrospectively from electronic medical records. Patients with severe aortic stenosis or rheumatic valve disease were excluded from this analysis. Clinical medical history was based on medical records including history of stroke, infective endocarditis, hypertension, diabetes mellitus, atrial fibrillation, dyslipidemia, hypertrophic cardiomyopathy, end stage renal disease, coronary artery disease, and congestive heart failure. Stroke was defined as rapidly developed clinical signs of focal neurological deficit lasting 24 hours or leading to death with no evidence of other than vascular origin [20]. History of infective endocarditis was defined as previous images of vegetation and medical records that satisfied Duke Criteria. Blood pressure and plasma laboratory results were acquired on the same day as echocardiography. Estimated glomerular filtration rate was calculated using the Modification of Diet in Renal Disease Study equation [21]. Patients were classified into two groups according to previous history of stroke to compare morphological and functional characteristics of MAC in patients with or without stroke. All strokes occurred within 3 years prior to echocardiography. The Institutional Review Board of Severance Hospital approved the research.

### Echocardiography

**Fig 1:**
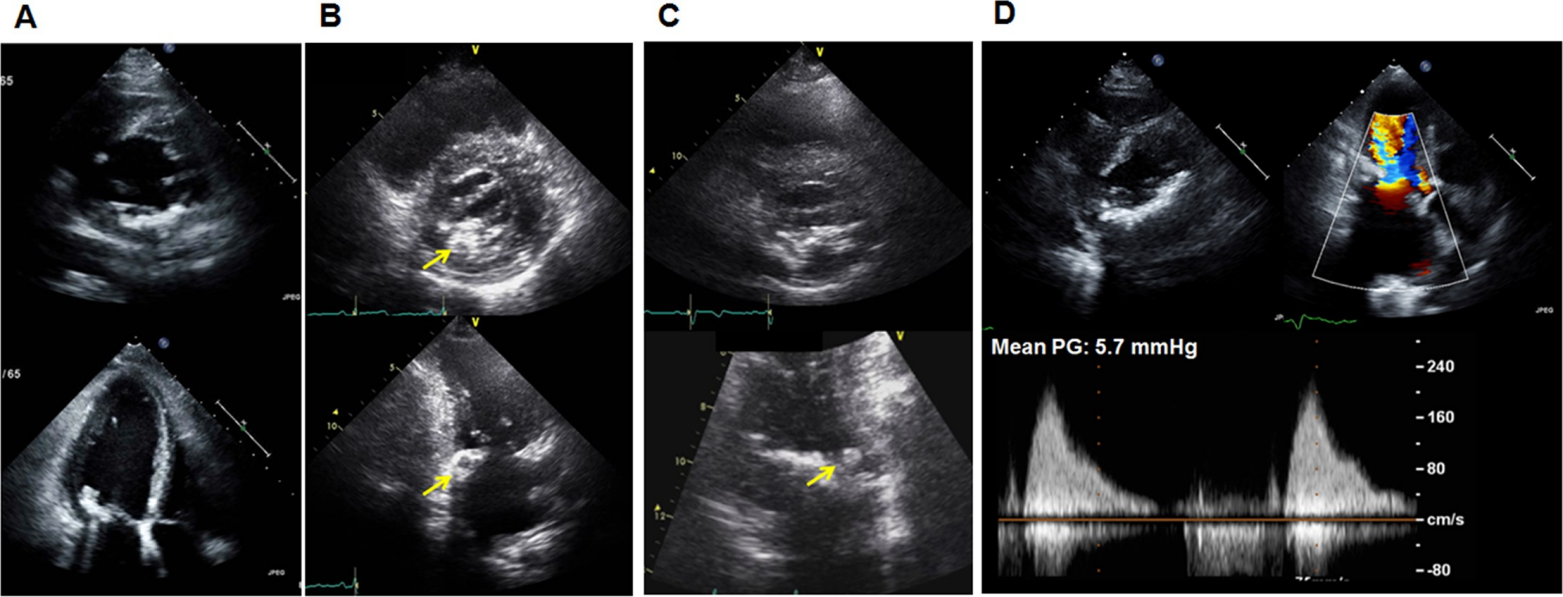
Representative cases. **A)** MAC in a patient without previous history of stroke: MAC is encircling the posterior mitral annulus without a mobile component, caseous necrosis, or functional stenosis. The MAC score of the patient is 0. **B)** MAC in a patient with previous history of stroke: Extent of MAC is similar to that in patient A; however, caseous necrosis of MAC (large, round, echodense mass with central areas of echoucencies) is noted at the posterior mitral annulus. The MAC score of the patient is 1. The arrow indicates caseous necrosis of MAC. **C)** MAC in a patient with previous of history of stroke: MAC is encircling the posterior annulus and has an attached mobile component. The MAC score of the patient is 1. The arrow indicates mobile components of MAC. **D)** MAC in a patient with previous of history of stroke: Flow acceleration is observed across the MV, and MAC is the encircling posterior annulus. Doppler image shows increased MDPG (5.7 mmHg, HR: 50 bpm). The MAC score of the patient is 1.

All patients underwent comprehensive two-dimensional echocardiography with Doppler. Chamber size and diastolic function were assessed according to current guidelines [22,23]. MAC was defined as an intense echocardiographic-producing structure with highly reflective characteristics that was located at the junction of the atrioventricular groove and the posterior or anterior mitral leaflet on parasternal long axis, apical 4- or 2-chamber, or parasternal short axis view, as described in previous study [11,18]. Characteristics of MAC were assessed comprehensively on the basis of five parameters–thickness, extent of MAC, presence of mobility, presence of typical echodense mass with central areas of echolucencies suggesting caseous necrosis, and functional stenosis. The thickness of MAC was measured from the leading anterior to the trailing posterior edge at its greatest width [18]. Extent of MAC was assessed by involvement of the posterior or anterior annulus only or bilateral annuli. Mobility of MAC was defined as any portion of MAC with independent mobility from the mitral annulus. Caseous necrosis of MAC was defined as in previous studies–large, round, echodense mass with smooth borders situated in the periannular region, without acoustic shadowing artifacts and containing central areas of echolucencies resembling liquefaction [24,25]. Continuous-wave Doppler was used to measure peak and mean transvalvular mitral gradients across the mitral valve to determine presence of functional stenosis. Functional mitral stenosis was diagnosed if increased transvalvular mean gradient (≥ 5 mmHg) was associated with MAC without severely restricted leaflet tips [26]. Fig 1 shows representative echocardiographic images of MAC in patients with or without previous history of stroke. Characteristics of MAC were scored according to presence of typical echodense mass with central areas of echolucencies suggesting caseous necrosis, mobility, and functional mitral stenosis (from minimal 0 to maximal 3 points).

### Statistical analysis

Clinical and echocardiographic characteristics were compared between groups 1 and 2. Continuous variables were recorded as mean ± SD and categorical variables were reported as frequency and percentage. Differences between two groups were analyzed by Student’s t-test for continuous variables and chi square test for categorical variables. Normality was evaluated using the Shapiro-Wilk W test. Sex and comorbidities including hypertension, diabetes, dyslipidemia, atrial fibrillation, a history of infective endocarditis, and MAC characteristics were included in multivariate models. Incremental value was assessed by comparing the global χ2 value for each model. A two-sided P value < 0.05 was accepted as indicating statistical significance. All data were analyzed using SPSS version 22.0 (SPSS, Chicago, IL).

## Results

### Clinical characteristics

Table 1 shows the baseline clinical characteristics of patients. Group 2 had a higher incidence of male patients, and patients were more likely to have diabetes mellitus, dyslipidemia, atrial fibrillation and a history of infective endocarditis compared to group 1. Mean age and blood pressure did not differ between the two groups. Mean value of eGFR and prevalence of end stage renal diseases were similar in the two groups.

**Table 1:** Baseline characteristics.

	Group 1 (n = 366)	Group 2 (n = 94)	p-value
Age, year	74 ± 11	75 ± 10	0.244
Men, n (%)	123 (34)	46 (49)	0.005
Body mass index, kg/m^2^	23.0 ± 4.2	23.8 ± 3.7	0.666
Systolic blood pressure, mmHg	136 ± 23	138 ± 21	0.279
Diastolic blood pressure, mmHg	73 ± 13	73 ± 15	0.645
Pulse pressure, mmHg	62 ± 19	65 ± 19	0.198
Hypertension, n (%)	297 (81)	82 (88)	0.065
Diabetes mellitus, n (%)	152 (42)	49 (52)	0.042
Dyslipidemia, n (%)	165 (45)	57 (61)	0.005
Hypertrophic cardiomyopathy, n (%)	21 (6)	9 (10)	0.124
Atrial fibrillation, n (%)	89 (24)	33 (35)	0.025
Coronary artery disease n (%)	111 (30)	30 (32)	0.199
Congestive heart failure, n (%)	16 (4)	5 (5)	0.434
History of infective endocarditis, n (%)	2 (0.5)	5 (5)	0.005
End-stage renal disease, n (%)	73 (19)	18 (19)	0.519
Estimated GFR, ml/min/1.73m^2^	48 ± 29	43 ± 28	0.267

GFR, glomerular filtration rate

### Echocardiographic characteristics

Table 2 displays echocardiographic characteristics in the two groups. Left ventricular chamber size, ejection fraction, mass index, and left atrial volume index did not differ between the two groups. Group 2 showed a tendency of higher E/e’ that did not reach statistical significance. In terms of the morphological and functional characteristics of MAC, thickness and extent of MAC were similar between the two groups. Features of MAC in group 2, however, were more frequently accompanied with the presence of mobility (15% vs. 8%, p = 0.031), typical echo-dense mass with central echolucencies suggesting caseous necrosis (24% vs. 7%, p<0.001), and functional mitral stenosis (13% vs. 7%, p = 0.042) than MAC in group 1, as shown in Table 2. Group 2 had higher MAC score compared to group 1 (0.50 ± 0.77 vs. 0.23 ± 0.52, p<0.001) resulting from lower incidence of MAC score 0 and higher incidences of MAC score 1, 2, and 3 in group 2. Fig 1 shows representative cases of MAC in patients with or without previous stroke.

**Table 2:** Echocardiographic characteristics.

	Group 1 (n = 366)	Group2 (n = 94)	p-value
LV end diastolic dimension, mm	49 ± 7	50 ± 7	0.582
LV end systolic dimension, mm	33 ± 8	34 ± 8	0.873
LV ejection fraction, %	65 ± 11	62 ± 12	0.749
LV mass index, g/m^2^	119 ± 36	128 ± 50	0.602
Left atrial volume index, ml/m^2^	51 ± 22	55 ± 22	0.602
E velocity, m/s	0.94 ± 0.31	0.99 ± 0.30	0.784
A velocity, m/s	1.09 ± 0.33	1.08 ± 0.33	0.793
e’, cm/s	4.70±1.58	4.52 ± 1.47	0.814
E/e’	22 ± 9	24 ± 10	0.073
RV systolic pressure, mmHg	37 ± 15	37 ± 13	0.828
**Characteristics of MAC**			
Thickness	6.3 ± 4.2	6.3 ± 3.7	0.854
0–10 mm	301 (82)	76 (81)	0.764
10–20 mm	51 (17)	18 (19)	0.544
> 20 mm	3 (0)	0 (0)	1.000
Extent, n (%)			
Posterior annulus only	228 (63)	59(63)	0.516
Anterior annulus only	7 (2)	0 (0)	0.354
Bilateral annuli	131 (36)	35 (37)	0.810
Presence of mobility, n (%)	28 (8)	14 (15)	0.031
Caseous necrosis, n (%)	25 (7)	23 (24)	<0.001
Functional mitral stenosis, n (%)	24 (7)	12 (13)	0.042
Significant mitral regurgitation, n (%)	51 (14)	15 (16)	0.361
**MAC score**	0.23 ± 0.52	0.50 ± 0.77	<0.001
MAC score 0, n (%)	299 (82)	61 (65)	<0.001
MAC score 1, n (%)	50 (14)	21 (22)	0.038
MAC score 2, n (%)	17 (5)	10 (11)	0.027
MAC score 3, n (%)	0 (0)	2 (2)	0.005

LV, left ventricle; E, mitral inflow early diastolic filling; A, mitral inflow late diastolic filling; E/e’, ratio of mitral inflow early diastolic filling to early diastolic mitral annular velocity; RV, right ventricle; MAC, mitral annular calcification

### MAC characteristics in association with stroke

**Fig 2:**
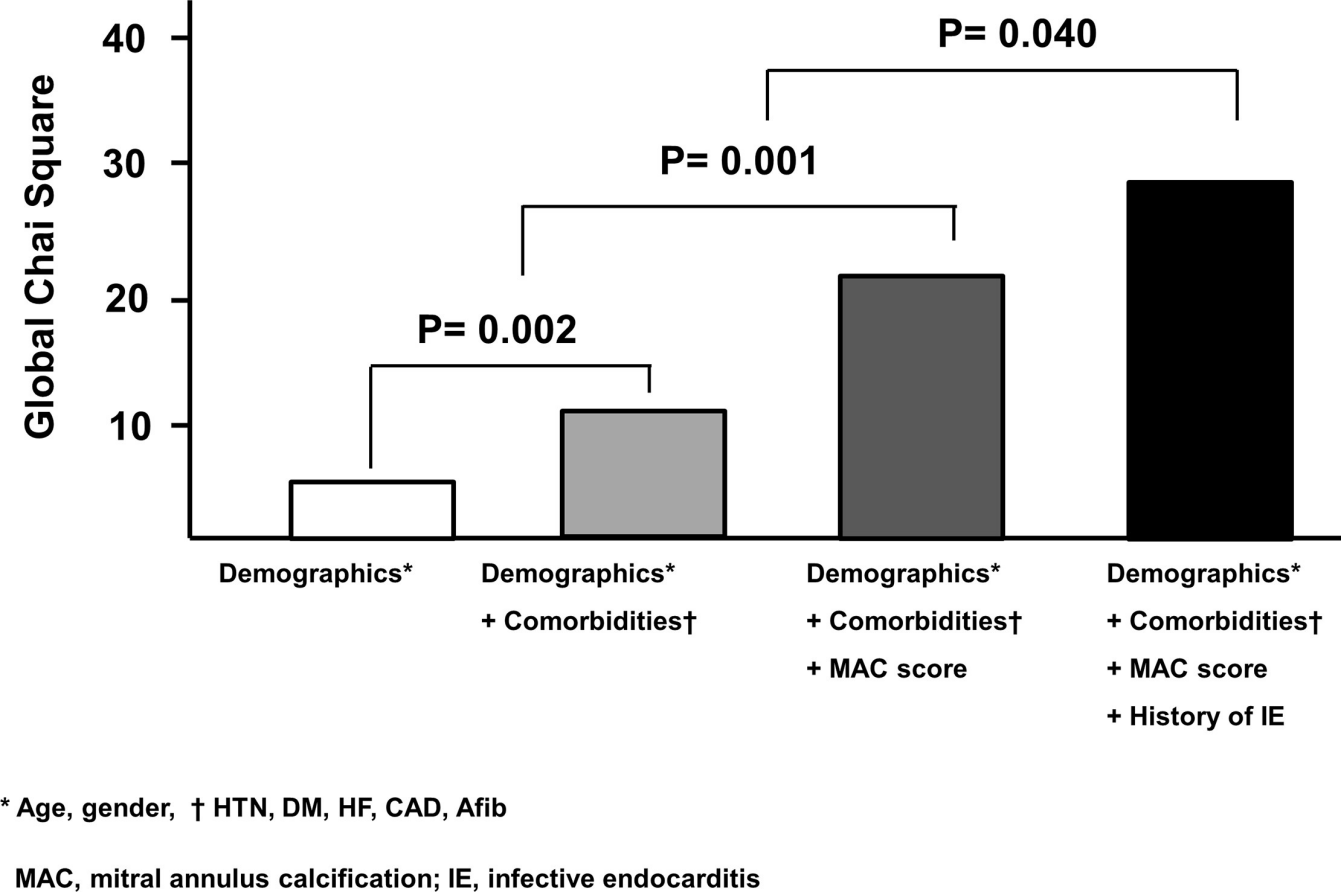
Incremental value of MAC score and a history of infective endocarditis over demographic factors and comorbidities for association with previous stroke.

Univariate analysis identified men, dyslipidemia, atrial fibrillation, history of infective endocarditis, MAC with mobility and, typical echo-dense mass with central echolucencies suggesting caseous necrosis to be associated with previous stroke (Table 3). MAC score was significantly associated with stroke (p = 0.004). On multivariate analysis, MAC score remained independently associated with previous stroke after adjustment for age, sex, dyslipidemia, atrial fibrillation, and history of infective endocarditis. Compared with MAC score 0, MAC scores 1, 2, and 3 had increased odd ratio of 2.217, 4.106, and 20.575, respectively in association with previous stroke (Table 3). MAC score had incremental value in association with stroke over demographic factors (age, sex) and comorbidities (hypertension, diabetes, congestive heart failure, coronary artery disease, atrial fibrillation) (Fig 2). History of infective endocarditis also demonstrated significant additive value in association with stroke over MAC score.

**Table 3:** Logistic regression for independent factors in association with stroke.

	Univariate			Multivariate		
	OR	95% CI	p	OR	95% CI	p
Age	1.009	0.988–1.030	0.427			
Men	1.893	1.197–2.996	0.006	2.180	1.259–3.777	0.007
Hypertension	1.784	0.903–3.525	0.095	1.989	0.894–4.425	0.102
Diabetes mellitus	1.533	0.973–2.416	0.066	1.498	0.882–4.425	0.116
Congestive heart failure	1.316	0.594–2.916	0.499			
Dyslipidemia	1.716	1.071–2.750	0.025	2.199	1.176–4.111	0.014
End-stage renal disease	0.919	0.497–1.699	0.787			
Atrial fibrillation	6.597	1.165–3.123	0.010	1.795	1.006–3.201	0.051
History of IE	10.225	1.952–53.57	0.006	8.702	1.457–51.980	0.017
Thickness of MAC	1.005	0.952–1.062	0.854			
Bilateral MAC	1.064	0.665–1.702	0.795			
Functional MS	2.072	0.967–4.442	0.061			
MAC with mobility	2.412	1.340–4.343	0.003			
Caseous necrosis	3.612	1.936–6.740	<0.001			
MAC score			0.004			<0.001
MAC score 1	2.101	1.175–3.754	0.031	2.217	1.097–4.123	0.025
MAC score 2	2.883	1.126–6.601	0.027	4.106	1.623–10.386	0.003
MAC score 3	9.803	0.986–109.828	0.064	20.575	1.642–257.7	0.019

IE, infective endocarditis; MAC, mitral annular calcification; MS, mitral stenosis

## Discussion

The principal finding of this study is that a simple MAC score, accounting for MAC mobility, typical echo-dense mass containing central echolucencies suggesting caseous necrosis, and functional mitral stenosis, was significantly associated with prior stroke, even after accounting for traditional risk factors including atrial fibrillation. The MAC score reflects functional and structural characteristics of MAC, as assessed by echocardiography, that may contribute to stroke risk. Also, previous history of infective endocarditis showed incremental value in association with stroke in addition to high risk features of MAC.

Previous community-based retrospective and prospective cohort studies reported association between MAC and stroke [11,12,27]. Framingham heart study showed in a 7-year follow-up study of 1,159 elderly patients (mean age: 70), MAC was associated with a 2.1 increased relative risk of stroke after adjustment of confounding variables including atrial fibrillation [12]. Results from a relatively younger cohort of 2,723 American Indians (mean ages: 59.2) suggest similar results; during a median 7-year follow-up, MAC was associated with a 3.12 increased risk of stroke [11]. However, conflicting results have also been published. Results from a multiethnic cohort of 1,955 subjects (mean age: 68) revealed that MAC did not show significant association with ischemic stroke [18]. In the SPAF (Stroke Prevention in Atrial Fibrillation) study, MAC failed to remain significantly associated with stroke when patients with non-rheumatic atrial fibrillation were excluded [19]. The association between MAC and stroke was questioned by investigators who suggested that MAC is merely associated with other cardiovascular risk factors for stroke, such as age, hypertension, and atrial fibrillation [18]. Indeed, pathological studies supported that MAC is a form of atherosclerosis and is closely associated with incident atrial fibrillation, carotid atherosclerosis, and aortic atheroma, which are risk factors for cardioembolic and atherothrombotic stroke [15,28–30].

Our results showed that there are certain morphological and functional characters of MAC that were significantly associated with prior stroke, supporting previous study suggesting association of MAC and stroke. To our knowledge, there were a few studies focusing on characteristics of MAC with risk of stroke. A previous cohort study showed that thickness of MAC measured by M mode was linearly associated with increased risk of stroke [12]. From our results, thickness of MAC did not show significant association with stroke. Rather, complicated characteristics of MAC reflected by MAC score, consisting of mobility, presence of typical echo-dense mass with central echolucencies suggesting caseous necrosis, and functional mitral stenosis, had incremental value in association with stroke over traditional risk factors for stroke. As shown in Fig 1, simple MAC without high risk features may not be associated with stroke and remains to be incidental findings. However, complicated MAC with many high-risk features may be a source of embolism or a more potent surrogate marker for composite risk factors for stroke and should not be neglected. Although our finding may be supporting evidence for association of MAC with stroke, due to the retrospective nature of our study, we cannot establish cause-and-effect association, and a prospective longitudinal study is needed in the future.

Interestingly, thickness of MAC did not show significant correlation with previous stroke. Results from the Framingham heart study showed that each millimeter in increased thickness of the MAC from M mode echocardiography represented a relative risk factor of 1.24 [12]. However, from our data, maximal thickness did not differ between patients with or without history of stroke. The incremental value of MAC scores may represent extensively formed MAC around the annulus and may better reflect the severity of MAC than thickness measured from echocardiography or binary classification of extent according to involved annuli, which showed nonsignificant association in multivariate analysis. Moreover, presence of functional mitral stenosis may elevate left atrial pressure and be prone to thrombus formation. The mobile characteristic of MAC from our study reflects the risk of embolism of MAC, which is consistent with previous studies [31,32]. Typical echo-dense mass with central echolucencies suggesting caseous necrosis of MAC showed significant association with previous stroke in multivariate analysis. Histologically, caseous necrosis of MAC composed of an admixture of calcium, fatty acid, and cholesterol with toothpaste-like texture with caseous transformation of inner material [24]. This variant of MAC has not been adequately appreciated, and the precise mechanism involved in liquefaction and caseation is not well understood. The clinical implication of caseous necrosis has not been investigated much. A few case reports suggest possible association between caseous necrosis of MAC and stroke [33,34]. Possible mechanisms of stroke and caseous necrosis of MAC include embolization of small calcific materials, thrombus from ulceration of the surface or by direct fistulization into the lumen of the left atrium or ventricle. Further studies including multimodality imaging study are needed to reveal the clinical significance of caseous necrosis of MAC. Our results also demonstrated that previous history of infective endocarditis had incremental value in association with stroke over traditional risk factors and MAC score. Pressman et al. suggested that MAC may act as a possible nidus of infective endocarditis and vegetation frequently occurring on calcific nodules, increasing affinity to specific bacterial species as S. aureus.^17^ Stroke from MAC = associated infective vegetation is likely to increase as the population ages and MAC is more prevalent in the elderly population.

This study has several limitations. First, a cause-and-effect relationship could not be determined due to the cross-sectional design of the current study. Prospective, longitudinal studies are needed to confirm the clinical implications of high risk morphological and functional characteristics of MAC. Our results, however, suggest possible high-risk features of MAC, and our results provide an impetus for further studies. Second, confirmation of previous stroke was based on documented medical records and previous brain imaging. As a consequence, a proportion of stroke may not have been included. As this was a retrospective study, classification of ischemic stroke could not be determined in some cases. Further studies are needed to determine if high-risk features of MAC are associated with certain types of stroke, such as embolic ischemic stroke. Third, lack of imaging modalities to assess MAC other than echocardiography may limit assessment of MAC severity. For example, computed tomography would be more accurate for assessing extent of MAC. We used five echocardiographic parameters to describe functional and structural characteristics of MAC to mitigate this limitation. In addition, we defined presence of caseous necrosis based on echocardiographic findings. Although pathologic confirmation was not performed, previous studies have shown that a typical echo-dense mass containing central areas of echolucencies at the posterior periannular region of the mitral valve on echocardiography is compatible with diagnosis of caseous necrosis from a surgical specimen [25].

## Conclusions

In conclusion, morphological and functional characteristics of MAC had incremental value in association with stroke over traditional risk factors. MAC score consisting of mobility, typical echodense mass with central areas of echolucencies suggesting caseous necrosis, and functional mitral stenosis was independently associated with stroke and may reflect stroke risk. These results build on previous prospective cohort trials by demonstrating that MAC with high risk features has incremental value in association with stroke.

## Supporting information

S1 File Data set. Data set of MAC pat.
(PDF)

## References

[1] Korn D, Desanctis RW, Sell S. Massive calcification of the mitral annulus. A clinicopathological study of fourteen cases. N Engl J Med. 1962; 267:900–9. 10.1056/NEJM196211012671802 14034804

[2] Pomerance A. Pathological and clinical study of calcification of the mitral valve ring. J Clin Pathol. 1970; 23:354–61. 10.1136/jcp.23.4.354 5430424 PMC476757

[3] Fertman MH, Wolff L. Calcification of the mitral valve. Am Heart J. 1946; 31:580–9. 10.1016/0002-8703(46)90004-x 20981944

[4] Fox CS, Vasan RS, Parise H, Levy D, O'Donnell CJ, D'Agostino RB, et al Mitral annular calcification predicts cardiovascular morbidity and mortality—The Framingham Heart Study. Circulation. 2003; 107:1492–6. 10.1161/01.Cir.0000058168.26163.Bc WOS:000181764600009 12654605

[5] Gondrie MJA, van der Graaf Y, Jacobs PC, Oen AL, Mali WPTM, Grp PS. The association of incidentally detected heart valve calcification with future cardiovascular events. European Radiology. 2011; 21:963–73. 10.1007/s00330-010-1995-0 WOS:000289291100009 21058039 PMC3072500

[6] Volzke H, Haring R, Lorbeer R, Wallaschofski H, Reffelmann T, Empen K, et al Heart valve sclerosis predicts all-cause and cardiovascular mortality. Atherosclerosis. 2010; 209:606–10. 10.1016/j.atherosclerosis.2009.10.030 WOS:000276158000048 19922935

[7] Ramaraj R, Manrique C, Hashemzadeh M, Movahed MR. Mitral annulus calcification is independently associated with all-cause mortality. Experimental & Clinical Cardiology. 2013; 18:E5–E7. WOS:000319219000002 24294050 PMC3716491

[8] Atar S, Jeon DS, Luo H, Siegel RJ. Mitral annular calcification: a marker of severe coronary artery disease in patients under 65 years old. Heart. 2003; 89:161–4. 10.1136/heart.89.2.161 WOS:000180611700016 12527666 PMC1767558

[9] Lu ML, Gupta S, Romero-Corral A, Matejkova M, De Venecia T, Obasare E, et al Cardiac Calcifications on Echocardiography Are Associated with Mortality and Stroke. J Am Soc Echocardiogr. 2016; 29:1171–8. 10.1016/j.echo.2016.08.020 27742243

[10] Gardin JM, McCleland R, Kitzman D, Lima JAC, Bommer W, Klopfenstein HS, et al M-mode echocardiographic predictors at six- to seven-year incidence of coronary heart disease, stroke, congestive heart failure, and mortality in an elderly cohort (the Cardiovascular Health Study). American Journal of Cardiology. 2001; 87:1051–7. WOS:000168352700004 10.1016/s0002-9149(01)01460-6 11348601

[11] Kizer JR, Wiebers DO, Whisnant JP, Galloway JM, Welty TK, Lee ET, et al Mitral annular calcification, aortic valve sclerosis, and incident stroke in adults free of clinical cardiovascular disease: the Strong Heart Study. Stroke. 2005; 36:2533–7. 10.1161/01.STR.0000190005.09442.ad 16254219

[12] Benjamin EJ, Plehn JF, D'Agostino RB, Belanger AJ, Comai K, Fuller DL, et al Mitral annular calcification and the risk of stroke in an elderly cohort. N Engl J Med. 1992; 327:374–9. 10.1056/NEJM199208063270602 1625711

[13] Allison MA, Cheung P, Criqui MH, Langer RD, Wright CM. Mitral and aortic annular calcification are highly associated with systemic calcified atherosclerosis. Circulation. 2006; 113:861–6. 10.1161/CIRCULATIONAHA.105.552844 16461818

[14] Adler Y, Vaturi M, Fink N, Tanne D, Shapira Y, Weisenberg D, et al Association between mitral annulus calcification and aortic atheroma: a prospective transesophageal echocardiographic study. Atherosclerosis. 2000; 152:451–6. ISI:000089651000020 10.1016/s0021-9150(99)00497-9 10998474

[15] O'Neal WT, Efird JT, Nazarian S, Alonso A, Heckbert SR, Soliman EZ. Mitral annular calcification and incident atrial fibrillation in the Multi-Ethnic Study of Atherosclerosis. Europace. 2015; 17:358–63. ISI:000351605000005 10.1093/europace/euu265 25341740 PMC4415068

[16] O'Neal WT, Efird JT, Nazarian S, Alonso A, Michos ED, Szklo M, et al Mitral annular calcification progression and the risk of atrial fibrillation: results from MESA. European Heart Journal-Cardiovascular Imaging. 2018; 19:279–84. ISI:000427282100008 10.1093/ehjci/jex093 28460029 PMC5837370

[17] Pressman GS, Rodriguez-Ziccardi M, Gartman CH, Obasare E, Melendres E, Arguello V, et al Mitral Annular Calcification as a Possible Nidus for Endocarditis: A Descriptive Series with Bacteriological Differences Noted. Journal of the American Society of Echocardiography. 2017; 30:572–8. ISI:000405452500009 10.1016/j.echo.2017.01.016 28366636

[18] Kohsaka S, Jin Z, Rundek T, Boden-Albala B, Homma S, Sacco RL, et al Impact of mitral annular calcification on cardiovascular events in a multiethnic community: the Northern Manhattan Study. JACC Cardiovasc Imaging. 2008; 1:617–23. 10.1016/j.jcmg.2008.07.006 19356491 PMC2847358

[19] Vaziri S, Bikkina M, Levy D. Predictors of thromboembolism in atrial fibrillation. Ann Intern Med. 1992; 117:89–90. 10.7326/0003-4819-117-1-89 1596053

[20] Aho K, Harmsen P, Hatano S, Marquardsen J, Smirnov VE, Strasser T. Cerebrovascular disease in the community: results of a WHO collaborative study. Bull World Health Organ. 1980; 58:113–30. 6966542 PMC2395897

[21] Eknoyan G, Levin NW. K/DOQI clinical practice guidelines for chronic kidney disease: Evaluation, classification, and stratification—Foreword. American Journal of Kidney Diseases. 2002; 39:S14–S266. 10.1053/ajkd.2002.30939 WOS:000174176800001 11904577

[22] Lang RM, Badano LP, Mor-Avi V, Afilalo J, Armstrong A, Ernande L, et al Recommendations for cardiac chamber quantification by echocardiography in adults: an update from the American Society of Echocardiography and the European Association of Cardiovascular Imaging. J Am Soc Echocardiogr. 2015; 28:1–39 e14. 10.1016/j.echo.2014.10.003 25559473

[23] Nagueh SF, Smiseth OA, Appleton CP, Byrd BF 3rd, Dokainish H, Edvardsen T, et al Recommendations for the Evaluation of Left Ventricular Diastolic Function by Echocardiography: An Update from the American Society of Echocardiography and the European Association of Cardiovascular Imaging. J Am Soc Echocardiogr. 2016; 29:277–314. 10.1016/j.echo.2016.01.011 27037982

[24] Deluca G, Correale M, Ieva R, Del Salvatore B, Gramenzi S, Di Biase M. The incidence and clinical course of caseous calcification of the mitral annulus: A prospective echocardiographic study. Journal of the American Society of Echocardiography. 2008; 21:828–33. WOS:000257205300011 10.1016/j.echo.2007.12.004 18222637

[25] Harpaz D, Auerbach I, Vered Z, Motro M, Tobar A, Rosenblatt S. Caseous calcification of the mitral annulus: a neglected, unrecognized diagnosis. J Am Soc Echocardiogr. 2001; 14:825–31. 10.1067/mje.2001.111877 11490332

[26] Akram MR, Chan T, McAuliffe S, Chenzbraun A. Non-rheumatic annular mitral stenosis: prevalence and characteristics. Eur J Echocardiogr. 2009; 10:103–5. 10.1093/ejechocard/jen179 18579487

[27] Furlan AJ, Craciun AR, Salcedo EE, Mellino M. Risk of stroke in patients with mitral annulus calcification. Stroke. 1984; 15:801–3. 10.1161/01.str.15.5.801 6474529

[28] Fox CS, Parise H, Vasan RS, Levy D, O'Donnell CJ, D'Agostino RB, et al Mitral annular calcification is a predictor for incident atrial fibrillation. Atherosclerosis. 2004; 173:291–4. ISI:000221755700016 10.1016/j.atherosclerosis.2003.12.018 15064104

[29] Pujadas R, Arboix A, Anguera N, Rafel J, Sagues F, Casanas R. Mitral annular calcification as a marker of complex aortic atheroma in patients with stroke of uncertain etiology. Echocardiography. 2008; 25:124–32. 10.1111/j.1540-8175.2007.00570.x 18269556

[30] Adler Y, Koren A, Fink N, Tanne D, Fusman R, Assali A, et al Association between mitral annulus calcification and carotid atherosclerotic disease. Stroke. 1998; 29:1833–7. 10.1161/01.str.29.9.1833 9731604

[31] Eicher JC, Soto FX, DeNadai L, Ressencourt O, Falcon-Eicher S, Giroud M, et al Possible association of thrombotic, nonbacterial vegetations of the mitral ring-mitral annular calcium and stroke. Am J Cardiol. 1997; 79:1712–5. 10.1016/s0002-9149(97)00233-6 9202375

[32] Lin CS, Schwartz IS, Chapman I. Calcification of the mitral annulus fibrosus with systemic embolization. A clinicopathologic study of 16 cases. Arch Pathol Lab Med. 1987; 111:411–4. 3566471

[33] Chevalier B, Reant P, Laffite S, Barandon L. Spontaneous fistulization of a caseous calcification of the mitral annulus: an exceptional cause of stroke. Eur J Cardiothorac Surg. 2011; 39:e184–5. 10.1016/j.ejcts.2011.01.038 21376613

[34] Marci M, Lo Jacono F. Mitral regurgitation due to caseous calcification of the mitral annulus: two case reports. Cases J. 2009; 2:95 10.1186/1757-1626-2-95 19178692 PMC2642793

