# Supplementary material for: Morphological and functional characteristics of mitral annular calcification and their relationship to stroke
Source: PLoS One. 2020 Jan 13;15(1):e0227753. doi: 10.1371/journal.pone.0227753 (PMC6957171; doi:10.1371/journal.pone.0227753)
Supplement: S1 File — Data set of MAC pat. (PDF) [file pone.0227753.s001.pdf]

No	MS	IE	MS_MDPG	CN2	Mob2	NewMAC_s	MAC_s2
1	No	1.0	0.00	1.00	1.0	2.00	2.00
2	No	0.0	0.00	1.00	0.0	1.00	1.00
3	No	0.0	0.00	0.00	0.0	0.00	0.00
4	Mild	0.0	0.00	0.00	1.0	1.00	1.00
5	No	0.0	0.00	0.00	0.0	0.00	0.00
6	Insignificar	0.0	0.00	1.00	0.0	1.00	1.00
7	No	0.0	0.00	0.00	0.0	0.00	0.00
8	Fuctional	0.0	0.00	1.00	0.0	1.00	1.00
9	Mild	0.0	0.00	1.00	0.0	1.00	1.00
10	Insignificar	0.0	0.00	0.00	0.0	0.00	0.00
11	Mild	0.0	0.00	0.00	0.0	0.00	0.00
12	No	0.0	0.00	1.00	1.0	2.00	2.00
13	No	0.0	0.00	0.00	0.0	0.00	0.00
14	Insignificar	0.0	0.00	0.00	0.0	0.00	0.00
15	Mild to mo	0.0	1.00	1.00	0.0	2.00	2.00
16	No	0.0	0.00	0.00	0.0	0.00	0.00
17	No	0.0	0.00	0.00	0.0	0.00	0.00
18	Insignificar	0.0	0.00	0.00	0.0	0.00	0.00
19	No	0.0	0.00	0.00	0.0	0.00	0.00
20	No	0.0	0.00	0.00	0.0	0.00	0.00
21	Insignificar	0.0	0.00	1.00	0.0	1.00	1.00
22	No	0.0	0.00	0.00	0.0	0.00	0.00
23	No	0.0	0.00	0.00	0.0	0.00	0.00
24	No	0.0	0.00	0.00	0.0	0.00	0.00
25	No	0.0	0.00	0.00	0.0	0.00	0.00
26	Moderate(	0.0	1.00	0.00	0.0	1.00	1.00
27	Insignificar	0.0	0.00	0.00	0.0	0.00	0.00
28	Functional	0.0	0.00	1.00	1.0	2.00	2.00
29	No	1.0	0.00	0.00	0.0	0.00	0.00
30	No	0.0	0.00	0.00	0.0	0.00	0.00
31	No	0.0	0.00	0.00	0.0	0.00	0.00
32	No	0.0	0.00	0.00	0.0	0.00	0.00
33	No	0.0	0.00	0.00	0.0	0.00	0.00
34	No	0.0	0.00	0.00	0.0	0.00	0.00
35	No	0.0	0.00	0.00	0.0	0.00	0.00
36	Severe	0.0	1.00	0.00	1.0	2.00	2.00
37	Mild	0.0	1.00	0.00	0.0	1.00	1.00
38	No	0.0	0.00	0.00	0.0	0.00	0.00
39	No	0.0	0.00	0.00	0.0	0.00	0.00
40	No	0.0	0.00	1.00	0.0	1.00	1.00
41	No	0.0	0.00	0.00	0.0	0.00	0.00
42	Mild to mo	0.0	0.00	0.00	1.0	1.00	1.00

43	No	0.0	0.00	0.00	0.0	0.00	0.00
44	No	0.0	0.00	0.00	0.0	0.00	0.00
45	No	0.0	0.00	0.00	0.0	0.00	0.00
46	Functional	0.0	0.00	0.00	0.0	0.00	0.00
47	No	0.0	0.00	0.00	0.0	0.00	0.00
48	No	0.0	0.00	0.00	0.0	0.00	0.00
49	No	0.0	0.00	0.00	0.0	0.00	0.00
50	Insignificar	0.0	0.00	0.00	1.0	1.00	1.00
51	No	0.0	0.00	0.00	0.0	0.00	0.00
52	No	0.0	0.00	0.00	0.0	0.00	0.00
53	No	0.0	0.00	0.00	0.0	0.00	0.00
54	Functional	1.0	0.00	1.00	1.0	2.00	2.00
55	Functional	0.0	1.00	0.00	0.0	1.00	1.00
56	No	0.0	0.00	0.00	0.0	0.00	0.00
57	Mild	0.0	0.00	0.00	0.0	0.00	0.00
58	No	0.0	0.00	0.00	0.0	0.00	0.00
59	No	0.0	0.00	1.00	1.0	2.00	2.00
60	No	1.0	0.00	1.00	0.0	1.00	1.00
61	Functional	0.0	0.00	0.00	0.0	0.00	0.00
62	No	0.0	0.00	0.00	0.0	0.00	0.00
63	Insignificar	0.0	0.00	0.00	0.0	0.00	0.00
64	No	0.0	0.00	0.00	0.0	0.00	0.00
65	Functional	0.0	1.00	1.00	0.0	2.00	2.00
66	No	0.0	0.00	0.00	0.0	0.00	0.00
67	Insignificar	0.0	0.00	0.00	0.0	0.00	0.00
68	No	0.0	0.00	0.00	1.0	1.00	1.00
69	No	0.0	0.00	1.00	0.0	1.00	1.00
70	No	0.0	0.00	0.00	0.0	0.00	0.00
71	Mild	0.0	1.00	1.00	1.0	3.00	3.00
72	No	0.0	0.00	0.00	0.0	0.00	0.00
73	No	0.0	0.00	0.00	0.0	0.00	0.00
74	No	0.0	0.00	0.00	0.0	0.00	0.00
75	Functional	0.0	0.00	0.00	0.0	0.00	0.00
76	Fuctional	0.0	1.00	0.00	1.0	2.00	2.00
77	No	0.0	0.00	0.00	0.0	0.00	0.00
78	Functional	0.0	0.00	0.00	0.0	0.00	0.00
79	No	0.0	0.00	1.00	0.0	1.00	1.00
80	Functional	0.0	0.00	0.00	1.0	1.00	1.00
81	No	0.0	0.00	0.00	0.0	0.00	0.00
82	Insignificar	0.0	0.00	0.00	0.0	0.00	0.00
83	No	0.0	0.00	0.00	0.0	0.00	0.00
84	No	0.0	0.00	0.00	0.0	0.00	0.00
85	Functional	0.0	0.00	0.00	0.0	0.00	0.00

86	Functional	0.0	0.00	0.00	0.0	0.00	0.00
87	No	1.0	0.00	0.00	0.0	0.00	0.00
88	Moderate	0.0	1.00	0.00	0.0	1.00	1.00
89	Insignificar	0.0	0.00	0.00	0.0	0.00	0.00
90	No	0.0	0.00	1.00	0.0	1.00	1.00
91	Mild	0.0	1.00	1.00	0.0	2.00	2.00
92	No	0.0	0.00	0.00	0.0	0.00	0.00
93	No	0.0	0.00	0.00	0.0	0.00	0.00
94	Insignificar	0.0	0.00	1.00	0.0	1.00	1.00
95	Severe	0.0	0.00	0.00	0.0	0.00	0.00
96	No	0.0	0.00	0.00	0.0	0.00	0.00
97	Insignificar	0.0	0.00	0.00	0.0	0.00	0.00
98	Mild	0.0	0.00	0.00	0.0	0.00	0.00
99	No	0.0	0.00	0.00	0.0	0.00	0.00
100	No	0.0	0.00	0.00	0.0	0.00	0.00
101	No	0.0	0.00	0.00	0.0	0.00	0.00
102	Moderate	0.0	1.00	1.00	1.0	3.00	3.00
103	Insignificar	0.0	0.00	0.00	0.0	0.00	0.00
104	No	0.0	0.00	0.00	0.0	0.00	0.00
105	Functional	0.0	0.00	0.00	0.0	0.00	0.00
106	Moderate	0.0	1.00	0.00	0.0	1.00	1.00
107	No	0.0	0.00	1.00	1.0	2.00	2.00
108	No	0.0	0.00	0.00	0.0	0.00	0.00
109	Insignificar	0.0	0.00	0.00	0.0	0.00	0.00
110	Insignificar	0.0	0.00	0.00	0.0	0.00	0.00
111	No	0.0	0.00	0.00	0.0	0.00	0.00
112	No	0.0	0.00	0.00	0.0	0.00	0.00
113	Functional	0.0	0.00	0.00	1.0	1.00	1.00
114	No	0.0	0.00	0.00	0.0	0.00	0.00
115	No	0.0	0.00	0.00	0.0	0.00	0.00
116	Insignificar	1.0	0.00	0.00	1.0	1.00	1.00
117	No	0.0	0.00	0.00	1.0	1.00	1.00
118	Functional	0.0	0.00	0.00	0.0	0.00	0.00
119	Functional	0.0	0.00	1.00	1.0	2.00	2.00
120	No	0.0	0.00	1.00	1.0	2.00	2.00
121	No	0.0	0.00	0.00	0.0	0.00	0.00
122	Mild	0.0	0.00	0.00	0.0	0.00	0.00
123	No	0.0	0.00	0.00	0.0	0.00	0.00
124	Functional	0.0	0.00	0.00	0.0	0.00	0.00
125	No	0.0	0.00	0.00	0.0	0.00	0.00
126	No	0.0	0.00	0.00	0.0	0.00	0.00
127	No	0.0	0.00	0.00	0.0	0.00	0.00
128	No	0.0	0.00	0.00	0.0	0.00	0.00

129	No	0.0	0.00	0.00	0.0	0.00	0.00
130	Mild	0.0	0.00	1.00	0.0	1.00	1.00
131	Moderate	0.0	1.00	0.00	0.0	1.00	1.00
132	No	0.0	0.00	0.00	0.0	0.00	0.00
133	No	0.0	0.00	0.00	0.0	0.00	0.00
134	No	0.0	0.00	0.00	0.0	0.00	0.00
135	No	0.0	0.00	1.00	1.0	2.00	2.00
136	No	0.0	0.00	0.00	0.0	0.00	0.00
137	Insignificar	0.0	0.00	0.00	0.0	0.00	0.00
138	No	0.0	0.00	0.00	0.0	0.00	0.00
139	No	0.0	0.00	0.00	0.0	0.00	0.00
140	Insignificar	0.0	0.00	0.00	0.0	0.00	0.00
141	No	0.0	0.00	0.00	0.0	0.00	0.00
142	Functional	0.0	0.00	0.00	0.0	0.00	0.00
143	Insignificar	0.0	0.00	0.00	0.0	0.00	0.00
144	No	0.0	0.00	0.00	0.0	0.00	0.00
145	No	0.0	0.00	0.00	0.0	0.00	0.00
146	No	0.0	0.00	0.00	0.0	0.00	0.00
147	Moderate	0.0	1.00	0.00	0.0	1.00	1.00
148	Insignificar	0.0	0.00	0.00	1.0	1.00	1.00
149	Insignificar	0.0	0.00	0.00	0.0	0.00	0.00
150	No	0.0	0.00	0.00	0.0	0.00	0.00
151	No	0.0	0.00	0.00	0.0	0.00	0.00
152	No	0.0	0.00	1.00	1.0	2.00	2.00
153	Mild	0.0	1.00	1.00	0.0	2.00	2.00
154	No	0.0	0.00	0.00	0.0	0.00	0.00
155	No	0.0	0.00	0.00	0.0	0.00	0.00
156	No	0.0	0.00	0.00	0.0	0.00	0.00
157	No	0.0	0.00	0.00	0.0	0.00	0.00
158	Functional	0.0	0.00	0.00	0.0	0.00	0.00
159	No	0.0	0.00	0.00	0.0	0.00	0.00
160	Insignificar	0.0	0.00	0.00	1.0	1.00	1.00
161	No	0.0	0.00	0.00	0.0	0.00	0.00
162	No	0.0	0.00	0.00	0.0	0.00	0.00
163	Insignificar	0.0	0.00	0.00	0.0	0.00	0.00
164	No	0.0	0.00	0.00	0.0	0.00	0.00
165	No	0.0	0.00	0.00	0.0	0.00	0.00
166	No	0.0	0.00	0.00	0.0	0.00	0.00
167	No	0.0	0.00	1.00	1.0	2.00	2.00
168	No	0.0	0.00	0.00	0.0	0.00	0.00
169	Functional	0.0	0.00	0.00	0.0	0.00	0.00
170	No	0.0	0.00	0.00	0.0	0.00	0.00
171	No	0.0	0.00	0.00	0.0	0.00	0.00

172	No	0.0	0.00	0.00	0.0	0.00	0.00
173	Mild	0.0	0.00	0.00	0.0	0.00	0.00
174	Moderate	0.0	1.00	0.00	0.0	1.00	1.00
175	No	0.0	0.00	0.00	0.0	0.00	0.00
176	No	0.0	0.00	0.00	0.0	0.00	0.00
177	No	0.0	0.00	0.00	0.0	0.00	0.00
178	No	0.0	0.00	0.00	0.0	0.00	0.00
179	No	0.0	0.00	0.00	0.0	0.00	0.00
180	Mild	0.0	0.00	1.00	0.0	1.00	1.00
181	Mild	0.0	0.00	0.00	0.0	0.00	0.00
182	Functional	0.0	0.00	0.00	1.0	1.00	1.00
183	No	0.0	0.00	0.00	0.0	0.00	0.00
184	No	0.0	0.00	0.00	0.0	0.00	0.00
185	No	0.0	0.00	0.00	0.0	0.00	0.00
186	No	0.0	0.00	0.00	0.0	0.00	0.00
187	Moderate	0.0	1.00	1.00	1.0	3.00	3.00
188	No	0.0	0.00	0.00	1.0	1.00	1.00
189	Functional	0.0	0.00	0.00	0.0	0.00	0.00
190	No	0.0	0.00	0.00	0.0	0.00	0.00
191	No	0.0	0.00	0.00	0.0	0.00	0.00
192	No	0.0	0.00	0.00	0.0	0.00	0.00
193	No	0.0	0.00	0.00	0.0	0.00	0.00
194	Insignificar	0.0	0.00	0.00	0.0	0.00	0.00
195	No	0.0	0.00	0.00	0.0	0.00	0.00
196	Functional	0.0	0.00	0.00	0.0	0.00	0.00
197	Functional	0.0	0.00	1.00	1.0	2.00	2.00
198	Functional	0.0	0.00	0.00	0.0	0.00	0.00
199	No	0.0	0.00	0.00	0.0	0.00	0.00
200	No	0.0	0.00	0.00	0.0	0.00	0.00
201	No	0.0	0.00	1.00	0.0	1.00	1.00
202	No	0.0	0.00	0.00	0.0	0.00	0.00
203	No	0.0	0.00	0.00	0.0	0.00	0.00
204	No	0.0	0.00	0.00	0.0	0.00	0.00
205	No	0.0	0.00	0.00	0.0	0.00	0.00
206	Functional	0.0	1.00	0.00	0.0	1.00	1.00
207	Insignificar	0.0	0.00	0.00	0.0	0.00	0.00
208	Mild	0.0	0.00	0.00	0.0	0.00	0.00
209	No	0.0	0.00	0.00	0.0	0.00	0.00
210	No	0.0	0.00	0.00	0.0	0.00	0.00
211	No	0.0	0.00	0.00	0.0	0.00	0.00
212	No	0.0	0.00	0.00	0.0	0.00	0.00
213	No	0.0	0.00	0.00	0.0	0.00	0.00
214	No	1.0	0.00	0.00	0.0	0.00	0.00

215	Functional	0.0	0.00	0.00	0.0	0.00	0.00
216	Functional	0.0	0.00	0.00	0.0	0.00	0.00
217	Functional	0.0	0.00	0.00	0.0	0.00	0.00
218	Functional	0.0	0.00	0.00	0.0	0.00	0.00
219	No	0.0	0.00	0.00	0.0	0.00	0.00
220	No	0.0	0.00	0.00	0.0	0.00	0.00
221	No	0.0	0.00	0.00	0.0	0.00	0.00
222	No	0.0	0.00	0.00	1.0	1.00	1.00
223	Functional	0.0	0.00	1.00	0.0	1.00	1.00
224	Mild	0.0	0.00	0.00	0.0	0.00	0.00
225	Mild	0.0	1.00	0.00	0.0	1.00	1.00
226	No	0.0	0.00	0.00	0.0	0.00	0.00
227	No	0.0	0.00	0.00	0.0	0.00	0.00
228	Mild	0.0	1.00	1.00	0.0	2.00	2.00
229	No	0.0	0.00	1.00	0.0	1.00	1.00
230	Insignificar	0.0	0.00	0.00	0.0	0.00	0.00
231	Insignificar	0.0	0.00	0.00	0.0	0.00	0.00
232	Mild	0.0	0.00	0.00	0.0	0.00	0.00
233	No	0.0	0.00	0.00	0.0	0.00	0.00
234	No	0.0	0.00	0.00	0.0	0.00	0.00
235	No	0.0	0.00	0.00	0.0	0.00	0.00
236	No	0.0	0.00	0.00	0.0	0.00	0.00
237	No	0.0	0.00	0.00	0.0	0.00	0.00
238	No	0.0	0.00	0.00	0.0	0.00	0.00
239	No	0.0	0.00	0.00	1.0	1.00	1.00
240	No	0.0	0.00	0.00	0.0	0.00	0.00
241	No	0.0	0.00	0.00	0.0	0.00	0.00
242	No	0.0	0.00	0.00	0.0	0.00	0.00
243	Insignificar	0.0	0.00	0.00	0.0	0.00	0.00
244	Insignificar	0.0	0.00	0.00	0.0	0.00	0.00
245	No	0.0	0.00	0.00	0.0	0.00	0.00
246	No	0.0	0.00	0.00	0.0	0.00	0.00
247	No	0.0	0.00	0.00	0.0	0.00	0.00
248	No	0.0	0.00	0.00	0.0	0.00	0.00
249	No	0.0	0.00	0.00	0.0	0.00	0.00
250	No	0.0	0.00	0.00	0.0	0.00	0.00
251	No	0.0	0.00	0.00	0.0	0.00	0.00
252	No	0.0	0.00	0.00	0.0	0.00	0.00
253	No	0.0	0.00	0.00	0.0	0.00	0.00
254	No	0.0	0.00	0.00	0.0	0.00	0.00
255	No	0.0	0.00	0.00	0.0	0.00	0.00
256	Insignificar	0.0	0.00	1.00	0.0	1.00	1.00
257	Mild	0.0	0.00	0.00	0.0	0.00	0.00

258 No	0.0	0.00	1.00	1.0	2.00	2.00
259 Functional	0.0	1.00	0.00	0.0	1.00	1.00
260 functional	0.0	1.00	0.00	0.0	1.00	1.00
261 Insignificar	0.0	0.00	0.00	0.0	0.00	0.00
262 Insignificar	0.0	0.00	0.00	0.0	0.00	0.00
263 Mild to mc	0.0	1.00	0.00	0.0	1.00	1.00
264 No	0.0	0.00	0.00	0.0	0.00	0.00
265 Functional	0.0	0.00	1.00	0.0	1.00	1.00
266 No	0.0	0.00	0.00	0.0	0.00	0.00
267 Functional	0.0	0.00	0.00	0.0	0.00	0.00
268 Functional	0.0	0.00	0.00	0.0	0.00	0.00
269 Insignificar	0.0	0.00	0.00	0.0	0.00	0.00
270 No	0.0	0.00	0.00	0.0	0.00	0.00
271 No	0.0	0.00	0.00	0.0	0.00	0.00
272 No	0.0	0.00	0.00	0.0	0.00	0.00
273 No	0.0	0.00	0.00	0.0	0.00	0.00
274 No	0.0	0.00	0.00	0.0	0.00	0.00
275 No	0.0	0.00	0.00	0.0	0.00	0.00
276 No	0.0	0.00	0.00	0.0	0.00	0.00
277 No	0.0	0.00	0.00	0.0	0.00	0.00
278 No	0.0	0.00	0.00	0.0	0.00	0.00
279 No	0.0	0.00	1.00	1.0	2.00	2.00
280 Insignificar	0.0	0.00	0.00	1.0	1.00	1.00
281 Insignificar	0.0	0.00	0.00	0.0	0.00	0.00
282 No	0.0	0.00	0.00	0.0	0.00	0.00
283 No	0.0	0.00	0.00	0.0	0.00	0.00
284 No	0.0	0.00	0.00	0.0	0.00	0.00
285 Functional	0.0	0.00	0.00	0.0	0.00	0.00
286 Insignificar	0.0	0.00	0.00	0.0	0.00	0.00
287 Mild	0.0	0.00	0.00	0.0	0.00	0.00
288 No	0.0	0.00	0.00	0.0	0.00	0.00
289 No	0.0	0.00	0.00	0.0	0.00	0.00
290 No	0.0	0.00	0.00	0.0	0.00	0.00
291 No	0.0	0.00	0.00	0.0	0.00	0.00
292 No	0.0	0.00	0.00	0.0	0.00	0.00
293 No	0.0	0.00	1.00	1.0	2.00	2.00
294 Functional	0.0	0.00	0.00	1.0	1.00	1.00
295 Insignificar	0.0	0.00	0.00	0.0	0.00	0.00
296 Insignificar	0.0	0.00	0.00	0.0	0.00	0.00
297 No	0.0	0.00	0.00	0.0	0.00	0.00
298 No	0.0	0.00	1.00	1.0	2.00	2.00
299 Insignificar	0.0	0.00	0.00	0.0	0.00	0.00
300 No	0.0	0.00	0.00	0.0	0.00	0.00

301	No	0.0	0.00	0.00	0.0	0.00	0.00
302	Insignificar	0.0	0.00	0.00	0.0	0.00	0.00
303	Mild	0.0	0.00	0.00	0.0	0.00	0.00
304	No	0.0	0.00	0.00	0.0	0.00	0.00
305	No	0.0	0.00	0.00	0.0	0.00	0.00
306	No	0.0	0.00	0.00	0.0	0.00	0.00
307	No	0.0	0.00	0.00	0.0	0.00	0.00
308	No	0.0	0.00	0.00	1.0	1.00	1.00
309	No	0.0	0.00	0.00	0.0	0.00	0.00
310	Functional	0.0	0.00	0.00	0.0	0.00	0.00
311	Mild	0.0	0.00	0.00	0.0	0.00	0.00
312	No	0.0	0.00	0.00	0.0	0.00	0.00
313	Mild	0.0	0.00	0.00	0.0	0.00	0.00
314	Functional	0.0	0.00	0.00	0.0	0.00	0.00
315	Insignificar	0.0	0.00	0.00	0.0	0.00	0.00
316	No	0.0	0.00	0.00	0.0	0.00	0.00
317	No	0.0	0.00	0.00	0.0	0.00	0.00
318	No	0.0	0.00	0.00	0.0	0.00	0.00
319	No	0.0	0.00	0.00	0.0	0.00	0.00
320	No	0.0	0.00	0.00	0.0	0.00	0.00
321	No	0.0	0.00	0.00	0.0	0.00	0.00
322	No	0.0	0.00	0.00	0.0	0.00	0.00
323	No	0.0	0.00	0.00	0.0	0.00	0.00
324	Insignificar	0.0	0.00	0.00	1.0	1.00	1.00
325	No	0.0	0.00	0.00	0.0	0.00	0.00
326	No	0.0	0.00	0.00	0.0	0.00	0.00
327	Insignificar	0.0	0.00	0.00	1.0	1.00	1.00
328	Functional	0.0	0.00	1.00	0.0	1.00	1.00
329	No	0.0	0.00	0.00	0.0	0.00	0.00
330	No	0.0	0.00	0.00	0.0	0.00	0.00
331	functional	0.0	0.00	0.00	0.0	0.00	0.00
332	Functional	0.0	0.00	0.00	0.0	0.00	0.00
333	Functional	0.0	1.00	0.00	0.0	1.00	1.00
334	Functional	0.0	0.00	0.00	0.0	0.00	0.00
335	No	0.0	0.00	0.00	0.0	0.00	0.00
336	No	0.0	0.00	0.00	0.0	0.00	0.00
337	No	0.0	0.00	0.00	0.0	0.00	0.00
338	No	0.0	0.00	1.00	1.0	2.00	2.00
339	No	0.0	0.00	0.00	0.0	0.00	0.00
340	Functional	0.0	0.00	0.00	0.0	0.00	0.00
341	Functional	0.0	0.00	0.00	0.0	0.00	0.00
342	Insignificar	0.0	0.00	0.00	0.0	0.00	0.00
343	Mild	0.0	0.00	0.00	0.0	0.00	0.00

344	No	0.0	0.00	0.00	0.0	0.00	0.00
345	functional	0.0	1.00	0.00	0.0	1.00	1.00
346	Functional	0.0	1.00	0.00	0.0	1.00	1.00
347	Insignificar	0.0	0.00	0.00	0.0	0.00	0.00
348	No	0.0	0.00	0.00	0.0	0.00	0.00
349	No	0.0	0.00	0.00	0.0	0.00	0.00
350	No	0.0	0.00	0.00	0.0	0.00	0.00
351	No	0.0	0.00	0.00	0.0	0.00	0.00
352	No	0.0	0.00	0.00	0.0	0.00	0.00
353	No	0.0	0.00	0.00	0.0	0.00	0.00
354	No	0.0	0.00	0.00	0.0	0.00	0.00
355	functional	0.0	0.00	0.00	0.0	0.00	0.00
356	No	0.0	0.00	0.00	1.0	1.00	1.00
357	Functional	0.0	1.00	1.00	0.0	2.00	2.00
358	Functional	0.0	0.00	0.00	0.0	0.00	0.00
359	Mild	0.0	0.00	0.00	0.0	0.00	0.00
360	No	0.0	0.00	0.00	0.0	0.00	0.00
361	No	0.0	0.00	0.00	0.0	0.00	0.00
362	Insignificar	0.0	0.00	0.00	0.0	0.00	0.00
363	No	0.0	0.00	0.00	0.0	0.00	0.00
364	No	0.0	0.00	0.00	0.0	0.00	0.00
365	No	0.0	0.00	0.00	0.0	0.00	0.00
366	No	0.0	0.00	0.00	0.0	0.00	0.00
367	No	0.0	0.00	0.00	0.0	0.00	0.00
368	No	0.0	0.00	0.00	0.0	0.00	0.00
369	No	0.0	0.00	0.00	0.0	0.00	0.00
370	Insignificar	0.0	0.00	0.00	0.0	0.00	0.00
371	Mild to mc	0.0	0.00	0.00	0.0	0.00	0.00
372	No	0.0	0.00	0.00	0.0	0.00	0.00
373	No	0.0	0.00	0.00	1.0	1.00	1.00
374	No	0.0	0.00	0.00	1.0	1.00	1.00
375	No	0.0	0.00	0.00	0.0	0.00	0.00
376	Functional	0.0	0.00	0.00	0.0	0.00	0.00
377	Functional	0.0	0.00	0.00	0.0	0.00	0.00
378	Functional	0.0	1.00	0.00	0.0	1.00	1.00
379	Insignificar	0.0	0.00	0.00	0.0	0.00	0.00
380	Insignificar	0.0	0.00	0.00	0.0	0.00	0.00
381	Mild	0.0	0.00	0.00	0.0	0.00	0.00
382	No	0.0	0.00	0.00	0.0	0.00	0.00
383	No	0.0	0.00	0.00	0.0	0.00	0.00
384	No	0.0	0.00	0.00	0.0	0.00	0.00
385	No	0.0	0.00	0.00	0.0	0.00	0.00
386	Mild	0.0	0.00	0.00	0.0	0.00	0.00

387 Functional	0.0	0.00	0.00	0.0	0.00	0.00
388 No	0.0	0.00	0.00	0.0	0.00	0.00
389 Funcional	0.0	0.00	1.00	1.0	2.00	2.00
390 No	0.0	0.00	0.00	0.0	0.00	0.00
391 Insignificar	0.0	0.00	0.00	0.0	0.00	0.00
392 Mild	0.0	0.00	0.00	0.0	0.00	0.00
393 No	0.0	0.00	0.00	0.0	0.00	0.00
394 No	0.0	0.00	0.00	0.0	0.00	0.00
395 No	0.0	0.00	0.00	0.0	0.00	0.00
396 No	0.0	0.00	0.00	0.0	0.00	0.00
397 No	0.0	0.00	0.00	0.0	0.00	0.00
398 No	0.0	0.00	0.00	0.0	0.00	0.00
399 No	0.0	0.00	0.00	0.0	0.00	0.00
400 Functional	0.0	0.00	0.00	0.0	0.00	0.00
401 Insignificar	0.0	0.00	0.00	0.0	0.00	0.00
402 Mild to mc	0.0	1.00	0.00	0.0	1.00	1.00
403 No	0.0	0.00	0.00	0.0	0.00	0.00
404 No	0.0	0.00	0.00	0.0	0.00	0.00
405 No	0.0	0.00	0.00	0.0	0.00	0.00
406 Insignificar	0.0	0.00	0.00	0.0	0.00	0.00
407 No	0.0	0.00	0.00	0.0	0.00	0.00
408 No	0.0	0.00	0.00	0.0	0.00	0.00
409 Insignificar	0.0	0.00	1.00	0.0	1.00	1.00
410 No	0.0	0.00	0.00	0.0	0.00	0.00
411 No	0.0	0.00	0.00	0.0	0.00	0.00
412 No	0.0	0.00	0.00	0.0	0.00	0.00
413 No	0.0	0.00	0.00	0.0	0.00	0.00
414 Insignificar	0.0	0.00	0.00	0.0	0.00	0.00
415 Insignificar	0.0	0.00	0.00	1.0	1.00	1.00
416 Functional	0.0	0.00	0.00	1.0	1.00	1.00
417 Functional	0.0	0.00	0.00	0.0	0.00	0.00
418 functional	0.0	0.00	0.00	0.0	0.00	0.00
419 No	0.0	0.00	0.00	0.0	0.00	0.00
420 No	0.0	0.00	0.00	0.0	0.00	0.00
421 No	0.0	0.00	0.00	0.0	0.00	0.00
422 Insignificar	0.0	0.00	0.00	1.0	1.00	1.00
423 Fuctional	0.0	1.00	0.00	0.0	1.00	1.00
424 No	0.0	0.00	0.00	0.0	0.00	0.00
425 Functional	0.0	0.00	0.00	1.0	1.00	1.00
426 Functional	0.0	1.00	0.00	0.0	1.00	1.00
427 No	0.0	0.00	0.00	0.0	0.00	0.00
428 Functional	0.0	0.00	0.00	0.0	0.00	0.00
429 No	0.0	0.00	0.00	0.0	0.00	0.00

430	No	0.0	0.00	0.00	0.0	0.00	0.00
431	No	0.0	0.00	0.00	0.0	0.00	0.00
432	No	0.0	0.00	0.00	0.0	0.00	0.00
433	No	0.0	0.00	0.00	0.0	0.00	0.00
434	No	0.0	0.00	1.00	0.0	1.00	1.00
435	Functional	0.0	1.00	0.00	1.0	2.00	2.00
436	No	0.0	0.00	0.00	0.0	0.00	0.00
437	No	0.0	0.00	0.00	0.0	0.00	0.00
438	No	0.0	0.00	0.00	0.0	0.00	0.00
439	No	0.0	0.00	0.00	0.0	0.00	0.00
440	Insignificar	0.0	0.00	0.00	0.0	0.00	0.00
441	No	0.0	0.00	0.00	0.0	0.00	0.00
442	No	0.0	0.00	0.00	0.0	0.00	0.00
443	No	0.0	0.00	0.00	0.0	0.00	0.00
444	Insignificar	0.0	0.00	0.00	0.0	0.00	0.00
445	Mild	0.0	0.00	0.00	0.0	0.00	0.00
446	Insignificar	0.0	0.00	0.00	0.0	0.00	0.00
447	No	0.0	0.00	0.00	0.0	0.00	0.00
448	Functional	0.0	0.00	0.00	0.0	0.00	0.00
449	No	0.0	0.00	0.00	0.0	0.00	0.00
450	Functional	0.0	1.00	0.00	0.0	1.00	1.00
451	No	0.0	0.00	0.00	0.0	0.00	0.00
452	No	0.0	0.00	0.00	0.0	0.00	0.00
453	No	0.0	0.00	0.00	0.0	0.00	0.00
454	No	0.0	0.00	0.00	1.0	1.00	1.00
455	No	0.0	0.00	0.00	0.0	0.00	0.00
456	No	0.0	0.00	0.00	0.0	0.00	0.00
457	No	0.0	0.00	0.00	0.0	0.00	0.00
458	No	0.0	0.00	0.00	0.0	0.00	0.00
459	Insignificar	0.0	0.00	0.00	1.0	1.00	1.00
460	functional	0.0	0.00	0.00	0.0	0.00	0.00

MACgr	A.fib_1	Sex	Sex_Cat	CVA_gr	HCM	Location	Loc_code
3	0.0	F	0.00	1.0	0.0	P	3.00
3	1.0	M	1.00	1.0	0.0	B	2.00
1	0.0	M	1.00	1.0	0.0	P	3.00
3	0.0	M	1.00	1.0	0.0	B	2.00
1	1.0	M	1.00	1.0	0.0	P	3.00
2	0.0	M	1.00	1.0	0.0	P	3.00
1	0.0	F	0.00	1.0	0.0	P	3.00
1	0.0	M	1.00	1.0	0.0	P	3.00
2	0.0	M	1.00	1.0	0.0	P	3.00
2	0.0	F	0.00	1.0	0.0	P	3.00
2	0.0	M	1.00	1.0	0.0	B	2.00
3	0.0	F	0.00	1.0	0.0	P	3.00
2	0.0	F	0.00	1.0	0.0	P	3.00
2	0.0	F	0.00	1.0	0.0	P	3.00
2	0.0	F	0.00	1.0	0.0	P	3.00
2	1.0	M	1.00	1.0	0.0	P	3.00
1	1.0	M	1.00	1.0	0.0	P	3.00
1	0.0	F	0.00	1.0	0.0	P	3.00
1	1.0	M	1.00	1.0	0.0	P	3.00
1	1.0	M	1.00	1.0	0.0	P	3.00
3	0.0	F	0.00	1.0	0.0	P	3.00
1	0.0	M	1.00	1.0	0.0	P	3.00
1	1.0	M	1.00	1.0	0.0	P	3.00
2	1.0	F	0.00	1.0	0.0	P	3.00
1	1.0	M	1.00	1.0	0.0	P	3.00
3	0.0	M	1.00	1.0	0.0	B	2.00
2	0.0	F	0.00	1.0	1.0	P	3.00
3	0.0	M	1.00	1.0	0.0	B	2.00
2	0.0	M	1.00	1.0	0.0	B	2.00
2	0.0	M	1.00	1.0	0.0	B	2.00
1	1.0	F	0.00	1.0	0.0	P	3.00
1	0.0	F	0.00	1.0	1.0	P	3.00
1	0.0	F	0.00	1.0	0.0	P	3.00
1	0.0	F	0.00	1.0	0.0	P	3.00
1	0.0	F	0.00	1.0	0.0	P	3.00
3	0.0	F	0.00	1.0	0.0	B	2.00
2	1.0	M	1.00	1.0	0.0	B	2.00
2	1.0	F	0.00	1.0	0.0	B	2.00
1	0.0	M	1.00	1.0	0.0	P	3.00
2	0.0	M	1.00	1.0	0.0	B	2.00
1	1.0	F	0.00	1.0	1.0	B	2.00
2	0.0	F	0.00	1.0	0.0	P	3.00

1	0.0	M	1.00	1.0	0.0	P	3.00
2	0.0	F	0.00	1.0	0.0	P	3.00
1	0.0	M	1.00	1.0	0.0	P	3.00
2	1.0	M	1.00	1.0	0.0	P	3.00
1	0.0	F	0.00	1.0	0.0	P	3.00
1	1.0	M	1.00	1.0	0.0	P	3.00
3	1.0	F	0.00	1.0	1.0	B	2.00
2	0.0	M	1.00	1.0	#NULL!	P	3.00
1	0.0	M	1.00	1.0	0.0	P	3.00
1	0.0	F	0.00	1.0	0.0	P	3.00
1	0.0	M	1.00	1.0	0.0	P	3.00
3	0.0	F	0.00	1.0	0.0	B	2.00
3	1.0	F	0.00	1.0	0.0	B	2.00
0	1.0	M	1.00	1.0	0.0	P	3.00
2	1.0	M	1.00	1.0	0.0	P	3.00
2	1.0	F	0.00	1.0	1.0	P	3.00
2	1.0	M	1.00	0.0	0.0	B	2.00
3	0.0	M	1.00	1.0	0.0	B	2.00
2	1.0	F	0.00	1.0	1.0	B	2.00
1	1.0	M	1.00	1.0	0.0	P	3.00
1	0.0	M	1.00	1.0	0.0	B	2.00
2	1.0	F	0.00	1.0	1.0	B	2.00
2	0.0	F	0.00	1.0	0.0	B	2.00
2	1.0	M	1.00	1.0	0.0	B	2.00
2	0.0	F	0.00	1.0	0.0	B	2.00
3	1.0	M	1.00	1.0	0.0	B	2.00
2	1.0	F	0.00	1.0	0.0	B	2.00
1	0.0	F	0.00	1.0	0.0	B	2.00
3	0.0	F	0.00	1.0	1.0	B	2.00
1	0.0	M	1.00	1.0	0.0	P	3.00
1	0.0	F	0.00	1.0	1.0	P	3.00
1	1.0	M	1.00	1.0	0.0	P	3.00
2	1.0	M	1.00	1.0	0.0	P	3.00
3	0.0	M	1.00	1.0	0.0	B	2.00
1	0.0	M	1.00	1.0	0.0	P	3.00
1	0.0	F	0.00	1.0	0.0	P	3.00
2	1.0	M	1.00	1.0	0.0	B	2.00
2	0.0	F	0.00	1.0	0.0	P	3.00
1	0.0	M	1.00	1.0	0.0	P	3.00
2	1.0	F	0.00	1.0	0.0	P	3.00
1	1.0	F	0.00	1.0	0.0	P	3.00
1	0.0	M	1.00	1.0	0.0	P	3.00
3	0.0	F	0.00	1.0	0.0	P	3.00

3	0.0	F	0.00	1.0	0.0	B	2.00
2	1.0	F	0.00	1.0	0.0	P	3.00
2	0.0	F	0.00	1.0	#NULL!	B	2.00
1	0.0	M	1.00	0.0	0.0	P	3.00
3	0.0	F	0.00	0.0	0.0	B	2.00
2	0.0	F	0.00	0.0	#NULL!	B	2.00
1	0.0	M	1.00	0.0	1.0	P	3.00
1	0.0	F	0.00	0.0	0.0	P	3.00
2	0.0	F	0.00	0.0	0.0	B	2.00
2	0.0	F	0.00	0.0	0.0	B	2.00
1	0.0	M	1.00	0.0	0.0	P	3.00
2	0.0	M	1.00	0.0	0.0	P	3.00
2	1.0	M	1.00	0.0	0.0	B	2.00
1	0.0	M	1.00	0.0	0.0	P	3.00
1	1.0	F	0.00	0.0	0.0	P	3.00
3	0.0	M	1.00	0.0	0.0	B	2.00
3	0.0	F	0.00	0.0	0.0	B	2.00
3	0.0	F	0.00	0.0	0.0	B	2.00
1	0.0	M	1.00	0.0	0.0	P	3.00
2	0.0	F	0.00	0.0	0.0	P	3.00
2	0.0	M	1.00	0.0	0.0	B	2.00
2	0.0	M	1.00	0.0	0.0	P	3.00
1	0.0	M	1.00	0.0	0.0	P	3.00
1	1.0	F	0.00	0.0	0.0	A	1.00
1	0.0	F	0.00	0.0	0.0	B	2.00
1	1.0	M	1.00	0.0	0.0	P	3.00
1	1.0	M	1.00	0.0	0.0	P	3.00
3	1.0	F	0.00	0.0	0.0	B	2.00
2	0.0	F	0.00	0.0	0.0	P	3.00
1	0.0	F	0.00	0.0	0.0	P	3.00
2	0.0	M	1.00	0.0	0.0	B	2.00
3	0.0	F	0.00	0.0	0.0	B	2.00
1	0.0	M	1.00	0.0	0.0	P	3.00
3	0.0	F	0.00	0.0	1.0	B	2.00
3	0.0	F	0.00	0.0	0.0	B	2.00
3	0.0	M	1.00	0.0	0.0	B	2.00
3	1.0	M	1.00	0.0	0.0	B	2.00
2	0.0	M	1.00	0.0	0.0	P	3.00
1	0.0	M	1.00	0.0	0.0	B	2.00
2	0.0	F	0.00	0.0	0.0	B	2.00
1	0.0	M	1.00	0.0	0.0	P	3.00
2	1.0	F	0.00	0.0	0.0	P	3.00
1	0.0	F	0.00	0.0	0.0	P	3.00

3	0.0	M	1.00	0.0	0.0	P	3.00
3	0.0	F	0.00	0.0	0.0	B	2.00
1	0.0	M	1.00	0.0	0.0	B	2.00
0	0.0	M	1.00	0.0	0.0	B	2.00
1	0.0	F	0.00	0.0	0.0	P	3.00
1	0.0	F	0.00	0.0	0.0	P	3.00
0	0.0	F	0.00	0.0	0.0	B	2.00
1	0.0	F	0.00	0.0	0.0	P	3.00
2	0.0	M	1.00	0.0	0.0	B	2.00
1	0.0	M	1.00	0.0	1.0	P	3.00
3	1.0	M	1.00	0.0	0.0	P	3.00
1	0.0	M	1.00	0.0	0.0	P	3.00
1	1.0	M	1.00	0.0	0.0	P	3.00
2	0.0	F	0.00	0.0	0.0	P	3.00
1	0.0	F	0.00	0.0	0.0	P	3.00
1	0.0	F	0.00	0.0	0.0	P	3.00
2	0.0	F	0.00	0.0	0.0	P	3.00
3	0.0	F	0.00	0.0	0.0	P	3.00
2	1.0	M	1.00	0.0	0.0	B	2.00
3	0.0	F	0.00	0.0	0.0	P	3.00
1	0.0	M	1.00	0.0	0.0	P	3.00
1	0.0	F	0.00	0.0	0.0	P	3.00
1	0.0	M	1.00	0.0	0.0	P	3.00
3	0.0	M	1.00	0.0	0.0	B	2.00
2	0.0	M	1.00	0.0	0.0	B	2.00
1	0.0	M	1.00	0.0	0.0	P	3.00
2	0.0	F	0.00	0.0	1.0	P	3.00
1	0.0	F	0.00	0.0	0.0	P	3.00
1	0.0	M	1.00	0.0	0.0	P	3.00
2	0.0	F	0.00	0.0	0.0	B	2.00
2	0.0	F	0.00	0.0	0.0	B	2.00
3	0.0	M	1.00	0.0	0.0	P	3.00
1	0.0	M	1.00	0.0	0.0	P	3.00
1	0.0	M	1.00	0.0	0.0	P	3.00
2	1.0	F	0.00	0.0	0.0	P	3.00
2	1.0	F	0.00	0.0	0.0	P	3.00
2	0.0	F	0.00	0.0	0.0	P	3.00
2	0.0	M	1.00	0.0	#NULL!	P	3.00
2	0.0	M	1.00	1.0	0.0	P	3.00
2	0.0	M	1.00	0.0	0.0	P	3.00
3	0.0	F	0.00	0.0	0.0	P	3.00
1	0.0	F	0.00	0.0	1.0	P	3.00
1	0.0	F	0.00	0.0	0.0	P	3.00

1	0.0	M	1.00	0.0	0.0	B	2.00
0	0.0	M	1.00	0.0	0.0	B	2.00
2	0.0	M	1.00	0.0	0.0	B	2.00
#NULL!	0.0	F	0.00	0.0	1.0	P	3.00
2	0.0	F	0.00	0.0	0.0	P	3.00
1	0.0	F	0.00	0.0	0.0	P	3.00
1	1.0	M	1.00	0.0	0.0	P	3.00
1	0.0	M	1.00	0.0	0.0	P	3.00
2	1.0	F	0.00	0.0	0.0	B	2.00
2	0.0	M	1.00	0.0	0.0	B	2.00
2	0.0	F	0.00	0.0	0.0	P	3.00
1	0.0	F	0.00	0.0	0.0	P	3.00
1	1.0	M	1.00	0.0	0.0	P	3.00
1	1.0	F	0.00	0.0	1.0	P	3.00
1	0.0	M	1.00	0.0	#NULL!	P	3.00
2	0.0	F	0.00	1.0	#NULL!	B	2.00
2	0.0	F	0.00	0.0	0.0	B	2.00
2	0.0	M	1.00	0.0	0.0	B	2.00
0	0.0	M	1.00	0.0	0.0	B	2.00
2	0.0	F	0.00	0.0	1.0	P	3.00
1	0.0	M	1.00	0.0	0.0	P	3.00
1	0.0	F	0.00	0.0	0.0	B	2.00
2	0.0	F	0.00	0.0	0.0	P	3.00
1	0.0	F	0.00	0.0	0.0	P	3.00
1	0.0	M	1.00	0.0	0.0	A	1.00
3	0.0	F	0.00	0.0	1.0	B	2.00
3	1.0	F	0.00	0.0	0.0	B	2.00
2	0.0	F	0.00	0.0	0.0	B	2.00
2	1.0	F	0.00	0.0	0.0	B	2.00
1	0.0	F	0.00	0.0	1.0	P	3.00
1	1.0	M	1.00	0.0	0.0	P	3.00
1	0.0	M	1.00	0.0	0.0	P	3.00
1	0.0	M	1.00	0.0	0.0	P	3.00
1	0.0	M	1.00	0.0	0.0	P	3.00
3	0.0	F	0.00	0.0	0.0	P	3.00
2	0.0	F	0.00	0.0	0.0	P	3.00
2	0.0	F	0.00	0.0	0.0	P	3.00
1	1.0	F	0.00	0.0	0.0	P	3.00
1	0.0	F	0.00	0.0	0.0	P	3.00
2	1.0	F	0.00	0.0	0.0	P	3.00
1	1.0	F	0.00	0.0	0.0	P	3.00
1	0.0	M	1.00	0.0	0.0	P	3.00
2	0.0	M	1.00	0.0	0.0	B	2.00

1	1.0	M	1.00	0.0	0.0	B	2.00
3	0.0	F	0.00	0.0	0.0	B	2.00
1	0.0	F	0.00	0.0	0.0	B	2.00
2	1.0	F	0.00	0.0	0.0	B	2.00
3	0.0	F	0.00	0.0	0.0	B	2.00
1	0.0	F	0.00	0.0	0.0	B	2.00
1	0.0	M	1.00	0.0	1.0	B	2.00
1	0.0	M	1.00	0.0	0.0	P	3.00
3	0.0	F	0.00	0.0	0.0	P	3.00
2	0.0	F	0.00	0.0	0.0	P	3.00
2	0.0	M	1.00	0.0	0.0	P	3.00
2	0.0	F	0.00	0.0	0.0	P	3.00
1	0.0	F	0.00	0.0	0.0	P	3.00
3	1.0	F	0.00	1.0	0.0	B	2.00
2	0.0	F	0.00	0.0	0.0	B	2.00
2	0.0	F	0.00	0.0	0.0	B	2.00
2	1.0	M	1.00	0.0	0.0	B	2.00
2	0.0	M	1.00	0.0	0.0	B	2.00
2	1.0	F	0.00	0.0	0.0	B	2.00
2	0.0	F	0.00	0.0	0.0	B	2.00
2	1.0	F	0.00	0.0	0.0	B	2.00
1	0.0	M	1.00	0.0	0.0	B	2.00
2	0.0	M	1.00	0.0	0.0	B	2.00
1	0.0	F	0.00	0.0	0.0	p	3.00
2	0.0	F	0.00	0.0	0.0	P	3.00
1	0.0	M	1.00	0.0	0.0	P	3.00
1	0.0	M	1.00	0.0	0.0	P	3.00
1	1.0	M	1.00	0.0	1.0	P	3.00
1	1.0	F	0.00	0.0	0.0	P	3.00
1	0.0	M	1.00	0.0	0.0	P	3.00
2	1.0	F	0.00	0.0	0.0	P	3.00
1	0.0	F	0.00	0.0	0.0	P	3.00
1	1.0	F	0.00	0.0	1.0	P	3.00
2	0.0	F	0.00	0.0	1.0	P	3.00
1	0.0	F	0.00	0.0	0.0	P	3.00
2	0.0	F	0.00	0.0	0.0	P	3.00
2	1.0	F	0.00	0.0	0.0	P	3.00
2	0.0	F	0.00	0.0	0.0	P	3.00
1	1.0	F	0.00	0.0	0.0	P	3.00
2	0.0	F	0.00	0.0	1.0	P	3.00
2	0.0	F	0.00	0.0	0.0	P	3.00
2	0.0	F	0.00	1.0	0.0	B	2.00
1	0.0	F	0.00	0.0	0.0	A	1.00

3	0.0	F	0.00	0.0	0.0	B	2.00
2	0.0	F	0.00	0.0	0.0	B	2.00
2	0.0	F	0.00	0.0	0.0	B	2.00
2	0.0	F	0.00	0.0	0.0	B	2.00
2	0.0	F	0.00	0.0	0.0	B	2.00
2	1.0	F	0.00	0.0	0.0	B	2.00
2	0.0	F	0.00	0.0	0.0	B	2.00
3	0.0	F	0.00	0.0	0.0	P	3.00
1	0.0	M	1.00	0.0	0.0	P	3.00
1	0.0	F	0.00	0.0	0.0	P	3.00
2	0.0	F	0.00	0.0	0.0	P	3.00
3	0.0	F	0.00	0.0	0.0	P	3.00
2	0.0	F	0.00	0.0	0.0	P	3.00
1	0.0	F	0.00	0.0	0.0	P	3.00
3	1.0	F	0.00	0.0	0.0	P	3.00
1	0.0	F	0.00	0.0	0.0	P	3.00
1	0.0	F	0.00	0.0	0.0	P	3.00
1	1.0	F	0.00	0.0	0.0	P	3.00
1	0.0	F	0.00	0.0	0.0	P	3.00
1	0.0	M	1.00	0.0	0.0	P	3.00
2	0.0	M	1.00	0.0	0.0	P	3.00
3	0.0	F	0.00	0.0	0.0	B	2.00
2	0.0	F	0.00	0.0	0.0	B	2.00
0	0.0	F	0.00	0.0	0.0	B	2.00
2	1.0	F	0.00	0.0	0.0	B	2.00
3	1.0	M	1.00	0.0	0.0	B	2.00
1	0.0	M	1.00	0.0	0.0	P	3.00
2	1.0	M	1.00	0.0	0.0	P	3.00
2	0.0	F	0.00	0.0	0.0	P	3.00
1	0.0	M	1.00	0.0	0.0	P	3.00
1	0.0	F	0.00	0.0	0.0	P	3.00
0	0.0	F	0.00	0.0	0.0	P	3.00
1	0.0	F	0.00	0.0	0.0	P	3.00
2	0.0	F	0.00	0.0	0.0	P	3.00
1	0.0	M	1.00	0.0	0.0	P	3.00
3	0.0	F	0.00	0.0	0.0	B	2.00
3	1.0	F	0.00	0.0	0.0	B	2.00
3	0.0	M	1.00	0.0	0.0	B	2.00
1	0.0	M	1.00	0.0	0.0	B	2.00
3	0.0	M	1.00	0.0	0.0	B	2.00
3	1.0	F	0.00	0.0	0.0	P	3.00
0	0.0	F	0.00	0.0	0.0	P	3.00
1	1.0	M	1.00	0.0	0.0	P	3.00

0	0.0	M	1.00	0.0	0.0	P	3.00
2	0.0	F	0.00	0.0	0.0	P	3.00
2	0.0	F	0.00	0.0	0.0	P	3.00
1	0.0	F	0.00	0.0	0.0	P	3.00
1	0.0	F	0.00	0.0	0.0	P	3.00
2	1.0	F	0.00	0.0	0.0	P	3.00
1	0.0	M	1.00	0.0	0.0	P	3.00
2	0.0	M	1.00	0.0	0.0	B	2.00
2	1.0	M	1.00	0.0	0.0	B	2.00
3	0.0	F	0.00	0.0	0.0	B	2.00
1	1.0	M	1.00	0.0	0.0	B	2.00
2	0.0	F	0.00	0.0	0.0	B	2.00
1	0.0	F	0.00	0.0	0.0	P	3.00
0	0.0	F	0.00	0.0	0.0	P	3.00
2	0.0	F	0.00	0.0	0.0	P	3.00
1	0.0	F	0.00	0.0	0.0	P	3.00
1	0.0	F	0.00	0.0	0.0	P	3.00
1	0.0	F	0.00	0.0	0.0	P	3.00
1	0.0	F	0.00	0.0	0.0	P	3.00
1	0.0	F	0.00	0.0	0.0	P	3.00
2	0.0	F	0.00	0.0	0.0	P	3.00
1	1.0	M	1.00	0.0	0.0	P	3.00
1	0.0	F	0.00	0.0	#NULL!	A	1.00
2	0.0	F	0.00	0.0	0.0	B	2.00
0	1.0	F	0.00	0.0	0.0	B	2.00
3	1.0	F	0.00	0.0	0.0	B	2.00
2	0.0	F	0.00	0.0	0.0	P	3.00
2	0.0	F	0.00	0.0	0.0	P	3.00
1	0.0	M	1.00	0.0	0.0	P	3.00
1	1.0	M	1.00	0.0	0.0	P	3.00
1	0.0	M	1.00	0.0	0.0	P	3.00
2	0.0	F	0.00	0.0	0.0	P	3.00
1	0.0	M	1.00	0.0	0.0	P	3.00
3	0.0	M	1.00	0.0	0.0	P	3.00
0	0.0	F	0.00	0.0	0.0	P	3.00
1	0.0	M	1.00	0.0	1.0	P	3.00
2	0.0	M	1.00	0.0	0.0	P	3.00
3	0.0	F	0.00	0.0	0.0	B	2.00
1	1.0	M	1.00	0.0	0.0	B	2.00
2	0.0	F	0.00	0.0	0.0	B	2.00
1	0.0	F	0.00	0.0	0.0	B	2.00
1	1.0	F	0.00	0.0	0.0	B	2.00
3	0.0	F	0.00	0.0	0.0	B	2.00

1	0.0	M	1.00	0.0	1.0	P	3.00
1	0.0	F	0.00	0.0	0.0	P	3.00
1	0.0	F	0.00	0.0	0.0	P	3.00
1	1.0	F	0.00	0.0	0.0	P	3.00
2	0.0	F	0.00	0.0	0.0	P	3.00
2	0.0	F	0.00	0.0	0.0	P	3.00
1	0.0	F	0.00	0.0	0.0	P	3.00
1	1.0	F	0.00	0.0	0.0	P	3.00
1	1.0	F	0.00	0.0	0.0	P	3.00
1	1.0	M	1.00	0.0	0.0	P	3.00
0	0.0	M	1.00	0.0	0.0	P	3.00
1	0.0	F	0.00	0.0	0.0	A	1.00
0	1.0	M	1.00	0.0	0.0	B	2.00
3	0.0	F	0.00	0.0	0.0	B	2.00
2	0.0	F	0.00	0.0	0.0	B	2.00
2	0.0	F	0.00	0.0	0.0	B	2.00
2	1.0	F	0.00	0.0	0.0	B	2.00
2	1.0	F	0.00	0.0	0.0	B	2.00
1	0.0	F	0.00	0.0	0.0	P	3.00
1	0.0	F	0.00	0.0	0.0	P	3.00
1	0.0	F	0.00	0.0	0.0	P	3.00
1	1.0	F	0.00	0.0	0.0	P	3.00
1	1.0	F	0.00	0.0	0.0	P	3.00
1	0.0	F	0.00	0.0	0.0	P	3.00
2	0.0	M	1.00	0.0	0.0	P	3.00
1	0.0	M	1.00	0.0	0.0	P	3.00
3	0.0	F	0.00	0.0	0.0	B	2.00
1	0.0	F	0.00	0.0	0.0	B	2.00
2	1.0	F	0.00	0.0	0.0	B	2.00
2	1.0	F	0.00	0.0	0.0	P	3.00
2	0.0	F	0.00	0.0	0.0	P	3.00
2	0.0	F	0.00	0.0	0.0	P	3.00
1	0.0	F	0.00	0.0	0.0	P	3.00
2	0.0	F	0.00	0.0	0.0	P	3.00
1	0.0	F	0.00	0.0	0.0	P	3.00
2	0.0	F	0.00	0.0	0.0	P	3.00
1	0.0	M	1.00	0.0	0.0	P	3.00
2	0.0	F	0.00	0.0	0.0	P	3.00
1	1.0	F	0.00	0.0	0.0	P	3.00
1	0.0	F	0.00	0.0	0.0	P	3.00
1	0.0	F	0.00	0.0	0.0	P	3.00
1	1.0	M	1.00	0.0	1.0	P	3.00
2	0.0	F	0.00	1.0	0.0	P	3.00

1	0.0	F	0.00	0.0	0.0	A	1.00
1	1.0	F	0.00	0.0	0.0	A	1.00
3	0.0	F	0.00	0.0	0.0	B	2.00
1	1.0	M	1.00	0.0	0.0	B	2.00
3	1.0	F	0.00	0.0	0.0	B	2.00
2	0.0	F	0.00	0.0	0.0	B	2.00
2	0.0	F	0.00	0.0	0.0	B	2.00
2	1.0	F	0.00	0.0	0.0	B	2.00
3	0.0	F	0.00	0.0	0.0	B	2.00
1	0.0	F	0.00	0.0	0.0	B	2.00
1	0.0	F	0.00	0.0	0.0	B	2.00
2	0.0	M	1.00	0.0	0.0	P	3.00
2	0.0	M	1.00	0.0	0.0	P	3.00
1	0.0	M	1.00	0.0	0.0	P	3.00
1	0.0	M	1.00	0.0	0.0	P	3.00
1	0.0	F	0.00	0.0	0.0	P	3.00
3	1.0	F	0.00	0.0	0.0	P	3.00
2	1.0	M	1.00	0.0	0.0	P	3.00
1	0.0	M	1.00	0.0	0.0	P	3.00
3	0.0	F	0.00	0.0	0.0	B	2.00
2	0.0	F	0.00	0.0	0.0	B	2.00
2	1.0	M	1.00	0.0	0.0	B	2.00
2	0.0	F	0.00	0.0	0.0	P	3.00
1	0.0	M	1.00	0.0	0.0	P	3.00
1	1.0	M	1.00	0.0	0.0	P	3.00
2	1.0	F	0.00	0.0	0.0	P	3.00
1	0.0	M	1.00	0.0	0.0	P	3.00
1	0.0	F	0.00	0.0	0.0	P	3.00
3	0.0	F	0.00	1.0	0.0	B	2.00
3	1.0	F	0.00	0.0	0.0	B	2.00
2	1.0	F	0.00	0.0	0.0	B	2.00
2	0.0	F	0.00	0.0	0.0	B	2.00
2	1.0	F	0.00	0.0	0.0	B	2.00
2	1.0	F	0.00	0.0	0.0	B	2.00
1	0.0	F	0.00	0.0	0.0	B	2.00
2	0.0	F	0.00	0.0	0.0	P	3.00
2	0.0	F	0.00	0.0	0.0	P	3.00
#NULL!	0.0	M	1.00	0.0	0.0	P	3.00
3	1.0	F	0.00	0.0	1.0	B	2.00
2	0.0	F	0.00	0.0	0.0	B	2.00
1	0.0	F	0.00	0.0	0.0	B	2.00
2	0.0	F	0.00	0.0	0.0	P	3.00
1	1.0	F	0.00	0.0	1.0	P	3.00

1	1.0 F	0.00	0.0	0.0 P	3.00
2	1.0 F	0.00	0.0	0.0 P	3.00
2	0.0 F	0.00	0.0	0.0 P	3.00
2	0.0 F	0.00	0.0	0.0 P	3.00
2	0.0 M	1.00	1.0	0.0 B	2.00
3	0.0 F	0.00	0.0	0.0 B	2.00
2	1.0 F	0.00	0.0	0.0 B	2.00
2	0.0 F	0.00	0.0	0.0 B	2.00
1	1.0 F	0.00	0.0	0.0 B	2.00
1	1.0 F	0.00	0.0	0.0 P	3.00
2	0.0 M	1.00	0.0	0.0 P	3.00
2	0.0 F	0.00	0.0	1.0 P	3.00
2	0.0 F	0.00	0.0	0.0 P	3.00
2	1.0 M	1.00	0.0	0.0 P	3.00
2	0.0 F	0.00	0.0	0.0 B	2.00
3	0.0 F	0.00	0.0	0.0 B	2.00
2	0.0 F	0.00	0.0	0.0 P	3.00
2	0.0 M	1.00	0.0	0.0 B	2.00
2	0.0 F	0.00	0.0	0.0 B	2.00
2	0.0 F	0.00	0.0	0.0 B	2.00
3	0.0 F	0.00	0.0	0.0 P	3.00
1	0.0 F	0.00	0.0	0.0 P	3.00
1	0.0 F	0.00	0.0	0.0 P	3.00
1	0.0 M	1.00	0.0	0.0 P	3.00
2	0.0 M	1.00	0.0	0.0 P	3.00
1	0.0 F	0.00	0.0	0.0 P	3.00
3	0.0 M	1.00	0.0	0.0 P	3.00
2	1.0 F	0.00	0.0	0.0 P	3.00
2	1.0 F	0.00	0.0	0.0 P	3.00
1	0.0 F	0.00	0.0	0.0 P	3.00
2	0.0 F	0.00	0.0	0.0 B	2.00

Bilateral	Age	Ht	MAC_site	MAC_max1	SBP	DBP	PP
0.00	71.0	160.0	P	16.5	152.0	85.0	67.0
1.00	50.0	167.0	B	14.2	166.0	93.0	73.0
0.00	54.0	174.0	P	5.1	122.0	72.0	50.0
1.00	56.0	172.0	B	12.0	154.0	98.0	56.0
0.00	56.0	173.0	P	3.0	170.0	103.0	67.0
0.00	57.0	166.0	P	3.9	135.0	69.0	66.0
0.00	57.0	158.0	P	4.8	122.0	68.0	54.0
0.00	58.0	173.0	P	9.2	152.0	76.0	76.0
0.00	58.0	164.0	P	4.2	133.0	66.0	67.0
0.00	60.0	174.0	P	3.8	160.0	68.0	92.0
1.00	62.0	163.0	B	5.9	164.0	77.0	87.0
0.00	63.0	158.0	P	15.2	137.0	81.0	56.0
0.00	63.0	144.0	P	7.2	150.0	80.0	70.0
0.00	64.0	146.0	P	4.3	144.0	64.0	80.0
0.00	66.0	158.0	P	10.2	174.0	121.0	53.0
0.00	67.0	161.0	P	4.3	97.0	67.0	30.0
0.00	67.0	183.0	P	6.4	123.0	76.0	47.0
0.00	68.0	153.0	P	4.8	164.0	81.0	83.0
0.00	68.0	174.0	P	3.8	126.0	78.0	48.0
0.00	68.0	167.0	P	5.6	100.0	61.0	39.0
0.00	69.0	160.0	P	17.2	117.0	80.0	37.0
0.00	69.0	168.0	P	2.1	173.0	90.0	83.0
0.00	69.0	168.0	P	3.4	108.0	62.0	46.0
0.00	70.0	154.0	P	7.6	135.0	59.0	76.0
0.00	70.0	168.0	P	3.8	114.0	72.0	42.0
1.00	72.0	160.0	B	6.4	114.0	70.0	44.0
0.00	72.0	155.0	P	10.4	132.0	70.0	62.0
1.00	73.0	167.0	B	10.4	151.0	64.0	87.0
1.00	73.0	160.0	B	4.4	160.0	44.0	116.0
1.00	73.0	170.0	B	18.0	160.0	75.0	85.0
0.00	73.0	147.0	P	4.0	88.0	59.0	29.0
0.00	73.0	140.0	P	3.2	119.0	74.0	45.0
0.00	74.0	145.0	P	1.8	135.0	80.0	55.0
0.00	74.0	153.0	P	5.0	166.0	79.0	87.0
0.00	74.0	159.0	P	4.1	137.0	80.0	57.0
1.00	75.0	147.0	B	10.8	115.0	65.0	50.0
1.00	75.0	158.0	B	4.8	147.0	76.0	71.0
1.00	75.0	154.0	B	5.4	120.0	80.0	40.0
0.00	75.0	170.0	P	2.1	120.0	73.0	47.0
1.00	76.0	162.0	B	7.1	169.0	92.0	77.0
1.00	76.0	150.0	B	3.5	144.0	78.0	66.0
0.00	76.0	154.0	P	3.0	165.0	50.0	115.0

0.00	76.0	156.0	P	2.5	148.0	79.0	69.0
0.00	76.0	162.0	P	9.0	179.0	96.0	83.0
0.00	77.0	178.0	P	4.1	141.0	85.0	56.0
0.00	77.0	165.0	P	4.3	124.0	57.0	67.0
0.00	77.0	156.0	P	3.5	97.0	56.0	41.0
0.00	77.0	177.0	P	4.2	134.0	79.0	55.0
1.00	78.0	150.0	B	4.5	110.0	60.0	50.0
0.00	78.0	170.0	P	11.5	152.0	78.0	74.0
0.00	78.0	169.0	P	2.4	131.0	49.0	82.0
0.00	78.0	150.0	P	4.7	125.0	76.0	49.0
0.00	78.0	170.0	P	6.0	126.0	63.0	63.0
1.00	79.0	150.0	B	7.7	#NULL!	#NULL!	0.0
1.00	79.0	150.0	B	6.9	141.0	80.0	61.0
0.00	79.0	168.0	P	3.7	144.0	82.0	62.0
0.00	79.0	163.0	P	3.9	152.0	73.0	79.0
0.00	79.0	155.0	P	6.9	121.0	65.0	56.0
1.00	80.0	157.0	B	5.4	120.0	80.0	40.0
1.00	80.0	166.0	B	10.4	140.0	80.0	60.0
1.00	80.0	150.0	B	6.0	97.0	32.0	65.0
0.00	81.0	164.0	P	3.7	148.0	86.0	62.0
1.00	82.0	156.0	B	3.2	168.0	78.0	90.0
1.00	82.0	155.0	B	4.7	154.0	97.0	57.0
1.00	83.0	148.0	B	13.7	166.0	99.0	67.0
1.00	83.0	168.0	B	1.9	127.0	48.0	79.0
1.00	83.0	157.0	B	4.7	138.0	78.0	60.0
1.00	84.0	160.0	B	5.3	135.0	72.0	63.0
1.00	84.0	150.0	B	9.7	130.0	60.0	70.0
1.00	84.0	163.0	B	4.2	147.0	58.0	89.0
1.00	85.0	149.0	B	6.8	133.0	69.0	64.0
0.00	85.0	167.0	P	4.5	148.0	76.0	72.0
0.00	86.0	152.0	P	5.1	169.0	109.0	60.0
0.00	86.0	157.0	P	3.3	150.0	73.0	77.0
0.00	86.0	164.0	P	5.7	139.0	67.0	72.0
1.00	87.0	164.0	B	3.8	164.0	98.0	66.0
0.00	87.0	168.0	P	3.7	160.0	46.0	114.0
0.00	87.0	170.0	P	3.9	130.0	84.0	46.0
1.00	88.0	165.0	B	5.9	116.0	45.0	71.0
0.00	88.0	147.0	P	5.4	124.0	59.0	65.0
0.00	88.0	165.0	P	4.5	126.0	60.0	66.0
0.00	88.0	165.0	P	5.4	141.0	64.0	77.0
0.00	89.0	163.0	P	3.4	102.0	51.0	51.0
0.00	89.0	174.0	P	2.8	155.0	78.0	77.0
0.00	90.0	145.0	P	10.3	143.0	67.0	76.0

1.00	92.0	140.0 B	9.5	154.0	78.0	76.0
0.00	93.0	140.0 P	3.4	159.0	74.0	85.0
1.00	95.0	150.0 B	2.9	104.0	67.0	37.0
0.00	34.0	152.0 P	2.3	170.0	70.0	100.0
1.00	38.0	154.0 B	5.3	138.0	71.0	67.0
1.00	38.0	148.0 B	11.8	141.0	87.0	54.0
0.00	39.0	159.0 P	8.3	141.0	101.0	40.0
0.00	39.0	163.0 P	2.7	128.0	107.0	21.0
1.00	44.0	161.0 P	10.3	117.0	72.0	45.0
1.00	45.0	157.0 B	4.2	127.0	75.0	52.0
0.00	45.0	169.0 P	3.9	120.0	83.0	37.0
0.00	47.0	180.0 P	6.1	196.0	110.0	86.0
1.00	48.0	167.0 B	5.8	99.0	81.0	18.0
0.00	49.0	168.0 P	3.4	167.0	85.0	82.0
0.00	49.0	155.0 P	2.0	109.0	72.0	37.0
1.00	50.0	168.0 B	10.4	154.0	97.0	57.0
1.00	51.0	164.0 B	6.2	135.0	79.0	56.0
1.00	51.0	157.0 B	3.6	129.0	79.0	50.0
0.00	52.0	173.0 P	2.0	90.0	61.0	29.0
0.00	52.0	150.0 P	4.6	112.0	88.0	24.0
1.00	53.0	177.0 B	8.8	130.0	70.0	60.0
0.00	53.0	172.0 P	11.6	136.0	81.0	55.0
0.00	53.0	173.0 P	2.3	119.0	66.0	53.0
0.00	54.0	154.0 A	2.7	135.0	76.0	59.0
1.00	54.0	160.0 B	4.3	167.0	87.0	80.0
0.00	54.0	177.0 P	3.8	0.0	0.0	0.0
0.00	54.0	170.0 P	3.1	164.0	91.0	73.0
1.00	55.0	158.0 B	10.4	153.0	84.0	69.0
0.00	55.0	150.0 P	2.8	120.0	63.0	57.0
0.00	55.0	154.0 P	3.1	141.0	57.0	84.0
1.00	56.0	153.0 B	10.2	108.0	54.0	54.0
1.00	56.0	155.0 B	13.9	122.0	68.0	54.0
0.00	56.0	167.0 P	3.9	154.0	93.0	61.0
1.00	57.0	160.0 B	20.2	165.0	92.0	73.0
1.00	57.0	157.0 B	12.8	110.0	70.0	40.0
1.00	57.0	172.0 B	13.1	128.0	60.0	68.0
1.00	57.0	177.0 B	7.8	135.0	89.0	46.0
0.00	57.0	170.0 P	15.8	145.0	67.0	78.0
1.00	58.0	152.0 B	10.5	100.0	70.0	30.0
1.00	58.0	158.0 B	6.8	140.0	74.0	66.0
0.00	58.0	172.0 P	2.6	139.0	89.0	50.0
0.00	58.0	161.0 P	11.1	137.0	59.0	78.0
0.00	58.0	160.0 P	3.0	120.0	80.0	40.0

0.00	58.0	166.0 P	15.4	160.0	72.0	88.0
1.00	59.0	150.0 B	8.8	153.0	72.0	81.0
1.00	59.0	173.0 B	13.8	170.0	65.0	105.0
1.00	59.0	175.0 B	7.2	100.0	70.0	30.0
0.00	59.0	153.0 P	3.7	119.0	75.0	44.0
0.00	59.0	148.0 P	2.5	159.0	84.0	75.0
1.00	60.0	153.0 B	18.2	199.0	99.0	100.0
0.00	60.0	146.0 P	6.0	110.0	70.0	40.0
1.00	61.0	170.0 B	5.7	98.0	68.0	30.0
0.00	61.0	172.0 P	3.2	119.0	79.0	40.0
0.00	61.0	170.0 P	16.6	108.0	80.0	28.0
0.00	62.0	167.0 P	3.5	152.0	80.0	72.0
0.00	62.0	161.0 P	4.5	160.0	92.0	68.0
0.00	63.0	158.0 P	4.9	144.0	85.0	59.0
0.00	63.0	150.0 P	3.3	113.0	52.0	61.0
0.00	63.0	167.0 P	3.7	130.0	74.0	56.0
0.00	63.0	160.0 P	4.0	103.0	60.0	43.0
0.00	63.0	160.0 P	8.5	106.0	66.0	40.0
1.00	64.0	171.0 B	5.2	147.0	80.0	67.0
0.00	64.0	160.0 P	29.8	125.0	80.0	45.0
0.00	64.0	160.0 P	8.3	135.0	70.0	65.0
0.00	64.0	160.0 P	2.0	125.0	67.0	58.0
0.00	64.0	166.0 P	2.3	145.0	83.0	62.0
1.00	65.0	162.0 B	10.2	168.0	53.0	115.0
1.00	65.0	170.0 B	5.3	147.0	64.0	83.0
0.00	65.0	170.0 P	2.3	117.0	72.0	45.0
0.00	65.0	162.0 P	4.6	115.0	71.0	44.0
0.00	65.0	140.0 P	2.5	109.0	69.0	40.0
0.00	65.0	170.0 P	3.3	116.0	81.0	35.0
1.00	66.0	146.0 B	5.7	140.0	72.0	68.0
1.00	66.0	154.0 B	3.3	176.0	65.0	111.0
0.00	66.0	170.0 P	15.9	110.0	50.0	60.0
0.00	66.0	170.0 P	2.5	100.0	66.0	34.0
0.00	66.0	170.0 P	3.7	146.0	87.0	59.0
0.00	66.0	154.0 P	4.2	146.0	96.0	50.0
0.00	66.0	159.0 P	7.6	163.0	76.0	87.0
0.00	66.0	146.0 P	5.5	138.0	84.0	54.0
0.00	66.0	169.0 P	5.2	116.0	68.0	48.0
0.00	66.0	170.0 P	14.2	107.0	70.0	37.0
0.00	67.0	169.0 P	8.8	144.0	73.0	71.0
0.00	67.0	155.0 P	15.6	176.0	83.0	93.0
0.00	67.0	158.0 P	4.1	118.0	82.0	36.0
0.00	67.0	151.0 P	4.1	147.0	82.0	65.0

1.00	68.0	171.0	B	4.2	130.0	72.0	58.0
1.00	68.0	158.0	B	2.9	105.0	66.0	39.0
1.00	68.0	170.0	B	5.8	125.0	39.0	86.0
0.00	68.0	164.0	P	4.1	112.0	72.0	40.0
0.00	68.0	155.0	P	13.4	169.0	93.0	76.0
0.00	68.0	149.0	P	2.8	103.0	66.0	37.0
0.00	68.0	163.0	P	2.7	161.0	96.0	65.0
0.00	68.0	167.0	P	6.4	152.0	91.0	61.0
1.00	69.0	152.0	B	17.0	112.0	67.0	45.0
1.00	69.0	168.0	B	13.7	153.0	82.0	71.0
0.00	69.0	163.0	P	5.8	145.0	79.0	66.0
0.00	69.0	149.0	P	3.6	156.0	85.0	71.0
0.00	69.0	167.0	P	2.1	142.0	53.0	89.0
0.00	69.0	152.0	P	3.6	157.0	81.0	76.0
0.00	69.0	164.0	P	3.6	145.0	84.0	61.0
1.00	69.0	152.0	B	5.9	183.0	80.0	103.0
1.00	70.0	150.0	B	10.8	160.0	73.0	87.0
1.00	70.0	167.0	B	5.1	109.0	65.0	44.0
1.00	70.0	161.0	B	16.8	143.0	80.0	63.0
0.00	70.0	152.0	P	3.9	130.0	76.0	54.0
0.00	70.0	165.0	P	2.6	139.0	76.0	63.0
1.00	71.0	155.0	B	4.3	145.0	77.0	68.0
0.00	71.0	161.0	P	2.5	131.0	61.0	70.0
0.00	71.0	160.0	P	2.6	148.0	78.0	70.0
0.00	72.0	156.0	A	4.9	165.0	79.0	86.0
1.00	72.0	145.0	B	6.6	120.0	80.0	40.0
1.00	72.0	163.0	B	4.8	133.0	71.0	62.0
1.00	72.0	152.0	B	11.4	128.0	81.0	47.0
1.00	72.0	151.0	B	4.4	131.0	67.0	64.0
0.00	72.0	158.0	P	3.8	121.0	57.0	64.0
0.00	72.0	165.0	P	5.4	79.0	51.0	28.0
0.00	72.0	162.0	P	2.9	145.0	74.0	71.0
0.00	72.0	161.0	P	5.1	113.0	90.0	23.0
0.00	72.0	167.0	P	5.1	129.0	46.0	83.0
0.00	72.0	152.0	P	7.7	178.0	93.0	85.0
0.00	72.0	153.0	P	13.6	137.0	68.0	69.0
0.00	72.0	162.0	P	5.3	152.0	87.0	54.0
0.00	72.0	162.0	P	4.1	158.0	87.0	71.0
0.00	72.0	163.0	P	3.5	120.0	76.0	44.0
0.00	72.0	155.0	P	5.5	107.0	54.0	53.0
0.00	72.0	155.0	P	3.3	147.0	106.0	41.0
0.00	72.0	170.0	P	2.0	148.0	87.0	61.0
1.00	73.0	165.0	B	4.7	130.0	88.0	42.0

1.00	73.0	162.0	B	8.8	138.0	82.0	56.0
1.00	73.0	164.0	B	15.3	149.0	88.0	61.0
1.00	73.0	152.0	B	10.8	140.0	90.0	50.0
1.00	73.0	150.0	B	12.4	162.0	74.0	88.0
1.00	73.0	153.0	B	3.8	118.0	60.0	58.0
1.00	73.0	147.0	B	2.9	135.0	60.0	75.0
1.00	73.0	175.0	B	3.4	112.0	82.0	30.0
0.00	73.0	174.0	P	5.4	124.0	81.0	43.0
0.00	73.0	160.0	P	18.6	135.0	72.0	63.0
0.00	73.0	152.0	P	5.0	145.0	81.0	64.0
0.00	73.0	168.0	P	6.7	87.0	52.0	35.0
0.00	73.0	165.0	P	7.6	160.0	71.0	89.0
0.00	73.0	159.0	P	3.5	127.0	91.0	36.0
1.00	73.0	150.0	B	11.6	180.0	80.0	100.0
1.00	74.0	152.0	B	8.6	158.0	72.0	86.0
1.00	74.0	144.0	B	4.9	130.0	70.0	60.0
1.00	74.0	160.0	B	6.1	100.0	71.0	29.0
1.00	74.0	154.0	B	4.7	104.0	69.0	35.0
1.00	74.0	163.0	B	1.9	105.0	55.0	50.0
1.00	74.0	144.0	P	10.4	133.0	64.0	69.0
1.00	74.0	155.0	B	12.0	121.0	67.0	54.0
1.00	74.0	168.0	B	6.4	110.0	63.0	47.0
1.00	74.0	164.0	B	7.5	134.0	65.0	69.0
0.00	74.0	160.0	p	4.6	153.0	78.0	75.0
0.00	74.0	159.0	P	5.5	144.0	71.0	73.0
0.00	74.0	160.0	P	4.7	130.0	70.0	60.0
0.00	74.0	165.0	P	3.6	151.0	77.0	74.0
0.00	74.0	173.0	P	5.2	135.0	83.0	52.0
0.00	74.0	154.0	P	2.5	120.0	70.0	50.0
0.00	74.0	159.0	P	4.2	136.0	82.0	54.0
0.00	74.0	158.0	P	5.8	108.0	75.0	33.0
0.00	74.0	160.0	P	3.7	129.0	77.0	52.0
0.00	74.0	154.0	P	3.6	115.0	53.0	62.0
0.00	74.0	160.0	P	6.2	154.0	77.0	77.0
0.00	74.0	158.0	P	5.2	187.0	97.0	90.0
0.00	74.0	150.0	P	5.6	179.0	99.0	80.0
0.00	74.0	154.0	P	5.5	105.0	60.0	45.0
0.00	74.0	156.0	P	9.5	130.0	81.0	49.0
0.00	74.0	152.0	P	3.1	129.0	71.0	58.0
0.00	74.0	157.0	P	4.7	113.0	65.0	48.0
0.00	74.0	150.0	P	6.8	147.0	74.0	73.0
1.00	74.0	148.0	P	7.3	122.0	70.0	52.0
0.00	75.0	149.0	A	3.0	132.0	50.0	82.0

1.00	75.0	157.0	B	19.9	146.0	80.0	66.0
1.00	75.0	152.0	B	4.8	166.0	53.0	113.0
1.00	75.0	155.0	B	3.7	182.0	101.0	81.0
1.00	75.0	158.0	B	9.4	142.0	79.0	63.0
1.00	75.0	145.0	B	5.4	127.0	70.0	57.0
1.00	75.0	144.0	B	4.6	134.0	87.0	47.0
1.00	75.0	138.0	B	3.0	138.0	74.0	64.0
0.00	75.0	137.0	P	9.7	128.0	69.0	59.0
0.00	75.0	165.0	P	2.8	149.0	60.0	89.0
0.00	75.0	159.0	P	3.8	133.0	73.0	60.0
0.00	75.0	155.0	P	12.1	130.0	74.0	56.0
0.00	75.0	160.0	P	6.0	172.0	83.0	89.0
0.00	75.0	150.0	P	7.7	149.0	70.0	79.0
0.00	75.0	150.0	P	4.0	137.0	72.0	65.0
0.00	75.0	153.0	P	4.0	146.0	77.0	69.0
0.00	75.0	146.0	P	5.7	150.0	90.0	60.0
0.00	75.0	146.0	P	5.9	107.0	58.0	49.0
0.00	75.0	160.0	P	5.6	132.0	95.0	37.0
0.00	75.0	162.0	P	4.4	132.0	68.0	64.0
0.00	75.0	160.0	P	4.5	133.0	71.0	62.0
0.00	75.0	159.0	P	11.8	153.0	90.0	63.0
1.00	76.0	153.0	B	13.5	155.0	80.0	75.0
1.00	76.0	149.0	B	9.5	133.0	65.0	68.0
1.00	76.0	152.0	B	2.9	105.0	66.0	39.0
1.00	76.0	154.0	B	5.6	150.0	93.0	57.0
1.00	76.0	175.0	B	9.0	140.0	90.0	50.0
0.00	76.0	168.0	P	2.1	130.0	70.0	60.0
0.00	76.0	166.0	P	9.6	139.0	65.0	74.0
0.00	76.0	160.0	P	6.1	139.0	70.0	69.0
0.00	76.0	169.0	P	11.2	149.0	90.0	59.0
0.00	76.0	148.0	P	4.2	114.0	61.0	53.0
0.00	76.0	161.0	P	12.1	154.0	75.0	79.0
0.00	76.0	156.0	P	4.2	146.0	85.0	61.0
0.00	76.0	155.0	P	16.4	139.0	66.0	73.0
0.00	76.0	163.0	P	2.0	115.0	77.0	38.0
1.00	77.0	146.0	B	5.6	118.0	58.0	60.0
1.00	77.0	153.0	B	14.7	171.0	88.0	83.0
1.00	77.0	165.0	B	14.3	147.0	89.0	58.0
1.00	77.0	171.0	B	5.6	110.0	60.0	50.0
1.00	77.0	163.0	B	16.2	128.0	81.0	47.0
0.00	77.0	155.0	P	16.0	120.0	50.0	70.0
0.00	77.0	150.0	P	2.2	105.0	60.0	45.0
0.00	77.0	175.0	P	2.7	114.0	87.0	27.0

0.00	77.0	167.0	P	2.9	211.0	88.0	123.0
0.00	77.0	157.0	P	4.3	134.0	64.0	70.0
0.00	77.0	151.0	P	4.6	148.0	76.0	72.0
0.00	77.0	154.0	P	2.5	136.0	80.0	56.0
0.00	77.0	155.0	P	3.4	138.0	68.0	70.0
0.00	77.0	165.0	P	5.4	106.0	68.0	38.0
0.00	77.0	164.0	P	4.6	197.0	95.0	102.0
1.00	78.0	169.0	P	5.3	103.0	60.0	43.0
1.00	78.0	173.0	B	4.5	117.0	69.0	48.0
1.00	78.0	154.0	B	7.5	125.0	77.0	48.0
1.00	78.0	164.0	B	5.7	141.0	76.0	65.0
1.00	78.0	147.0	B	4.8	126.0	50.0	76.0
0.00	78.0	160.0	P	3.8	157.0	88.0	69.0
0.00	78.0	145.0	P	11.4	168.0	70.0	98.0
0.00	78.0	147.0	P	8.5	175.0	105.0	70.0
0.00	78.0	150.0	P	5.7	122.0	73.0	49.0
0.00	78.0	158.0	P	2.9	154.0	78.0	76.0
0.00	78.0	155.0	P	2.1	102.0	77.0	25.0
0.00	78.0	158.0	P	2.4	92.0	62.0	30.0
0.00	78.0	151.0	P	3.8	126.0	69.0	57.0
0.00	78.0	158.0	P	10.2	118.0	43.0	75.0
0.00	78.0	163.0	P	3.3	110.0	62.0	48.0
0.00	79.0	158.0	A	2.9	114.0	67.0	47.0
1.00	79.0	160.0	B	5.7	134.0	64.0	70.0
1.00	79.0	156.0	B	7.8	126.0	74.0	52.0
1.00	79.0	156.0	B	10.5	145.0	70.0	75.0
0.00	79.0	151.0	P	6.5	156.0	81.0	75.0
0.00	79.0	142.0	P	5.6	146.0	95.0	51.0
0.00	79.0	179.0	P	2.3	136.0	70.0	66.0
0.00	79.0	158.0	P	2.6	137.0	103.0	34.0
0.00	79.0	176.0	P	3.8	185.0	89.0	96.0
0.00	79.0	147.0	P	5.0	162.0	88.0	74.0
0.00	79.0	166.0	P	3.7	110.0	56.0	54.0
0.00	79.0	163.0	P	10.5	127.0	66.0	61.0
0.00	79.0	149.0	P	10.1	149.0	84.0	65.0
0.00	79.0	155.0	P	3.5	154.0	87.0	67.0
0.00	79.0	185.0	P	3.9	145.0	71.0	74.0
1.00	80.0	150.0	P	16.1	126.0	72.0	54.0
1.00	80.0	162.0	B	2.0	129.0	69.0	60.0
1.00	80.0	138.0	B	5.6	125.0	60.0	65.0
1.00	80.0	144.0	B	3.9	138.0	71.0	67.0
1.00	80.0	143.0	B	3.0	109.0	73.0	36.0
1.00	80.0	155.0	P	14.6	147.0	75.0	72.0

0.00	80.0	160.0 P	3.2	140.0	80.0	60.0
0.00	80.0	160.0 P	3.8	140.0	70.0	70.0
0.00	80.0	167.0 P	3.0	115.0	50.0	65.0
0.00	80.0	154.0 P	3.8	152.0	79.0	73.0
0.00	80.0	157.0 P	6.7	113.0	66.0	47.0
0.00	80.0	143.0 P	5.1	135.0	70.0	65.0
0.00	80.0	159.0 P	2.5	146.0	69.0	77.0
0.00	80.0	162.0 P	3.6	111.0	53.0	58.0
0.00	80.0	161.0 P	3.4	146.0	65.0	81.0
0.00	80.0	153.0 P	2.7	155.0	94.0	61.0
0.00	80.0	165.0 P	15.7	172.0	125.0	47.0
0.00	81.0	152.0 A	3.4	150.0	77.0	73.0
1.00	81.0	166.0 B	12.8	104.0	75.0	29.0
1.00	81.0	150.0 B	20.5	197.0	38.0	159.0
1.00	81.0	148.0 B	4.6	164.0	63.0	101.0
1.00	81.0	150.0 B	4.3	133.0	84.0	49.0
1.00	81.0	150.0 B	5.7	144.0	59.0	85.0
1.00	81.0	151.0 B	3.1	154.0	73.0	81.0
0.00	81.0	164.0 P	3.6	128.0	88.0	40.0
0.00	81.0	158.0 P	2.0	144.0	64.0	80.0
0.00	81.0	153.0 P	2.8	108.0	59.0	49.0
0.00	81.0	150.0 P	2.0	149.0	52.0	97.0
0.00	81.0	162.0 P	4.0	159.0	102.0	57.0
0.00	81.0	158.0 P	3.0	131.0	78.0	53.0
0.00	81.0	157.0 P	5.2	115.0	65.0	50.0
0.00	81.0	162.0 P	4.9	103.0	71.0	32.0
1.00	82.0	147.0 B	9.1	131.0	71.0	60.0
1.00	82.0	152.0 B	4.4	116.0	82.0	34.0
1.00	82.0	146.0 B	5.6	148.0	71.0	77.0
0.00	82.0	153.0 P	4.3	120.0	61.0	59.0
0.00	82.0	156.0 P	4.4	122.0	68.0	54.0
0.00	82.0	157.0 P	3.9	147.0	84.0	63.0
0.00	82.0	140.0 P	3.9	146.0	67.0	79.0
0.00	82.0	146.0 P	6.2	200.0	97.0	103.0
0.00	82.0	150.0 P	6.5	141.0	49.0	92.0
0.00	82.0	157.0 P	4.6	169.0	74.0	95.0
0.00	82.0	164.0 P	4.0	122.0	46.0	76.0
0.00	82.0	150.0 P	9.1	124.0	71.0	53.0
0.00	82.0	141.0 P	3.2	99.0	58.0	41.0
0.00	82.0	152.0 P	3.6	142.0	78.0	64.0
0.00	82.0	140.0 P	3.6	102.0	52.0	50.0
0.00	82.0	166.0 P	3.2	127.0	63.0	64.0
0.00	82.0	150.0 P	13.1	159.0	84.0	75.0

0.00	83.0	150.0 A	8.1	184.0	65.0	119.0
0.00	83.0	153.0 A	2.1	131.0	93.0	38.0
1.00	83.0	164.0 B	19.1	150.0	80.0	70.0
1.00	83.0	170.0 B	7.2	133.0	68.0	65.0
1.00	83.0	150.0 B	6.5	129.0	82.0	47.0
1.00	83.0	153.0 B	3.0	148.0	78.0	70.0
1.00	83.0	149.0 B	15.5	151.0	62.0	89.0
1.00	83.0	149.0 B	3.2	161.0	65.0	96.0
1.00	83.0	150.0 B	16.2	122.0	63.0	59.0
1.00	83.0	156.0 B	2.9	173.0	93.0	80.0
1.00	83.0	150.0 B	2.9	156.0	106.0	50.0
0.00	83.0	169.0 P	3.5	152.0	66.0	86.0
0.00	83.0	170.0 P	3.2	153.0	54.0	99.0
0.00	83.0	162.0 P	2.9	126.0	70.0	56.0
0.00	83.0	159.0 P	4.1	113.0	75.0	38.0
0.00	83.0	153.0 P	3.8	162.0	70.0	92.0
0.00	83.0	156.0 P	15.7	154.0	74.0	80.0
0.00	83.0	169.0 P	7.3	138.0	82.0	56.0
0.00	83.0	162.0 P	6.3	148.0	86.0	62.0
1.00	84.0	145.0 B	6.2	142.0	87.0	55.0
1.00	84.0	148.0 B	5.7	110.0	72.0	38.0
1.00	84.0	155.0 B	5.3	146.0	76.0	70.0
0.00	84.0	158.0 P	8.7	140.0	65.0	75.0
0.00	84.0	172.0 P	3.5	126.0	66.0	60.0
0.00	84.0	163.0 P	2.9	136.0	65.0	71.0
0.00	84.0	160.0 P	5.6	151.0	79.0	72.0
0.00	84.0	165.0 P	4.7	133.0	84.0	49.0
0.00	84.0	160.0 P	2.4	173.0	72.0	101.0
1.00	84.0	163.0 B	10.4	93.0	43.0	50.0
1.00	85.0	148.0 B	11.1	137.0	66.0	71.0
1.00	85.0	145.0 B	2.6	96.0	53.0	43.0
1.00	85.0	158.0 B	3.8	167.0	79.0	88.0
1.00	85.0	155.0 B	4.9	123.0	74.0	49.0
1.00	85.0	160.0 B	4.7	108.0	47.0	61.0
1.00	85.0	158.0 B	3.9	162.0	94.0	68.0
0.00	85.0	151.0 P	13.7	160.0	76.0	84.0
0.00	85.0	160.0 P	5.4	154.0	72.0	82.0
0.00	85.0	171.0 P	3.3	148.0	81.0	67.0
1.00	86.0	160.0 B	11.8	109.0	66.0	43.0
1.00	86.0	160.0 B	7.1	130.0	80.0	50.0
1.00	86.0	151.0 B	3.3	122.0	53.0	69.0
0.00	86.0	154.0 P	14.3	128.0	54.0	74.0
0.00	86.0	145.0 P	4.4	134.0	78.0	56.0

0.00	86.0	158.0	P	3.8	147.0	80.0	67.0
0.00	86.0	157.0	P	7.5	142.0	78.0	64.0
0.00	86.0	163.0	P	8.3	150.0	78.0	72.0
0.00	86.0	152.0	P	5.6	116.0	35.0	81.0
1.00	86.0	163.0	B	8.3	120.0	60.0	60.0
1.00	87.0	138.0	B	5.2	161.0	72.0	89.0
1.00	87.0	149.0	B	2.8	115.0	66.0	49.0
1.00	87.0	153.0	B	7.6	110.0	59.0	51.0
1.00	87.0	150.0	B	1.8	123.0	82.0	41.0
0.00	87.0	150.0	P	3.4	118.0	57.0	61.0
0.00	88.0	160.0	P	3.8	118.0	68.0	50.0
0.00	88.0	151.0	P	6.1	138.0	66.0	72.0
0.00	88.0	149.0	P	3.8	150.0	89.0	61.0
0.00	88.0	171.0	P	4.1	136.0	64.0	72.0
1.00	89.0	142.0	B	3.8	168.0	88.0	80.0
1.00	90.0	150.0	B	5.8	120.0	70.0	50.0
0.00	90.0	148.0	P	3.8	115.0	60.0	55.0
1.00	91.0	162.0	B	8.2	120.0	80.0	40.0
1.00	91.0	153.0	B	7.9	140.0	76.0	64.0
1.00	91.0	153.0	B	3.3	101.0	48.0	53.0
0.00	91.0	163.0	P	14.2	115.0	67.0	48.0
0.00	91.0	155.0	P	3.3	109.0	72.0	37.0
0.00	91.0	151.0	P	1.8	123.0	72.0	51.0
0.00	92.0	172.0	P	3.5	120.0	70.0	50.0
0.00	93.0	178.0	P	6.3	130.0	73.0	57.0
0.00	93.0	133.0	P	3.3	140.0	70.0	70.0
0.00	93.0	164.0	P	11.5	122.0	59.0	63.0
0.00	95.0	162.0	P	4.6	105.0	51.0	54.0
0.00	95.0	145.0	P	5.6	144.0	76.0	68.0
0.00	96.0	150.0	P	5.2	118.0	71.0	47.0
1.00	97.0	158.0	B	3.7	158.0	64.0	94.0

LVEDD	LVESD	EF	LAVI	E	Eem	RVSP	Sm_RV
43.0	28.0	67	23.0	0.69	16.4	27.0	
50.0	34.0	#NULL!	64.1	1.24	31.0	67.0	4
58.0	41.0	60	50.2	1.18	20.0	#NULL!	8
46.0	32.0	61	32.6	1.12	17.1	29.0	8.4
60.0	47.0	43	124.0	0.86	14.0	62.0	4
59.0	47.0	46	105.6	1.35	34.0	17.0	4
46.0	30.0	67	29.7	0.46	21.0	15.0	5
59.0	46.0	#NULL!	60.1	1.54	39.0	45.0	5
44.0	22.0	85	#NULL!	1.25	31.3	32.0	7.7
47.0	32.0	63	46.1	1.37	25.0	42.0	4.9
53.0	34.0	68	#NULL!	1.37	34.0	29.0	5
45.0	24.0	79	43.4	0.90	23.0	31.0	6
37.0	26.0	60	39.5	0.67	22.3	17.6	6.8
53.0	33.0	71	65.5	1.38	35.0	37.0	7
45.0	24.0	81	#NULL!	1.72	44.0	60.0	6
67.0	65.0	10	#NULL!	1.12	38.0	68.0	2
61.0	45.0	50	84.5	1.11	16.0	32.0	6
48.0	32.0	65	#NULL!	1.39	34.7	32.0	5
67.0	50.0	34	62.9	1.00	25.0	28.0	3
52.0	32.0	72	39.5	1.46	16.2	49.0	9
46.0	29.0	70	33.1	0.90	23.0	31.0	9
61.0	44.0	52	50.2	0.55	18.0	27.0	6
52.0	37.0	59	54.6	0.67	13.4	36.0	6
44.0	31.0	60	54.9	0.95	11.6	34.0	6.1
56.0	38.0	63	#NULL!	1.02	17.0	41.0	6
35.0	22.0	70	#NULL!	1.18	47.0	24.0	5.4
44.0	29.0	66	34.7	0.95	19.3	22.0	5.5
52.0	35.0	64	45.7	0.85	17.0	30.0	7
53.0	32.0	73	32.4	0.78	13.0	#NULL!	10
52.0	37.0	59	68.5	1.09	27.0	33.0	7
48.0	35.0	56	95.4	0.51	9.0	20.0	6
41.0	24.0	75	33.3	0.71	17.8	30.0	5
50.0	31.0	#NULL!	31.2	0.90	15.0	24.0	6
51.0	34.0	65	53.8	0.84	19.0	47.0	6
66.0	55.0	36	77.7	0.69	17.3	37.0	3.7
43.0	26.0	73	#NULL!	#NULL!	#NULL!	28.0	
45.0	30.0	65	63.1	1.17	29.3	29.0	6
47.0	34.0	57	#NULL!	1.04	20.0	38.0	4
51.0	37.0	57	#NULL!	0.49	12.0	64.0	5
53.0	43.0	44	61.4	0.78	26.0	#NULL!	4
52.0	35.0	64	93.5	0.82	14.1	53.0	6
53.0	38.0	58	#NULL!	1.23	35.0	27.0	5.5

46.0	28.0	72	40.0	0.72	14.0	29.0	6
51.0	33.0	68	32.3	0.66	11.0	37.0	6
54.0	35.0	67	48.0	0.77	14.0	21.0	7.8
53.0	35.0	66	#NULL!	0.75	9.4	34.0	11
61.0	46.0	#NULL!	36.2	0.67	17.0	25.0	4
50.0	34.0	63	66.8	1.03	15.0	50.0	5
42.0	28.0	#NULL!	88.5	0.92	18.3	44.0	3.5
49.0	28.0	77	40.2	0.83	17.0	40.0	7.3
53.0	35.0	66	31.8	0.78	11.1	15.0	8
50.0	39.0	49	51.8	1.31	44.0	53.0	5
58.0	44.0	#NULL!	67.2	1.40	28.0	59.0	5
39.0	26.0	65	66.5	1.30	37.1	29.0	4.8
38.0	33.0	34	#NULL!	1.70	#NULL!	51.0	2
46.0	31.0	64	#NULL!	0.75	19.0	44.0	6
44.0	29.0	66	62.3	#NULL!	#NULL!	29.0	
51.0	36.0	60	85.5	1.38	23.0	56.0	3
43.0	26.0	73	76.7	#NULL!	#NULL!	35.0	
49.0	32.0	67	41.4	1.34	22.3	32.0	
42.0	28.0	65	81.8	1.11	22.0	47.0	3
58.0	52.0	#NULL!	52.0	1.22	31.0	55.0	3
48.0	33.0	62	29.2	0.87	29.0	33.0	6
44.0	30.0	63	60.0	1.03	49.0	35.0	3.4
46.0	30.0	67	48.0	#NULL!	#NULL!	#NULL!	
58.0	37.0	#NULL!	98.0	1.38	19.7	53.0	5
47.0	29.0	71	32.9	0.88	29.0	34.0	5
45.0	39.0	#NULL!	54.8	1.18	#NULL!	59.0	
53.0	34.0	68	78.7	1.27	21.0	42.0	6
48.0	34.0	59	#NULL!	1.26	25.0	58.0	5
35.0	25.0	58	#NULL!	#NULL!	#NULL!	#NULL!	5
48.0	30.0	70	18.4	0.46	9.0	25.0	7
40.0	28.0	61	50.0	0.77	39.0	24.0	4
45.0	28.0	71	59.0	0.66	17.0	30.0	6
43.0	31.0	58	63.9	#NULL!	#NULL!	23.0	5.6
41.0	26.0	69	#NULL!	0.65	33.0	23.0	6
61.0	44.0	#NULL!	53.3	0.98	33.0	27.0	4
44.0	31.0	60	28.4	0.60	15.0	29.0	5
54.0	32.0	74	75.1	#NULL!	#NULL!	43.0	
43.0	29.0	64	54.1	0.80	16.0	45.0	7
44.0	28.0	59	#NULL!	0.43	14.0	38.0	6
54.0	37.0	58	48.3	1.25	42.0	54.0	6
58.0	46.0	37	79.3	1.12	38.0	49.0	2
52.0	35.0	64	42.6	0.76	19.0	42.0	8
51.0	34.0	65	38.3	1.11	27.8	40.0	6

42.0	27.0	68	42.5	0.59	11.8	25.0	6
45.0	24.0	81	#NULL!	#NULL!	#NULL!	44.0	
39.0	27.0	62	15.1	0.61	30.5	26.0	6
50.0	35.0	61	39.4	#NULL!	#NULL!	26.0	
52.0	36.0	62	#NULL!	1.42	36.0	37.0	6
45.0	26.0	76	39.2	#NULL!	#NULL!	85.0	
57.0	40.0	60	44.0	0.70	18.0	21.0	6
64.0	57.0	#NULL!	51.0	#NULL!	#NULL!	47.0	3
49.0	29.0	74	47.5	0.99	16.5	26.0	10
47.0	30.0	69	96.8	#NULL!	#NULL!	59.0	
49.0	32.0	67	48.3	0.71	14.0	#NULL!	7.9
63.0	42.0	65	#NULL!	1.46	24.0	36.0	8.6
54.0	42.0	44	62.9	1.26	25.0	23.0	4.5
53.0	37.0	61	48.4	1.23	19.0	38.0	7.6
48.0	35.0	56	87.3	0.57	4.5	35.0	4.8
52.0	35.0	64	49.3	1.08	21.6	26.0	8
41.0	26.0	69	#NULL!	1.20	30.0	100.0	7
44.0	28.0	69	55.0	1.23	21.0	30.0	6
46.0	29.0	70	35.7	0.76	9.5	26.0	7
39.0	24.0	72	30.3	0.78	16.0	27.0	11
54.0	33.0	72	82.0	#NULL!	#NULL!	51.0	
53.0	35.0	66	54.4	1.06	18.0	39.0	7
51.0	35.0	62	24.8	0.61	7.0	22.0	6
42.0	31.0	55	28.5	1.33	27.0	79.0	5
49.0	36.0	56	52.9	1.17	29.0	20.0	5
0.0	0.0	0	0.0	0.00	0.0	0.0	0
57.0	39.0	63	61.1	0.96	14.0	53.0	7
45.0	28.0	71	78.3	1.54	32.7	44.0	5
41.0	25.0	62	41.9	1.14	19.0	30.0	5
52.0	34.0	67	29.5	0.55	14.0	18.0	5
47.0	33.0	60	52.9	#NULL!	#NULL!	#NULL!	
52.0	32.0	72	29.3	1.17	19.5	24.0	5
44.0	26.0	75	41.0	0.86	17.0	20.0	8
53.0	31.0	75	39.0	0.88	29.3	27.0	5
51.0	34.0	65	#NULL!	1.15	21.0	31.0	8
60.0	51.0	#NULL!	79.0	1.61	32.0	64.0	7
54.0	33.0	72	68.7	0.94	23.5	23.0	5
37.0	29.0	48	21.4	0.56	9.5	25.0	6
38.0	25.0	66	54.4	0.96	24.0	#NULL!	4
51.0	30.0	75	#NULL!	0.98	13.0	25.0	7
66.0	60.0	22	45.5	0.79	16.0	19.0	2
45.0	27.0	74	31.3	0.69	14.0	35.0	7.1
46.0	33.0	58	46.3	0.96	19.0	28.0	7

58.0	39.0	64	38.4	0.88	17.6	#NULL!	6
54.0	33.0	72	#NULL!	1.27	30.0	14.0	5.7
64.0	48.0	53	52.3	1.71	#NULL!	51.0	
70.0	55.0	38	#NULL!	0.70	14.0	19.0	6
49.0	35.0	58	47.0	0.66	10.0	35.0	7
69.0	56.0	#NULL!	48.1	0.79	40.0	17.0	4
51.0	29.0	77	106.2	#NULL!	#NULL!	66.0	
44.0	27.0	72	39.3	0.76	12.1	26.0	7.2
38.0	23.0	73	44.1	1.04	26.0	26.0	11
43.0	24.0	78	21.3	0.82	14.0	29.0	6
60.0	53.0	31	76.1	0.96	13.7	47.0	4
48.0	34.0	59	30.7	1.11	28.0	26.0	5
53.0	38.0	58	58.5	1.21	25.7	53.0	3.7
46.0	29.0	#NULL!	51.7	0.92	23.0	30.0	5
47.0	29.0	71	27.8	0.81	16.0	34.0	4
51.0	33.0	68	25.2	0.63	9.8	29.0	6.1
60.0	52.0	29	#NULL!	0.99	41.0	40.0	3.3
42.0	28.0	65	46.7	0.92	18.0	26.0	7
56.0	34.0	73	61.4	#NULL!	#NULL!	35.0	9.4
52.0	36.0	62	49.1	#NULL!	#NULL!	22.0	
48.0	32.0	#NULL!	36.8	0.87	22.0	18.0	7
52.0	32.0	72	34.3	0.78	19.0	28.0	5
52.0	35.0	64	24.8	0.78	13.5	#NULL!	6.8
56.0	35.0	70	88.8	1.49	50.0	19.0	5
49.0	29.0	74	#NULL!	1.61	32.0	30.0	9
57.0	44.0	45	39.8	0.68	17.0	34.0	6
47.0	29.0	71	72.8	1.11	22.2	40.0	9
72.0	64.0	#NULL!	86.4	0.97	32.0	54.0	3
49.0	36.0	56	37.0	#NULL!	#NULL!	26.0	
46.0	27.0	75	37.2	1.00	20.0	31.0	7
47.0	38.0	#NULL!	40.4	0.72	24.0	48.0	7
52.0	37.0	59	48.6	0.81	24.6	#NULL!	6
46.0	28.0	72	19.5	0.49	9.0	23.0	7.3
60.0	54.0	29	45.6	#NULL!	#NULL!	51.0	
52.0	32.0	72	39.5	1.09	17.6	41.0	6.2
51.0	33.0	68	45.9	1.23	24.6	29.0	6
49.0	41.0	#NULL!	54.8	1.09	36.0	53.0	4
53.0	35.0	66	36.6	0.81	14.0	23.0	6
48.0	31.0	68	21.5	0.65	13.0	#NULL!	12
54.0	41.0	52	32.2	0.62	15.5	30.0	6
51.0	32.0	70	43.5	1.25	#NULL!	33.0	7
49.0	31.0	69	63.5	0.70	23.3	29.0	5
52.0	35.0	64	35.5	1.21	13.8	27.0	6.6

43.0	35.0	38	#NULL!	0.67	34.0	29.0	4
47.0	32.0	63	28.8	0.81	16.0	22.0	10
57.0	38.0	60	58.2	#NULL!	#NULL!	59.0	
44.0	24.0	80	86.8	1.84	61.3	35.0	4
64.0	45.0	60	83.0	1.37	27.0	55.0	6
66.0	57.0	#NULL!	60.0	0.84	19.0	30.0	4
53.0	36.0	63	52.4	0.80	10.0	37.0	4
48.0	32.0	65	30.6	0.49	8.0	23.0	7
43.0	26.0	73	66.3	#NULL!	#NULL!	39.0	
49.0	31.0	69	41.5	0.95	19.0	19.0	6
50.0	30.0	74	60.8	1.28	32.0	33.0	7
54.0	37.0	63	45.0	0.77	11.7	49.0	5
58.0	40.0	62	53.1	0.86	10.8	32.0	6
51.0	34.0	65	#NULL!	1.22	20.0	66.0	3
63.0	50.0	39	38.9	0.86	23.0	46.0	3.6
45.0	25.0	79	60.0	#NULL!	#NULL!	45.0	5
51.0	35.0	62	39.5	0.54	13.5	35.0	6
33.0	21.0	69	24.1	0.90	11.0	14.0	9
53.0	41.0	50	32.2	0.78	19.5	#NULL!	6
49.0	32.0	67	43.3	0.56	14.0	27.0	6
56.0	36.0	68	26.9	0.55	9.5	35.0	8.9
51.0	36.0	60	44.3	1.24	20.7	#NULL!	5
44.0	29.0	66	52.2	1.59	40.0	48.0	7
64.0	40.0	70	#NULL!	0.21	5.0	25.0	9
49.0	31.0	#NULL!	32.3	0.89	22.3	41.0	8
42.0	23.0	80	86.3	1.09	54.5	34.0	5
41.0	24.0	75	62.2	0.91	23.0	32.0	7
42.0	25.0	74	42.9	0.84	17.0	38.0	6
54.0	39.0	52	#NULL!	1.01	16.0	42.0	5.6
45.0	29.0	68	40.3	0.64	20.0	22.0	7.9
50.0	32.0	69	31.1	0.75	19.0	11.0	7.7
45.0	29.0	68	#NULL!	0.65	16.3	#NULL!	6
46.0	31.0	#NULL!	32.4	0.89	15.0	32.0	6
80.0	58.0	57	56.6	0.67	13.0	40.0	9
44.0	29.0	66	57.2	1.12	22.2	55.0	7.8
38.0	24.0	70	37.3	0.88	29.0	27.0	6
53.0	30.0	77	47.7	0.73	14.0	27.0	7.5
51.0	35.0	62	66.9	0.98	16.0	36.0	5.2
54.0	35.0	67	#NULL!	0.50	10.0	38.0	7
49.0	31.0	69	56.4	1.37	22.8	50.0	9
52.0	34.0	#NULL!	75.6	1.08	15.0	55.0	7
52.0	32.0	72	35.8	0.42	7.0	23.0	8
58.0	38.0	67	#NULL!	1.14	23.0	46.0	6

52.0	39.0	53	63.2	1.59	30.0	42.0	4
44.0	25.0	77	41.7	1.15	15.0	24.0	7
51.0	35.0	62	46.6	1.10	18.3	34.0	6
50.0	30.0	74	118.5	0.97	9.7	51.0	6
46.0	28.0	72	36.0	0.67	17.0	22.0	6
48.0	34.0	59	32.5	#NULL!	#NULL!	#NULL!	
40.0	28.0	61	27.9	0.69	23.0	27.0	5
49.0	32.0	67	27.8	0.52	10.4	21.0	6
48.0	28.0	75	47.6	0.73	14.6	29.0	7
48.0	32.0	65	41.4	0.78	20.0	32.0	5
56.0	36.0	#NULL!	48.5	1.29	20.0	30.0	7
61.0	51.0	#NULL!	68.5	1.20	24.0	57.0	4
59.0	41.0	61	58.1	1.20	30.0	51.0	6
49.0	31.0	69	55.0	#NULL!	#NULL!	82.0	4
49.0	30.0	72	43.1	0.73	18.0	18.0	6
51.0	41.0	35	38.1	0.54	14.0	23.0	6
41.0	25.0	72	50.0	1.00	20.0	33.0	6
49.0	46.0	21	33.0	0.97	#NULL!	47.0	6.6
42.0	26.0	71	28.1	0.37	8.0	35.0	6.4
51.0	35.0	62	29.2	0.83	21.0	34.0	6
64.0	54.0	38	106.2	0.99	18.7	61.0	3
63.0	55.0	#NULL!	69.5	1.02	26.0	57.0	3
52.0	30.0	76	54.5	0.93	23.0	26.0	6
70.0	55.0	38	70.4	1.30	32.0	49.0	5
48.0	31.0	68	40.7	1.19	19.8	41.0	6
53.0	42.0	#NULL!	54.1	0.90	30.0	28.0	3
47.0	27.0	#NULL!	35.4	0.68	14.0	30.0	5.7
51.0	34.0	65	62.5	1.12	37.0	42.0	3
51.0	37.0	57	#NULL!	1.30	26.0	26.0	4
51.0	37.0	52	35.5	0.90	20.0	31.0	5.5
46.0	27.0	75	60.5	0.97	12.6	34.0	7.4
70.0	64.0	16	89.6	1.29	64.5	67.0	3
55.0	31.0	78	83.8	0.96	24.0	74.0	4
45.0	29.0	68	85.2	1.20	30.0	34.0	5
40.0	29.0	57	37.2	#NULL!	#NULL!	38.0	
43.0	30.0	61	#NULL!	0.78	29.0	25.0	4.5
47.0	31.0	66	57.3	1.20	20.0	40.0	6
42.0	24.0	77	24.3	0.56	11.0	20.0	7
56.0	44.0	48	99.1	1.05	21.0	41.0	5
49.0	28.0	77	61.9	1.06	35.0	44.0	7
49.0	38.0	#NULL!	49.6	0.83	27.7	28.0	3
44.0	31.0	60	#NULL!	0.64	21.0	24.0	6
44.0	29.0	66	#NULL!	1.30	43.0	44.0	5

58.0	36.0	71	55.0	0.75	15.0	25.0	9
41.0	24.0	75	#NULL!	0.76	22.0	28.0	5.5
46.0	32.0	61	#NULL!	1.52	25.0	64.0	4
40.0	24.0	74	67.9	1.12	28.0	33.0	7
49.0	27.0	79	60.0	0.95	24.0	36.0	6
47.0	32.0	63	52.1	1.49	37.0	27.0	4.5
43.0	28.0	67	#NULL!	0.82	27.3	30.0	5
42.0	29.0	62	38.3	1.10	36.7	30.0	7
61.0	48.0	#NULL!	49.7	1.29	26.0	42.0	7
48.0	31.0	68	39.0	1.00	33.0	30.0	5
46.0	31.0	64	32.1	0.86	21.5	32.0	6
54.0	31.0	77	#NULL!	0.98	26.0	37.0	6.1
50.0	37.0	#NULL!	51.5	0.42	6.0	47.0	8
47.0	30.0	69	#NULL!	0.59	12.0	29.0	8
50.0	29.0	76	45.0	0.88	12.6	44.0	6
48.0	30.0	70	36.0	0.80	13.0	24.0	6
59.0	34.0	76	42.2	0.61	20.0	25.0	5
56.0	48.0	26	89.5	0.86	21.5	37.0	3
65.0	53.0	#NULL!	55.1	0.76	15.2	21.0	5
43.0	28.0	67	23.9	0.63	9.4	31.0	7.4
48.0	39.0	43	49.3	0.73	24.3	20.0	4
45.0	30.0	65	41.0	#NULL!	#NULL!	40.0	
44.0	25.0	77	57.5	1.01	25.0	36.0	6
46.0	27.0	75	22.8	0.92	18.3	24.0	8
43.0	29.0	64	75.4	1.20	20.0	74.0	4
52.0	35.0	64	73.6	1.28	15.4	36.0	6.4
49.0	35.0	58	#NULL!	0.48	10.0	73.0	10
64.0	44.0	62	70.4	1.64	41.0	72.0	5
47.0	30.0	69	32.2	0.92	#NULL!	36.0	5
51.0	36.0	60	#NULL!	#NULL!	#NULL!	24.0	
50.0	34.0	63	39.7	0.66	13.0	35.0	6
47.0	26.0	79	#NULL!	0.76	19.0	24.0	7
48.0	31.0	68	30.2	0.64	16.0	23.0	6
47.0	30.0	69	34.0	0.38	12.7	30.0	8
64.0	50.0	#NULL!	66.1	0.52	13.0	30.0	4
53.0	43.0	#NULL!	56.2	1.52	51.0	79.0	4
46.0	28.0	72	72.7	1.56	29.4	61.0	4.7
48.0	31.0	68	53.3	0.67	16.8	28.0	6
47.0	31.0	66	#NULL!	0.62	21.0	23.0	6.4
52.0	32.0	72	42.8	0.74	18.5	23.0	8
52.0	38.0	56	69.5	1.41	28.2	44.0	6
50.0	33.0	66	34.7	0.95	31.7	32.0	4
68.0	63.0	#NULL!	59.6	1.03	26.0	34.0	4

53.0	35.0	66	62.7	0.87	17.0	#NULL!	5
46.0	27.0	75	29.9	0.94	24.0	29.0	7
48.0	30.0	70	32.9	1.06	21.0	22.0	7
44.0	25.0	77	27.6	0.65	16.0	32.0	5
58.0	47.0	#NULL!	49.0	1.14	23.0	47.0	5
46.0	33.0	58	64.9	0.78	15.6	39.0	5
59.0	39.0	66	44.7	0.57	10.0	37.0	9
54.0	33.0	72	59.7	1.18	22.3	27.0	7.7
48.0	30.0	70	79.1	1.50	16.7	51.0	7
40.0	27.0	64	56.2	0.66	22.0	36.0	
48.0	31.0	68	75.0	#NULL!	#NULL!	34.0	5
48.0	30.0	70	#NULL!	0.96	24.0	35.0	4
52.0	34.0	67	#NULL!	0.64	24.0	30.0	5
47.0	28.0	74	31.7	0.69	24.6	28.0	6.5
38.0	23.0	73	34.1	0.70	18.0	28.0	6
46.0	31.0	64	#NULL!	0.59	14.0	36.0	7
46.0	30.0	67	42.6	0.70	12.0	27.0	8
24.0	16.0	#NULL!	13.6	#NULL!	#NULL!	#NULL!	
37.0	26.0	60	18.5	0.40	13.0	83.0	6
55.0	36.0	67	52.5	0.50	10.0	23.0	8
51.0	32.0	#NULL!	53.8	0.63	21.0	34.0	5
47.0	34.0	57	87.0	1.55	14.0	42.0	8
45.0	28.0	53	21.2	0.41	14.0	36.0	5
43.0	24.0	78	36.1	0.98	16.3	32.0	8
52.0	35.0	64	#NULL!	1.31	19.0	45.0	6
55.0	36.0	67	197.9	1.54	30.8	69.0	5
41.0	28.0	63	#NULL!	1.10	#NULL!	18.0	4.3
45.0	31.0	62	#NULL!	0.71	23.7	27.0	5
50.0	33.0	66	33.4	0.65	11.0	27.0	8
48.0	31.0	68	41.5	0.85	9.0	37.0	7
42.0	28.0	65	#NULL!	0.75	38.0	17.0	7
40.0	26.0	67	34.2	0.86	17.0	32.0	7
53.0	35.0	66	38.2	1.12	23.0	16.0	9
47.0	28.0	74	44.0	1.02	20.3	54.0	9
50.0	35.0	61	#NULL!	0.84	21.0	29.0	6
52.0	36.0	#NULL!	58.0	0.89	26.2	37.0	4.4
53.0	37.0	56	#NULL!	0.69	12.0	25.0	6.4
42.0	25.0	74	42.2	0.73	15.0	29.0	7
52.0	39.0	48	48.6	0.49	7.0	24.0	4
41.0	27.0	66	39.8	1.01	25.0	47.0	7
38.0	23.0	73	42.5	0.92	30.7	21.0	7
42.0	30.0	58	74.2	1.11	22.0	37.0	3
47.0	30.0	69	41.5	0.63	15.8	33.0	7

44.0	29.0	66	#NULL!	1.24	31.0	36.0	5.2
46.0	31.0	64	34.1	1.24	25.0	17.0	6
50.0	36.0	58	52.3	1.71	42.8	52.0	4
47.0	33.0	60	75.0	0.75	22.7	37.0	6
42.0	29.0	62	38.9	0.80	16.0	27.0	7
44.0	28.0	#NULL!	42.5	0.67	17.0	28.0	6
45.0	31.0	62	53.4	0.64	16.0	25.0	6
51.0	34.0	65	71.3	0.90	18.0	27.0	9
40.0	26.0	67	34.7	1.12	13.0	37.0	6.9
54.0	41.0	52	#NULL!	1.05	26.0	79.0	3
62.0	50.0	44	61.1	0.69	17.3	54.0	5
36.0	20.0	76	33.6	0.61	24.4	20.0	7
51.0	42.0	#NULL!	59.1	1.08	22.0	36.0	5
51.0	36.0	60	57.3	1.03	#NULL!	42.0	8
47.0	27.0	76	44.0	1.02	23.0	28.0	6.9
53.0	33.0	71	44.8	1.17	36.6	53.0	4.9
43.0	30.0	61	54.0	#NULL!	#NULL!	38.0	
50.0	31.0	71	66.9	1.04	26.0	43.0	4
47.0	34.0	57	37.5	0.62	21.0	20.0	4
45.0	29.0	68	46.2	1.00	25.0	26.0	5
48.0	33.0	62	#NULL!	1.29	22.0	40.0	5
46.0	28.0	72	147.5	1.23	18.0	52.0	5.3
49.0	34.0	#NULL!	#NULL!	1.20	20.0	29.0	5
45.0	33.0	56	40.6	0.84	12.0	27.0	6.7
48.0	38.0	#NULL!	26.7	0.79	19.8	#NULL!	6
61.0	53.0	34	58.9	0.40	13.3	47.0	2
49.0	27.0	79	62.4	1.06	26.5	32.0	6
44.0	31.0	60	32.8	0.91	30.0	22.0	5
49.0	29.0	74	#NULL!	0.87	14.3	30.0	9
49.0	32.0	67	84.2	1.08	15.0	81.0	6.8
54.0	34.0	70	#NULL!	1.10	22.0	35.0	7
40.0	28.0	61	22.2	0.60	20.0	40.0	5
47.0	27.0	76	45.8	1.39	16.0	31.0	4.4
34.0	21.0	71	49.3	#NULL!	#NULL!	23.0	
40.0	27.0	64	54.4	1.70	43.0	40.0	7
48.0	29.0	73	56.7	1.45	42.7	48.0	4
43.0	27.0	70	49.7	1.09	22.2	17.0	6.7
46.0	27.0	75	#NULL!	0.66	17.0	37.0	7
43.0	29.0	64	58.6	0.92	31.0	31.0	5
47.0	26.0	79	34.6	0.64	12.8	34.0	7
53.0	42.0	#NULL!	56.6	0.67	33.5	32.0	3
51.0	32.0	70	46.1	1.02	20.3	28.0	4
43.0	27.0	70	62.3	1.05	21.0	48.0	7

52.0	42.0	#NULL!	50.7	0.80	22.0	44.0	5.5
43.0	30.0	46	#NULL!	0.78	11.0	40.0	6
49.0	27.0	79	44.9	1.12	28.0	38.0	5
58.0	38.0	67	170.1	1.02	16.0	75.0	6.5
55.0	40.0	57	#NULL!	1.36	45.3	42.0	3
41.0	21.0	83	#NULL!	0.62	15.0	23.0	9.1
50.0	33.0	61	32.6	0.76	19.0	25.0	6
53.0	38.0	#NULL!	58.7	1.22	31.0	86.0	4
42.0	25.0	74	40.4	0.65	21.7	27.0	4
62.0	47.0	52	64.0	0.94	24.0	61.0	4
59.0	51.0	23	52.6	0.89	45.0	72.0	2
64.0	48.0	#NULL!	#NULL!	1.18	30.0	#NULL!	4
53.0	34.0	68	37.1	1.74	43.5	50.0	5
46.0	31.0	64	28.8	0.55	11.0	24.0	7
46.0	27.0	75	#NULL!	0.76	25.3	19.0	6
42.0	26.0	71	#NULL!	#NULL!	#NULL!	32.0	
49.0	36.0	56	114.3	1.78	39.6	26.0	4
48.0	30.0	70	63.1	1.09	14.0	41.0	6
49.0	33.0	64	40.6	0.60	12.0	27.0	5
45.0	30.0	65	34.8	0.72	24.0	38.0	5
45.0	27.0	74	66.7	0.65	16.0	39.0	6
60.0	47.0	43	#NULL!	1.30	22.0	65.0	2
42.0	24.0	77	47.2	1.69	46.9	59.0	3.4
52.0	38.0	56	28.8	0.76	15.2	25.0	6
50.0	34.0	63	38.6	1.18	20.0	45.0	6
45.0	28.0	71	55.9	0.70	23.0	24.0	5
50.0	35.0	61	30.4	0.59	15.0	24.0	5
41.0	27.0	66	38.8	0.85	28.0	33.0	7
51.0	28.0	79	#NULL!	0.64	21.0	46.0	5
45.0	27.0	74	55.7	1.18	18.7	50.0	8
25.0	20.0	41	46.4	1.25	25.0	37.0	5
43.0	30.0	61	50.8	1.49	32.0	29.0	6.1
49.0	30.0	72	74.6	1.45	20.7	42.0	4.5
51.0	33.0	68	#NULL!	1.40	22.0	23.0	6
57.0	49.0	#NULL!	96.5	1.27	32.0	47.0	5
51.0	34.0	65	51.3	0.67	16.8	33.0	6
50.0	31.0	71	53.6	1.48	37.0	36.0	7
52.0	32.0	#NULL!	35.3	0.51	12.0	23.0	6.6
39.0	23.0	75	37.3	0.83	41.5	47.0	4
44.0	30.0	63	76.3	1.61	32.2	53.0	6
50.0	38.0	#NULL!	49.5	0.54	14.0	24.0	4
53.0	35.0	66	55.6	1.01	16.3	36.0	8.9
49.0	32.0	67	78.9	0.77	15.0	54.0	5

38.0	24.0	70	58.5	1.51	25.0	42.0	4
45.0	30.0	65	78.4	0.60	15.0	20.0	5
41.0	27.0	66	36.0	0.63	16.0	31.0	6
43.0	25.0	76	35.5	0.97	19.0	44.0	6
43.0	28.0	67	29.0	0.69	13.8	22.0	6
48.0	30.0	70	#NULL!	1.25	25.0	52.0	5
50.0	34.0	#NULL!	77.5	0.80	11.0	27.0	4
44.0	28.0	69	20.3	0.56	11.2	#NULL!	7
52.0	34.0	67	60.4	0.73	11.0	37.0	7
49.0	37.0	52	90.4	0.93	12.0	37.0	5
51.0	32.0	70	32.8	0.59	30.0	27.0	5
43.0	26.0	73	59.4	1.10	28.0	39.0	5
53.0	34.0	68	55.8	1.32	26.0	47.0	6.4
38.0	24.0	70	34.3	0.83	16.6	49.0	4
46.0	32.0	61	63.0	#NULL!	#NULL!	48.0	
46.0	30.0	67	61.4	1.51	38.0	46.0	6
39.0	20.0	83	25.5	0.90	22.5	20.0	7
51.0	35.0	62	78.4	0.89	18.0	66.0	3
43.0	26.0	73	38.1	1.17	39.0	40.0	7
41.0	23.0	78	50.3	1.05	26.0	27.0	3
49.0	34.0	61	35.5	1.08	21.6	29.0	6
55.0	43.0	43	48.3	0.63	21.0	43.0	6
49.0	34.0	61	26.6	0.55	13.8	23.0	7
48.0	32.0	65	#NULL!	#NULL!	#NULL!	52.0	
64.0	44.0	#NULL!	89.4	0.96	16.0	62.0	6
53.0	34.0	#NULL!	74.6	0.90	30.0	58.0	4
47.0	34.0	57	49.4	0.77	26.0	53.0	3
36.0	22.0	72	70.7	0.96	24.0	39.0	3
62.0	52.0	#NULL!	109.8	0.54	14.0	40.0	4
42.0	27.0	68	#NULL!	0.84	28.0	28.0	6
51.0	37.0	57	#NULL!	1.62	27.0	37.0	6

HTN	DM	Dyslip	CAD	PCI	CABG	CKD	ESRD
1.0	1.0	0.0	0.0	0.0	0.0	0.0	0.0
1.0	0.0	0.0	0.0	1.0	0.0	0.0	1.0
1.0	0.0	0.0	0.0	0.0	0.0	0.0	0.0
1.0	1.0	0.0	0.0	0.0	0.0	0.0	0.0
1.0	1.0	1.0	0.0	0.0	0.0	1.0	0.0
1.0	0.0	0.0	1.0	0.0	0.0	0.0	1.0
1.0	1.0	0.0	0.0	0.0	0.0	0.0	1.0
1.0	1.0	0.0	1.0	1.0	0.0	0.0	1.0
1.0	0.0	0.0	0.0	0.0	0.0	0.0	1.0
1.0	1.0	0.0	1.0	1.0	0.0	0.0	1.0
1.0	1.0	0.0	1.0	1.0	0.0	0.0	1.0
1.0	0.0	1.0	1.0	0.0	0.0	0.0	0.0
1.0	1.0	0.0	1.0	0.0	0.0	0.0	0.0
1.0	1.0	1.0	1.0	0.0	0.0	0.0	1.0
1.0	1.0	0.0	1.0	0.0	0.0	0.0	1.0
1.0	0.0	1.0	0.0	1.0	0.0	0.0	0.0
1.0	0.0	0.0	0.0	0.0	0.0	0.0	0.0
1.0	1.0	0.0	0.0	1.0	0.0	0.0	1.0
1.0	0.0	0.0	0.0	0.0	0.0	0.0	0.0
1.0	1.0	0.0	1.0	0.0	1.0	0.0	0.0
1.0	1.0	0.0	0.0	0.0	0.0	0.0	0.0
1.0	1.0	0.0	0.0	0.0	0.0	0.0	1.0
1.0	0.0	1.0	0.0	0.0	0.0	0.0	0.0
1.0	1.0	0.0	0.0	0.0	0.0	0.0	0.0
1.0	1.0	0.0	0.0	0.0	0.0	1.0	0.0
1.0	1.0	1.0	1.0	0.0	1.0	0.0	0.0
1.0	1.0	1.0	0.0	0.0	0.0	0.0	0.0
1.0	1.0	0.0	1.0	1.0	0.0	1.0	0.0
0.0	1.0	0.0	0.0	0.0	0.0	0.0	0.0
0.0	1.0	0.0	1.0	1.0	0.0	0.0	1.0
1.0	0.0	0.0	0.0	0.0	0.0	1.0	0.0
1.0	0.0	1.0	1.0	1.0	0.0	0.0	0.0
1.0	1.0	0.0	0.0	0.0	0.0	0.0	0.0
1.0	0.0	1.0	0.0	0.0	0.0	0.0	0.0
1.0	0.0	0.0	0.0	0.0	0.0	0.0	0.0
0.0	0.0	0.0	1.0	0.0	1.0	0.0	0.0
0.0	0.0	1.0	1.0	0.0	0.0	0.0	0.0
1.0	0.0	0.0	0.0	0.0	0.0	0.0	0.0
1.0	0.0	0.0	0.0	0.0	0.0	0.0	0.0
1.0	1.0	0.0	0.0	0.0	0.0	0.0	1.0
1.0	0.0	0.0	0.0	0.0	0.0	0.0	0.0
1.0	0.0	1.0	1.0	0.0	1.0	0.0	0.0

1.0	1.0	0.0	1.0	1.0	0.0	0.0	0.0
1.0	0.0	1.0	0.0	0.0	0.0	0.0	0.0
1.0	1.0	0.0	0.0	0.0	0.0	1.0	0.0
1.0	0.0	1.0	0.0	0.0	0.0	0.0	0.0
1.0	0.0	0.0	0.0	0.0	0.0	0.0	0.0
0.0	0.0	1.0	0.0	0.0	0.0	0.0	0.0
0.0	0.0	0.0	0.0	0.0	0.0	0.0	0.0
1.0	0.0	0.0	0.0	0.0	0.0	0.0	0.0
1.0	0.0	0.0	1.0	0.0	0.0	1.0	0.0
0.0	0.0	0.0	0.0	0.0	0.0	0.0	0.0
1.0	1.0	0.0	1.0	1.0	1.0	0.0	0.0
1.0	0.0	0.0	0.0	0.0	0.0	0.0	0.0
1.0	0.0	0.0	0.0	0.0	0.0	0.0	0.0
1.0	1.0	0.0	1.0	0.0	0.0	0.0	1.0
1.0	0.0	1.0	0.0	0.0	0.0	1.0	0.0
1.0	1.0	1.0	1.0	0.0	0.0	1.0	0.0
1.0	0.0	0.0	1.0	0.0	0.0	0.0	0.0
1.0	1.0	1.0	0.0	0.0	0.0	0.0	0.0
1.0	1.0	0.0	1.0	0.0	0.0	0.0	0.0
1.0	1.0	0.0	0.0	0.0	0.0	0.0	0.0
1.0	1.0	1.0	0.0	1.0	0.0	0.0	0.0
1.0	0.0	0.0	0.0	0.0	0.0	0.0	0.0
1.0	0.0	0.0	0.0	0.0	0.0	0.0	0.0
1.0	1.0	0.0	0.0	0.0	0.0	0.0	1.0
1.0	1.0	1.0	1.0	0.0	0.0	0.0	0.0
1.0	1.0	0.0	0.0	0.0	0.0	0.0	0.0
1.0	0.0	0.0	1.0	0.0	0.0	0.0	0.0
1.0	1.0	0.0	0.0	0.0	0.0	1.0	0.0
1.0	1.0	0.0	1.0	1.0	0.0	0.0	0.0
1.0	0.0	1.0	0.0	0.0	0.0	1.0	0.0
0.0	0.0	0.0	0.0	0.0	0.0	0.0	0.0
1.0	0.0	0.0	0.0	0.0	0.0	0.0	0.0
1.0	1.0	0.0	1.0	1.0	0.0	1.0	0.0
1.0	0.0	0.0	0.0	0.0	0.0	0.0	0.0
0.0	0.0	0.0	0.0	0.0	0.0	0.0	0.0
1.0	1.0	0.0	0.0	0.0	0.0	0.0	0.0
1.0	1.0	0.0	0.0	0.0	0.0	0.0	0.0
1.0	0.0	0.0	0.0	0.0	0.0	1.0	0.0
1.0	0.0	0.0	1.0	1.0	0.0	0.0	0.0
1.0	1.0	1.0	1.0	1.0	0.0	0.0	0.0
1.0	1.0	0.0	0.0	0.0	0.0	1.0	0.0
1.0	1.0	1.0	0.0	0.0	0.0	1.0	0.0
1.0	1.0	0.0	0.0	0.0	0.0	0.0	0.0

1.0	1.0	1.0	1.0	1.0	0.0	0.0	0.0
1.0	0.0	0.0	0.0	0.0	0.0	0.0	0.0
1.0	0.0	0.0	0.0	0.0	0.0	1.0	0.0
1.0	0.0	0.0	0.0	0.0	0.0	0.0	1.0
1.0	1.0	0.0	0.0	0.0	0.0	0.0	1.0
1.0	0.0	0.0	0.0	0.0	0.0	0.0	1.0
1.0	0.0	0.0	0.0	0.0	0.0	0.0	1.0
0.0	0.0	0.0	0.0	0.0	0.0	1.0	0.0
1.0	0.0	0.0	0.0	0.0	0.0	0.0	1.0
1.0	0.0	0.0	0.0	0.0	0.0	0.0	1.0
1.0	0.0	0.0	0.0	0.0	0.0	0.0	1.0
0.0	0.0	0.0	0.0	0.0	0.0	0.0	1.0
1.0	0.0	0.0	0.0	0.0	0.0	0.0	1.0
1.0	1.0	1.0	0.0	0.0	0.0	0.0	1.0
1.0	0.0	0.0	0.0	0.0	0.0	0.0	0.0
1.0	0.0	0.0	0.0	0.0	0.0	0.0	1.0
1.0	0.0	0.0	0.0	0.0	0.0	0.0	1.0
1.0	0.0	1.0	1.0	0.0	1.0	0.0	1.0
0.0	0.0	0.0	1.0	0.0	0.0	0.0	0.0
1.0	0.0	0.0	0.0	0.0	0.0	0.0	1.0
1.0	1.0	0.0	0.0	0.0	0.0	0.0	1.0
0.0	1.0	0.0	0.0	0.0	0.0	0.0	1.0
0.0	1.0	0.0	0.0	0.0	0.0	0.0	0.0
1.0	0.0	1.0	1.0	1.0	0.0	0.0	0.0
1.0	1.0	0.0	1.0	1.0	0.0	0.0	1.0
0.0	0.0	0.0	0.0	0.0	0.0	0.0	0.0
1.0	0.0	0.0	0.0	0.0	0.0	0.0	1.0
1.0	1.0	1.0	0.0	1.0	0.0	0.0	0.0
1.0	1.0	1.0	1.0	0.0	1.0	0.0	1.0
0.0	0.0	1.0	1.0	0.0	1.0	0.0	0.0
1.0	1.0	0.0	0.0	0.0	0.0	0.0	1.0
1.0	1.0	1.0	1.0	1.0	0.0	0.0	1.0
1.0	0.0	0.0	0.0	0.0	0.0	0.0	1.0
1.0	0.0	1.0	0.0	0.0	0.0	0.0	0.0
1.0	1.0	0.0	0.0	0.0	0.0	0.0	0.0
1.0	1.0	1.0	1.0	0.0	0.0	0.0	1.0
1.0	0.0	0.0	0.0	0.0	0.0	0.0	1.0
1.0	1.0	1.0	1.0	0.0	0.0	0.0	1.0
1.0	1.0	0.0	0.0	1.0	0.0	0.0	0.0
1.0	1.0	1.0	1.0	0.0	0.0	0.0	0.0
1.0	1.0	0.0	1.0	0.0	0.0	0.0	0.0
1.0	1.0	1.0	0.0	0.0	0.0	0.0	0.0
0.0	0.0	0.0	0.0	0.0	0.0	0.0	1.0

1.0	1.0	1.0	0.0	0.0	0.0	0.0	1.0
1.0	1.0	0.0	1.0	1.0	0.0	1.0	0.0
0.0	0.0	0.0	1.0	0.0	0.0	0.0	1.0
0.0	1.0	0.0	0.0	0.0	0.0	0.0	0.0
1.0	0.0	0.0	1.0	0.0	0.0	0.0	1.0
1.0	1.0	0.0	1.0	1.0	0.0	0.0	1.0
1.0	0.0	0.0	1.0	1.0	0.0	0.0	1.0
0.0	0.0	0.0	0.0	0.0	0.0	0.0	0.0
0.0	0.0	0.0	0.0	0.0	0.0	0.0	1.0
0.0	0.0	0.0	0.0	0.0	0.0	0.0	0.0
1.0	0.0	0.0	0.0	0.0	0.0	0.0	0.0
1.0	1.0	0.0	0.0	0.0	0.0	1.0	0.0
1.0	0.0	0.0	0.0	0.0	0.0	0.0	1.0
1.0	1.0	1.0	0.0	0.0	0.0	0.0	0.0
1.0	1.0	0.0	0.0	0.0	0.0	0.0	0.0
1.0	0.0	1.0	0.0	0.0	0.0	0.0	0.0
0.0	1.0	0.0	1.0	0.0	0.0	0.0	0.0
1.0	0.0	0.0	0.0	0.0	0.0	0.0	1.0
0.0	0.0	0.0	0.0	0.0	0.0	0.0	1.0
1.0	0.0	0.0	0.0	0.0	0.0	0.0	0.0
1.0	1.0	0.0	1.0	0.0	0.0	0.0	0.0
1.0	0.0	1.0	1.0	1.0	0.0	0.0	0.0
1.0	0.0	1.0	1.0	0.0	0.0	0.0	0.0
0.0	1.0	0.0	1.0	0.0	0.0	0.0	1.0
1.0	1.0	0.0	0.0	0.0	0.0	0.0	1.0
0.0	0.0	1.0	1.0	1.0	0.0	0.0	0.0
0.0	1.0	0.0	0.0	0.0	0.0	0.0	0.0
0.0	0.0	1.0	1.0	0.0	0.0	0.0	0.0
1.0	1.0	0.0	0.0	0.0	0.0	0.0	0.0
1.0	1.0	0.0	1.0	0.0	1.0	0.0	0.0
1.0	1.0	0.0	0.0	0.0	0.0	0.0	1.0
1.0	1.0	0.0	1.0	0.0	0.0	0.0	1.0
1.0	1.0	1.0	1.0	1.0	0.0	0.0	0.0
1.0	0.0	1.0	1.0	0.0	1.0	0.0	0.0
1.0	0.0	0.0	0.0	0.0	0.0	0.0	1.0
1.0	0.0	0.0	0.0	0.0	0.0	0.0	0.0
1.0	0.0	0.0	1.0	0.0	0.0	0.0	0.0
0.0	0.0	0.0	0.0	0.0	0.0	0.0	0.0
1.0	1.0	0.0	0.0	0.0	0.0	0.0	0.0
1.0	0.0	0.0	0.0	0.0	0.0	0.0	1.0
1.0	0.0	1.0	0.0	0.0	0.0	0.0	1.0
1.0	1.0	0.0	0.0	0.0	0.0	0.0	0.0
1.0	1.0	0.0	0.0	0.0	0.0	0.0	0.0

1.0	0.0	0.0	0.0	0.0	0.0	0.0	1.0
0.0	0.0	0.0	0.0	0.0	0.0	0.0	0.0
1.0	1.0	0.0	0.0	0.0	0.0	0.0	1.0
0.0	0.0	0.0	0.0	0.0	0.0	0.0	0.0
1.0	0.0	0.0	0.0	0.0	0.0	0.0	1.0
0.0	0.0	0.0	0.0	0.0	0.0	0.0	0.0
1.0	1.0	0.0	1.0	0.0	0.0	0.0	0.0
0.0	1.0	1.0	1.0	0.0	0.0	0.0	0.0
1.0	0.0	1.0	1.0	1.0	0.0	0.0	0.0
1.0	1.0	0.0	0.0	0.0	0.0	1.0	0.0
1.0	0.0	0.0	0.0	0.0	0.0	1.0	0.0
1.0	1.0	0.0	0.0	0.0	0.0	0.0	1.0
1.0	0.0	1.0	0.0	0.0	0.0	0.0	0.0
1.0	0.0	0.0	0.0	0.0	0.0	0.0	0.0
1.0	1.0	1.0	0.0	0.0	0.0	1.0	0.0
1.0	0.0	0.0	0.0	0.0	0.0	0.0	1.0
1.0	0.0	0.0	0.0	0.0	0.0	1.0	0.0
0.0	0.0	0.0	0.0	0.0	0.0	0.0	1.0
1.0	1.0	0.0	0.0	0.0	0.0	1.0	0.0
1.0	1.0	1.0	1.0	0.0	0.0	0.0	0.0
1.0	0.0	0.0	0.0	0.0	0.0	0.0	0.0
1.0	1.0	0.0	1.0	1.0	0.0	1.0	0.0
1.0	1.0	0.0	1.0	1.0	0.0	1.0	0.0
1.0	1.0	0.0	0.0	0.0	0.0	0.0	0.0
1.0	1.0	0.0	1.0	1.0	0.0	1.0	0.0
1.0	0.0	0.0	0.0	0.0	0.0	0.0	0.0
0.0	1.0	0.0	0.0	0.0	0.0	0.0	0.0
0.0	0.0	0.0	1.0	0.0	0.0	0.0	0.0
1.0	1.0	0.0	0.0	0.0	0.0	0.0	0.0
1.0	0.0	0.0	0.0	0.0	0.0	0.0	0.0
1.0	0.0	0.0	0.0	0.0	0.0	0.0	1.0
1.0	1.0	1.0	1.0	1.0	0.0	0.0	0.0
0.0	1.0	0.0	0.0	0.0	0.0	0.0	0.0
1.0	0.0	0.0	0.0	0.0	0.0	0.0	1.0
1.0	0.0	0.0	0.0	0.0	0.0	0.0	1.0
1.0	1.0	1.0	0.0	0.0	0.0	0.0	0.0
1.0	0.0	0.0	0.0	0.0	0.0	0.0	0.0
1.0	1.0	0.0	0.0	0.0	0.0	0.0	0.0
1.0	0.0	1.0	0.0	0.0	0.0	0.0	0.0
1.0	0.0	0.0	0.0	0.0	0.0	0.0	0.0
1.0	0.0	0.0	0.0	0.0	0.0	0.0	0.0
1.0	0.0	0.0	0.0	0.0	0.0	0.0	0.0
1.0	1.0	0.0	0.0	0.0	0.0	0.0	0.0

1.0	1.0	0.0	0.0	0.0	0.0	0.0	1.0
1.0	0.0	1.0	0.0	0.0	0.0	0.0	0.0
1.0	0.0	1.0	1.0	1.0	0.0	0.0	1.0
1.0	0.0	1.0	0.0	0.0	0.0	0.0	0.0
1.0	0.0	1.0	1.0	0.0	0.0	0.0	0.0
1.0	1.0	1.0	0.0	0.0	0.0	0.0	0.0
1.0	0.0	0.0	0.0	0.0	0.0	0.0	0.0
1.0	1.0	0.0	0.0	0.0	0.0	0.0	0.0
1.0	1.0	1.0	1.0	0.0	0.0	0.0	0.0
1.0	0.0	0.0	1.0	1.0	0.0	0.0	0.0
1.0	0.0	1.0	1.0	0.0	0.0	0.0	1.0
1.0	1.0	1.0	1.0	1.0	1.0	0.0	1.0
1.0	0.0	0.0	0.0	0.0	0.0	1.0	0.0
0.0	1.0	0.0	0.0	0.0	0.0	0.0	0.0
1.0	1.0	1.0	0.0	0.0	0.0	0.0	0.0
1.0	1.0	0.0	1.0	1.0	0.0	0.0	0.0
0.0	0.0	0.0	0.0	0.0	0.0	0.0	0.0
1.0	1.0	0.0	0.0	0.0	0.0	0.0	0.0
1.0	0.0	0.0	0.0	0.0	0.0	0.0	1.0
0.0	1.0	1.0	0.0	0.0	0.0	0.0	0.0
1.0	0.0	1.0	1.0	0.0	0.0	1.0	0.0
1.0	1.0	0.0	1.0	1.0	0.0	0.0	1.0
1.0	0.0	1.0	0.0	0.0	0.0	0.0	0.0
1.0	0.0	0.0	1.0	0.0	0.0	0.0	0.0
1.0	1.0	0.0	0.0	0.0	0.0	0.0	0.0
1.0	1.0	0.0	1.0	0.0	1.0	0.0	0.0
1.0	0.0	1.0	1.0	1.0	0.0	0.0	0.0
0.0	0.0	1.0	0.0	0.0	0.0	0.0	0.0
0.0	0.0	0.0	0.0	0.0	0.0	0.0	0.0
1.0	0.0	0.0	0.0	0.0	0.0	0.0	0.0
1.0	0.0	0.0	0.0	0.0	0.0	0.0	0.0
1.0	1.0	1.0	0.0	0.0	0.0	0.0	0.0
1.0	0.0	0.0	1.0	0.0	0.0	0.0	0.0
1.0	0.0	1.0	0.0	0.0	0.0	0.0	0.0
0.0	1.0	0.0	0.0	0.0	0.0	0.0	0.0
1.0	0.0	1.0	0.0	0.0	0.0	0.0	0.0
1.0	0.0	0.0	1.0	0.0	0.0	0.0	0.0
1.0	0.0	0.0	0.0	0.0	0.0	0.0	0.0
1.0	0.0	0.0	0.0	0.0	0.0	0.0	0.0
1.0	1.0	0.0	0.0	0.0	0.0	0.0	0.0
1.0	1.0	0.0	1.0	1.0	1.0	0.0	0.0
1.0	1.0	0.0	1.0	0.0	0.0	0.0	0.0
1.0	1.0	1.0	0.0	0.0	0.0	1.0	0.0

1.0	0.0	0.0	0.0	0.0	0.0	0.0	0.0
0.0	1.0	0.0	1.0	1.0	0.0	1.0	0.0
1.0	0.0	0.0	0.0	0.0	0.0	0.0	0.0
1.0	0.0	0.0	0.0	0.0	0.0	0.0	0.0
1.0	0.0	0.0	0.0	0.0	0.0	0.0	0.0
1.0	0.0	0.0	0.0	0.0	0.0	0.0	0.0
1.0	0.0	1.0	0.0	0.0	0.0	0.0	0.0
1.0	0.0	0.0	0.0	0.0	0.0	0.0	0.0
1.0	1.0	1.0	1.0	1.0	0.0	1.0	0.0
1.0	0.0	0.0	0.0	0.0	0.0	0.0	0.0
1.0	0.0	0.0	0.0	0.0	0.0	0.0	0.0
1.0	0.0	1.0	0.0	0.0	0.0	0.0	0.0
1.0	0.0	0.0	1.0	0.0	1.0	0.0	0.0
1.0	0.0	0.0	0.0	0.0	0.0	0.0	0.0
1.0	1.0	1.0	1.0	1.0	0.0	0.0	0.0
1.0	0.0	0.0	0.0	0.0	0.0	0.0	0.0
1.0	0.0	0.0	0.0	0.0	0.0	1.0	0.0
1.0	0.0	0.0	0.0	0.0	0.0	0.0	0.0
0.0	0.0	0.0	0.0	0.0	0.0	0.0	0.0
0.0	0.0	0.0	0.0	0.0	0.0	0.0	0.0
1.0	1.0	0.0	1.0	1.0	0.0	1.0	0.0
1.0	0.0	0.0	0.0	0.0	0.0	0.0	1.0
1.0	1.0	1.0	0.0	0.0	0.0	0.0	0.0
1.0	1.0	1.0	0.0	0.0	0.0	0.0	0.0
1.0	0.0	0.0	0.0	0.0	0.0	0.0	0.0
0.0	0.0	1.0	0.0	0.0	0.0	0.0	0.0
1.0	0.0	0.0	0.0	0.0	0.0	0.0	0.0
1.0	1.0	0.0	1.0	1.0	0.0	1.0	0.0
1.0	0.0	0.0	0.0	0.0	0.0	0.0	0.0
1.0	0.0	1.0	0.0	0.0	0.0	1.0	0.0
0.0	0.0	1.0	0.0	0.0	0.0	0.0	0.0
1.0	1.0	1.0	0.0	0.0	0.0	0.0	0.0
1.0	1.0	0.0	0.0	0.0	0.0	0.0	0.0
1.0	0.0	0.0	0.0	0.0	0.0	0.0	0.0
0.0	1.0	0.0	1.0	1.0	0.0	0.0	0.0
1.0	1.0	0.0	1.0	1.0	0.0	0.0	0.0
1.0	0.0	1.0	0.0	0.0	0.0	0.0	1.0
0.0	1.0	1.0	0.0	0.0	0.0	0.0	0.0
1.0	0.0	0.0	1.0	0.0	0.0	0.0	0.0
0.0	0.0	0.0	0.0	0.0	0.0	0.0	0.0
1.0	1.0	0.0	1.0	0.0	1.0	0.0	1.0
0.0	0.0	0.0	0.0	0.0	0.0	0.0	0.0
0.0	1.0	0.0	1.0	1.0	0.0	1.0	0.0

0.0	0.0	0.0	0.0	0.0	0.0	1.0	0.0
1.0	1.0	0.0	0.0	0.0	0.0	0.0	1.0
1.0	0.0	1.0	0.0	0.0	0.0	0.0	0.0
1.0	0.0	1.0	1.0	0.0	0.0	0.0	0.0
0.0	0.0	0.0	1.0	1.0	0.0	1.0	0.0
1.0	0.0	0.0	0.0	0.0	0.0	0.0	0.0
1.0	0.0	0.0	0.0	0.0	0.0	1.0	0.0
1.0	1.0	0.0	0.0	0.0	0.0	0.0	0.0
1.0	0.0	1.0	1.0	0.0	0.0	0.0	0.0
1.0	1.0	1.0	0.0	0.0	0.0	0.0	0.0
1.0	0.0	0.0	1.0	1.0	0.0	0.0	0.0
1.0	0.0	0.0	0.0	0.0	0.0	0.0	0.0
1.0	1.0	0.0	0.0	0.0	0.0	0.0	0.0
1.0	0.0	0.0	0.0	0.0	0.0	0.0	0.0
1.0	1.0	0.0	0.0	0.0	0.0	0.0	0.0
1.0	0.0	0.0	0.0	0.0	0.0	0.0	0.0
1.0	0.0	1.0	0.0	0.0	0.0	0.0	0.0
0.0	0.0	0.0	0.0	0.0	0.0	0.0	0.0
1.0	0.0	0.0	0.0	0.0	0.0	0.0	0.0
1.0	0.0	0.0	0.0	0.0	0.0	0.0	0.0
1.0	1.0	0.0	1.0	1.0	0.0	0.0	0.0
1.0	0.0	0.0	0.0	0.0	0.0	0.0	0.0
0.0	0.0	1.0	1.0	1.0	0.0	0.0	0.0
0.0	0.0	0.0	0.0	0.0	0.0	0.0	0.0
1.0	0.0	0.0	0.0	0.0	0.0	0.0	0.0
1.0	0.0	0.0	0.0	0.0	0.0	0.0	0.0
1.0	1.0	0.0	1.0	0.0	0.0	0.0	0.0
1.0	0.0	1.0	1.0	0.0	0.0	0.0	0.0
1.0	1.0	0.0	1.0	0.0	0.0	0.0	0.0
1.0	0.0	0.0	0.0	0.0	0.0	1.0	0.0
1.0	1.0	0.0	0.0	0.0	0.0	0.0	0.0
0.0	0.0	0.0	0.0	0.0	0.0	0.0	0.0
1.0	1.0	0.0	1.0	1.0	0.0	0.0	1.0
1.0	1.0	0.0	0.0	0.0	0.0	0.0	0.0
1.0	0.0	0.0	0.0	0.0	0.0	0.0	0.0
1.0	1.0	1.0	1.0	1.0	0.0	1.0	0.0
1.0	1.0	0.0	1.0	0.0	0.0	1.0	0.0
1.0	0.0	1.0	1.0	1.0	0.0	0.0	0.0
1.0	1.0	0.0	0.0	0.0	0.0	0.0	0.0
1.0	0.0	0.0	0.0	0.0	0.0	0.0	0.0
1.0	1.0	0.0	0.0	0.0	0.0	0.0	0.0
1.0	0.0	0.0	1.0	0.0	0.0	0.0	0.0
1.0	0.0	0.0	0.0	0.0	0.0	0.0	0.0

1.0	0.0	0.0	0.0	0.0	0.0	0.0	0.0
1.0	1.0	1.0	1.0	1.0	0.0	1.0	0.0
1.0	1.0	0.0	0.0	0.0	0.0	0.0	1.0
1.0	0.0	0.0	0.0	0.0	0.0	1.0	0.0
1.0	0.0	1.0	1.0	0.0	1.0	0.0	0.0
0.0	0.0	0.0	1.0	0.0	1.0	0.0	0.0
1.0	1.0	0.0	0.0	0.0	0.0	0.0	0.0
1.0	1.0	1.0	0.0	0.0	0.0	0.0	1.0
1.0	1.0	0.0	0.0	0.0	0.0	0.0	0.0
1.0	0.0	0.0	0.0	0.0	0.0	1.0	0.0
0.0	1.0	0.0	0.0	0.0	0.0	1.0	0.0
1.0	1.0	0.0	0.0	0.0	0.0	0.0	0.0
1.0	1.0	0.0	0.0	0.0	0.0	0.0	0.0
1.0	1.0	0.0	0.0	0.0	0.0	1.0	0.0
1.0	1.0	1.0	1.0	1.0	0.0	0.0	0.0
1.0	0.0	0.0	0.0	0.0	0.0	0.0	0.0
1.0	0.0	1.0	0.0	0.0	0.0	0.0	0.0
0.0	1.0	0.0	1.0	0.0	0.0	1.0	0.0
1.0	1.0	0.0	0.0	0.0	0.0	0.0	0.0
1.0	0.0	0.0	0.0	0.0	0.0	0.0	0.0
1.0	0.0	0.0	1.0	0.0	1.0	0.0	0.0
1.0	0.0	0.0	0.0	0.0	0.0	1.0	0.0
1.0	1.0	1.0	0.0	0.0	0.0	0.0	0.0
1.0	1.0	0.0	0.0	0.0	0.0	0.0	0.0
1.0	1.0	1.0	1.0	0.0	0.0	0.0	0.0
1.0	0.0	0.0	0.0	0.0	0.0	1.0	0.0
1.0	1.0	1.0	0.0	0.0	0.0	0.0	0.0
1.0	0.0	1.0	0.0	0.0	0.0	0.0	0.0
1.0	1.0	0.0	0.0	0.0	0.0	1.0	0.0
1.0	0.0	0.0	0.0	0.0	0.0	0.0	0.0
1.0	0.0	1.0	1.0	1.0	0.0	0.0	0.0
1.0	1.0	0.0	1.0	1.0	0.0	0.0	0.0
1.0	1.0	1.0	0.0	0.0	0.0	1.0	0.0
1.0	1.0	1.0	1.0	0.0	0.0	0.0	0.0
1.0	1.0	1.0	0.0	0.0	0.0	1.0	0.0
1.0	1.0	0.0	1.0	0.0	0.0	1.0	0.0
1.0	0.0	0.0	0.0	0.0	0.0	1.0	0.0
1.0	0.0	0.0	0.0	0.0	0.0	0.0	0.0
0.0	0.0	1.0	1.0	0.0	0.0	0.0	0.0
1.0	0.0	0.0	0.0	0.0	0.0	0.0	0.0
1.0	1.0	0.0	1.0	0.0	0.0	0.0	0.0
1.0	1.0	0.0	0.0	0.0	0.0	0.0	1.0
1.0	0.0	0.0	0.0	0.0	0.0	0.0	0.0

1.0	0.0	0.0	0.0	0.0	0.0	0.0	0.0
0.0	1.0	0.0	1.0	1.0	0.0	0.0	0.0
1.0	0.0	0.0	0.0	0.0	0.0	0.0	0.0
1.0	0.0	0.0	0.0	0.0	0.0	0.0	0.0
0.0	0.0	0.0	0.0	0.0	0.0	0.0	0.0
0.0	0.0	0.0	0.0	0.0	0.0	0.0	0.0
1.0	1.0	1.0	1.0	0.0	0.0	1.0	0.0
1.0	1.0	0.0	1.0	0.0	1.0	1.0	0.0
0.0	0.0	0.0	0.0	0.0	0.0	0.0	0.0
1.0	0.0	0.0	0.0	0.0	0.0	0.0	0.0
1.0	1.0	0.0	1.0	1.0	0.0	0.0	0.0
1.0	1.0	1.0	1.0	1.0	0.0	1.0	0.0
0.0	1.0	0.0	0.0	0.0	0.0	0.0	0.0
1.0	1.0	0.0	1.0	0.0	0.0	0.0	0.0
1.0	1.0	1.0	1.0	1.0	0.0	0.0	0.0
1.0	0.0	0.0	0.0	0.0	0.0	0.0	0.0
1.0	0.0	0.0	1.0	1.0	0.0	0.0	1.0
1.0	0.0	1.0	1.0	1.0	0.0	0.0	0.0
0.0	0.0	0.0	0.0	0.0	0.0	0.0	0.0
0.0	0.0	1.0	0.0	0.0	0.0	0.0	0.0
1.0	0.0	0.0	0.0	0.0	0.0	0.0	0.0
1.0	1.0	0.0	1.0	0.0	1.0	1.0	1.0
0.0	0.0	0.0	0.0	0.0	0.0	0.0	0.0
0.0	1.0	0.0	0.0	0.0	0.0	0.0	0.0
1.0	1.0	1.0	0.0	0.0	0.0	0.0	0.0
1.0	0.0	1.0	1.0	1.0	0.0	0.0	0.0
0.0	1.0	0.0	0.0	0.0	0.0	0.0	0.0
1.0	1.0	0.0	0.0	0.0	0.0	0.0	0.0
1.0	0.0	1.0	0.0	0.0	0.0	0.0	0.0
1.0	0.0	1.0	0.0	0.0	0.0	0.0	0.0
1.0	0.0	0.0	0.0	0.0	0.0	0.0	0.0
1.0	1.0	0.0	0.0	0.0	0.0	0.0	0.0
1.0	0.0	0.0	0.0	0.0	0.0	0.0	0.0
1.0	0.0	0.0	0.0	0.0	0.0	0.0	0.0
1.0	0.0	0.0	0.0	0.0	0.0	0.0	0.0
1.0	0.0	0.0	0.0	0.0	0.0	1.0	0.0
1.0	1.0	0.0	0.0	0.0	0.0	1.0	0.0
0.0	0.0	0.0	1.0	0.0	0.0	0.0	0.0
1.0	0.0	0.0	0.0	0.0	0.0	0.0	0.0
1.0	1.0	0.0	0.0	0.0	0.0	0.0	1.0
1.0	0.0	1.0	0.0	0.0	0.0	0.0	0.0
1.0	1.0	0.0	0.0	0.0	0.0	0.0	0.0
1.0	0.0	0.0	0.0	0.0	0.0	0.0	0.0

0.0	1.0	0.0	0.0	0.0	0.0	1.0	0.0
1.0	1.0	0.0	0.0	0.0	0.0	0.0	0.0
1.0	1.0	0.0	0.0	0.0	0.0	0.0	0.0
1.0	0.0	0.0	1.0	1.0	0.0	0.0	0.0
0.0	1.0	0.0	0.0	0.0	0.0	0.0	0.0
1.0	0.0	0.0	0.0	0.0	0.0	0.0	0.0
1.0	0.0	1.0	1.0	0.0	0.0	1.0	0.0
1.0	1.0	1.0	0.0	0.0	0.0	0.0	0.0
1.0	0.0	0.0	0.0	0.0	0.0	0.0	0.0
1.0	0.0	0.0	0.0	0.0	0.0	0.0	0.0
1.0	0.0	0.0	0.0	0.0	0.0	1.0	0.0
1.0	1.0	0.0	0.0	0.0	0.0	1.0	0.0
1.0	0.0	0.0	0.0	0.0	0.0	0.0	0.0
1.0	0.0	0.0	1.0	0.0	0.0	0.0	0.0
1.0	0.0	0.0	1.0	0.0	0.0	0.0	0.0
0.0	0.0	0.0	0.0	0.0	0.0	0.0	0.0
1.0	0.0	0.0	0.0	0.0	0.0	0.0	0.0
1.0	1.0	0.0	0.0	0.0	0.0	0.0	0.0
1.0	0.0	0.0	0.0	0.0	0.0	0.0	0.0
1.0	1.0	1.0	0.0	0.0	0.0	0.0	0.0
1.0	0.0	0.0	0.0	0.0	0.0	0.0	0.0
0.0	0.0	0.0	0.0	0.0	0.0	0.0	0.0
0.0	0.0	0.0	0.0	0.0	0.0	0.0	0.0
1.0	1.0	0.0	0.0	0.0	0.0	1.0	0.0
1.0	0.0	0.0	1.0	0.0	0.0	0.0	0.0
1.0	0.0	0.0	1.0	0.0	0.0	1.0	0.0
1.0	1.0	0.0	1.0	0.0	1.0	0.0	0.0
1.0	0.0	0.0	0.0	0.0	0.0	0.0	0.0
1.0	0.0	0.0	1.0	1.0	0.0	0.0	0.0
0.0	0.0	0.0	0.0	0.0	0.0	0.0	0.0
0.0	1.0	0.0	0.0	0.0	0.0	0.0	0.0

KT	Cr	eGFR	AF	ASP	CLOP	WAR	NOAC
0.0	0.93	64	0.0	1	0.0	1.0	0.0
0.0	8.41	7	1.0	1	0.0	0.0	0.0
0.0	0.76		0.0	0	0.0	0.0	0.0
0.0	1.06	73	0.0	1	1.0	0.0	0.0
0.0	1.60	45	1.0	0	0.0	1.0	0.0
0.0	0.00	0	0.0	1	1.0	0.0	0.0
1.0	0.66		0.0	1	0.0	0.0	0.0
0.0	10.63	5	0.0	1	1.0	0.0	0.0
0.0	10.32	5	#NULL!	1	1.0	0.0	0.0
0.0	1.07	52	0.0	0	1.0	0.0	0.0
0.0	14.12	4	0.0	0	0.0	0.0	0.0
0.0	0.89	64	0.0	0	1.0	0.0	0.0
0.0	0.48		#NULL!	0	0.0	0.0	0.0
0.0	6.11	7	#NULL!	0	0.0	0.0	0.0
0.0	4.71	9	0.0	0	1.0	0.0	0.0
1.0	1.55	45	1.0	1	0.0	1.0	0.0
0.0	1.00	75	1.0	0	0.0	0.0	0.0
0.0	4.43	10	0.0	1	1.0	0.0	0.0
0.0	0.95	79	1.0	1	0.0	0.0	0.0
0.0	0.72		1.0	1	1.0	0.0	0.0
0.0	0.69	84	0.0	0	0.0	0.0	0.0
0.0	3.21	19	0.0	1	1.0	0.0	0.0
0.0	1.09	67	0.0	0	0.0	0.0	0.0
0.0	1.60	32	1.0	0	0.0	0.0	0.0
1.0	1.12	65	1.0	1	1.0	0.0	1.0
0.0	1.19	60	0.0	0	1.0	0.0	0.0
0.0	0.85	66	0.0	0	0.0	0.0	0.0
0.0	2.53	25	0.0	0	1.0	0.0	0.0
0.0	2.50	32	0.0	0	0.0	0.0	0.0
0.0	8.55	51	0.0	0	0.0	0.0	0.0
0.0	1.00	54	#NULL!	0	0.0	1.0	0.0
0.0	0.40		0.0	1	1.0	0.0	0.0
0.0	1.03	52	0.0	0	0.0	0.0	0.0
0.0	0.45		0.0	0	1.0	0.0	0.0
0.0	0.61		0.0	0	0.0	0.0	0.0
0.0	0.00	0	0.0	0	0.0	0.0	0.0
0.0	0.48		#NULL!	1	1.0	0.0	1.0
0.0	0.73	78	1.0	1	0.0	0.0	0.0
0.0	1.05	67	0.0	0	1.0	0.0	0.0
0.0	7.59	7	0.0	1	0.0	0.0	0.0
0.0	0.56		1.0	0	0.0	0.0	0.0
0.0	0.75	75	#NULL!	1	1.0	0.0	0.0

0.0	0.79		0.0	1	0.0	0.0	0.0
0.0	0.70	81	0.0	0	0.0	0.0	0.0
0.0	3.25	19	0.0	0	0.0	0.0	0.0
0.0	1.74	38	0.0	1	0.0	0.0	0.0
0.0	#NULL!		0.0	0	0.0	0.0	0.0
0.0	0.99	73	1.0	0	0.0	0.0	0.0
0.0	0.59		1.0	0	0.0	1.0	0.0
0.0	#NULL!		#NULL!	0	1.0	0.0	0.0
0.0	1.89	35	0.0	1	1.0	0.0	0.0
0.0	0.70	70	0.0	0	0.0	0.0	0.0
0.0	2.45	34	0.0	0	0.0	0.0	0.0
0.0	0.69	82	#NULL!	0	1.0	0.0	0.0
0.0	1.30	40	1.0	0	0.0	0.0	0.0
1.0	1.67	40	1.0	0	1.0	1.0	0.0
0.0	1.10	65	#NULL!	0	0.0	1.0	0.0
0.0	3.36	13	1.0	0	0.0	0.0	0.0
0.0	1.35	51	#NULL!	0	0.0	1.0	0.0
0.0	1.07	66	0.0	1	1.0	1.0	0.0
0.0	0.82	67	1.0	0	0.0	1.0	0.0
0.0	1.83	36	1.0	0	0.0	0.0	1.0
0.0	0.00	0	0.0	1	1.0	0.0	0.0
0.0	1.04	51	#NULL!	0	0.0	1.0	0.0
0.0	0.00	0	0.0	1	0.0	0.0	0.0
0.0	3.14	20	1.0	0	0.0	0.0	0.0
0.0	0.94	57	#NULL!	0	0.0	0.0	0.0
0.0	0.57		1.0	0	0.0	0.0	0.0
0.0	1.16	45	#NULL!	1	0.0	0.0	0.0
0.0	3.00	15	0.0	0	0.0	0.0	0.0
0.0	0.82	1	#NULL!	1	0.0	0.0	0.0
0.0	1.65	40	0.0	0	1.0	0.0	0.0
0.0	0.99	52	0.0	0	0.0	1.0	0.0
0.0	0.97	73	0.0	1	0.0	0.0	0.0
0.0	0.87	83	#NULL!	0	1.0	0.0	0.0
0.0	0.90	80	0.0	0	0.0	0.0	0.0
0.0	2.17	29	0.0	0	0.0	0.0	0.0
0.0	1.22	42	0.0	1	1.0	0.0	0.0
0.0	0.00	0	0.0	0	0.0	0.0	0.0
0.0	1.27	40	0.0	0	1.0	0.0	0.0
0.0	1.47	45	0.0	0	1.0	0.0	0.0
0.0	0.48		#NULL!	0	1.0	0.0	0.0
0.0	2.29	20	1.0	0	0.0	0.0	0.0
0.0	1.55	42	0.0	0	0.0	0.0	0.0
0.0	0.55		0.0	1	0.0	0.0	0.0

0.0	0.53		0.0	0	1.0	0.0	0.0
0.0	0.84	48	#NULL!	0	0.0	0.0	0.0
0.0	#NULL!		#NULL!	0	1.0	0.0	0.0
1.0	1.17	71	#NULL!	0	0.0	0.0	0.0
0.0	7.68	6	0.0	0	0.0	0.0	0.0
0.0	#NULL!		#NULL!	0	0.0	0.0	0.0
0.0	5.13	13	0.0	0	0.0	0.0	0.0
0.0	0.75	86	#NULL!	0	0.0	0.0	0.0
0.0	10.21	4	0.0	0	0.0	0.0	0.0
0.0	3.74	13	#NULL!	1	0.0	0.0	0.0
1.0	11.38	5	0.0	0	0.0	0.0	0.0
1.0	12.22	4	0.0	1	0.0	0.0	0.0
0.0	7.85	7	1.0	0	1.0	0.0	0.0
1.0	8.67	7	0.0	1	0.0	0.0	0.0
0.0	0.76	81	1.0	1	0.0	0.0	0.0
0.0	#NULL!		0.0	1	0.0	0.0	0.0
0.0	6.91	6	0.0	0	0.0	0.0	0.0
0.0	10.98	4	0.0	1	0.0	0.0	0.0
0.0	0.77		0.0	1	1.0	0.0	0.0
0.0	2.94	17	0.0	0	0.0	0.0	0.0
1.0	10.58	5	#NULL!	1	1.0	0.0	0.0
0.0	4.80	13	0.0	0	0.0	0.0	0.0
0.0	0.76		0.0	0	0.0	0.0	0.0
0.0	0.57		0.0	1	0.0	0.0	0.0
1.0	1.43	38	0.0	1	1.0	0.0	0.0
0.0	0.00	0	0.0	0	0.0	0.0	0.0
0.0	14.76	3	0.0	0	0.0	0.0	0.0
1.0	1.62	36	1.0	0	0.0	0.0	0.0
0.0	5.04	9	0.0	0	1.0	0.0	0.0
0.0	0.58		0.0	1	0.0	0.0	0.0
0.0	0.55		#NULL!	0	0.0	0.0	0.0
0.0	8.40	5	0.0	0	1.0	0.0	0.0
1.0	1.35	55	0.0	0	0.0	0.0	0.0
0.0	#NULL!		0.0	1	0.0	0.0	0.0
1.0	3.27	15	0.0	0	0.0	0.0	0.0
1.0	8.07	14	0.0	0	1.0	0.0	0.0
1.0	6.52	9	#NULL!	0	0.0	0.0	0.0
0.0	10.35	5	0.0	1	1.0	0.0	0.0
0.0	1.13	67	#NULL!	0	1.0	0.0	0.0
0.0	0.52		0.0	0	0.0	0.0	0.0
0.0	0.89	88	0.0	0	0.0	0.0	0.0
0.0	0.65		0.0	0	0.0	0.0	1.0
0.0	4.25	11	0.0	0	0.0	0.0	0.0

0.0	8.31	7	0.0	1	0.0	0.0	0.0
0.0	3.15	15	0.0	1	1.0	0.0	0.0
0.0	8.53	6	#NULL!	1	1.0	0.0	0.0
0.0	0.91	85	0.0	0	0.0	0.0	0.0
1.0	4.67	10	0.0	1	0.0	0.0	0.0
0.0	7.51	6	0.0	0	0.0	0.0	0.0
0.0	5.20	8	#NULL!	1	1.0	0.0	0.0
0.0	0.00	0	0.0	0	0.0	0.0	0.0
0.0	6.59	9	0.0	0	0.0	0.0	0.0
0.0	0.93	83	0.0	0	0.0	0.0	0.0
0.0	2.20	20	1.0	0	0.0	0.0	0.0
0.0	8.48	6	0.0	0	1.0	0.0	0.0
1.0	10.33	5	1.0	1	0.0	0.0	0.0
1.0	0.83	69	0.0	0	0.0	0.0	0.0
0.0	0.71	83	0.0	0	0.0	0.0	0.0
0.0	0.57		0.0	1	0.0	0.0	0.0
0.0	1.00	56	0.0	1	0.0	0.0	0.0
0.0	9.31	4	0.0	0	0.0	0.0	0.0
0.0	8.88	6	#NULL!	0	0.0	0.0	0.0
0.0	0.62		#NULL!	0	0.0	0.0	0.0
0.0	1.42	50	0.0	1	0.0	0.0	0.0
0.0	0.64		0.0	1	1.0	0.0	0.0
0.0	#NULL!		0.0	1	0.0	0.0	0.0
0.0	8.82	14	0.0	1	1.0	0.0	0.0
0.0	6.07	9	0.0	1	1.0	0.0	0.0
0.0	0.97	78	0.0	1	0.0	0.0	0.0
0.0	0.54		0.0	1	0.0	0.0	0.0
0.0	0.99	56	0.0	1	0.0	0.0	0.0
0.0	1.03	72	#NULL!	1	0.0	0.0	0.0
0.0	1.08	51	0.0	1	1.0	0.0	0.0
0.0	3.65	11	0.0	1	0.0	0.0	0.0
0.0	11.30	5	0.0	1	0.0	0.0	0.0
0.0	0.85		0.0	0	1.0	0.0	0.0
0.0	1.04	71	#NULL!	1	0.0	0.0	0.0
1.0	10.29	4	0.0	0	0.0	0.0	0.0
0.0	0.89	63	1.0	0	0.0	0.0	1.0
0.0	0.56		0.0	1	0.0	0.0	0.0
0.0	#NULL!		#NULL!	0	0.0	0.0	0.0
0.0	0.96	78	0.0	1	0.0	0.0	0.0
0.0	9.50	6	0.0	0	0.0	0.0	0.0
0.0	11.04	3	#NULL!	0	0.0	0.0	0.0
0.0	0.58		0.0	1	0.0	0.0	0.0
0.0	1.06	52	0.0	1	0.0	0.0	0.0

0.0	116.00	15	0.0	0	0.0	0.0	0.0
0.0	0.00	0	0.0	0	0.0	0.0	0.0
0.0	10.98	5	#NULL!	0	0.0	0.0	0.0
0.0	0.72	81	0.0	0	0.0	0.0	0.0
0.0	8.93	4	0.0	0	0.0	0.0	0.0
0.0	0.85	67	0.0	1	0.0	0.0	0.0
0.0	#NULL!		1.0	0	1.0	1.0	0.0
0.0	0.85		0.0	1	0.0	0.0	0.0
0.0	1.13	48	#NULL!	0	1.0	0.0	0.0
0.0	2.71	23	0.0	0	0.0	0.0	0.0
0.0	1.10	65	#NULL!	0	0.0	0.0	0.0
0.0	8.47	5	0.0	0	1.0	0.0	0.0
0.0	1.09	67	1.0	0	0.0	0.0	1.0
0.0	0.60		1.0	0	0.0	1.0	0.0
0.0	#NULL!		#NULL!	0	0.0	0.0	0.0
0.0	#NULL!		#NULL!	0	0.0	0.0	0.0
0.0	6.17	7	0.0	0	0.0	0.0	0.0
0.0	4.68	12	0.0	1	0.0	0.0	0.0
0.0	1.24	58	0.0	1	0.0	0.0	0.0
0.0	0.85	66	0.0	0	1.0	0.0	0.0
0.0	0.74		0.0	0	0.0	0.0	0.0
0.0	4.36	10	0.0	1	0.0	0.0	0.0
0.0	1.89	26	0.0	1	0.0	0.0	0.0
0.0	0.50		0.0	1	1.0	0.0	0.0
0.0	2.12	31	0.0	0	1.0	0.0	0.0
0.0	0.56		#NULL!	1	0.0	0.0	0.0
0.0	0.66	88	#NULL!	1	0.0	0.0	0.0
0.0	0.46		0.0	1	0.0	0.0	0.0
0.0	2.98	15	1.0	1	1.0	1.0	0.0
0.0	0.00	0	0.0	0	0.0	0.0	0.0
0.0	11.45	13	0.0	1	0.0	1.0	0.0
0.0	1.33	53	0.0	1	1.0	0.0	0.0
0.0	0.78		0.0	0	0.0	0.0	0.0
0.0	3.04	20	0.0	0	0.0	0.0	0.0
0.0	4.47	10	#NULL!	1	0.0	0.0	0.0
0.0	0.00	0	0.0	0	0.0	0.0	0.0
0.0	0.00	0	0.0	1	0.0	0.0	0.0
1.0	1.29	41	1.0	1	0.0	0.0	0.0
0.0	1.16	46	0.0	0	0.0	0.0	0.0
0.0	0.50		0.0	0	0.0	0.0	0.0
0.0	0.42		1.0	1	0.0	0.0	0.0
0.0	1.00	73	0.0	1	0.0	0.0	0.0
0.0	0.75		0.0	0	0.0	0.0	0.0

0.0	10.93	12	1.0	0	0.0	0.0	0.0
0.0	0.64		#NULL!	0	0.0	0.0	0.0
0.0	5.52	8	0.0	1	0.0	0.0	0.0
0.0	0.72	79	1.0	0	0.0	1.0	0.0
0.0	0.80	70	0.0	0	1.0	0.0	0.0
0.0	0.86	65	#NULL!	1	1.0	0.0	0.0
0.0	1.95	34	0.0	1	0.0	0.0	0.0
0.0	1.64	41	0.0	0	0.0	0.0	0.0
0.0	0.75	76	0.0	1	0.0	0.0	0.0
0.0	0.59		0.0	1	0.0	0.0	0.0
0.0	8.06	13	0.0	1	0.0	0.0	0.0
0.0	6.75	6	0.0	0	1.0	0.0	0.0
0.0	1.03	53	0.0	1	0.0	0.0	0.0
0.0	1.07	50	#NULL!	0	0.0	1.0	0.0
0.0	0.92	60	0.0	1	0.0	0.0	0.0
0.0	1.51	34	0.0	1	0.0	0.0	0.0
0.0	1.03	71	1.0	1	0.0	0.0	0.0
0.0	0.69		0.0	0	0.0	0.0	0.0
0.0	0.92	60	0.0	1	0.0	0.0	0.0
0.0	0.61		0.0	1	0.0	0.0	0.0
0.0	0.95	58	1.0	0	0.0	0.0	0.0
0.0	4.46	13	0.0	1	1.0	0.0	0.0
0.0	0.66		0.0	0	0.0	0.0	0.0
0.0	0.74	77	0.0	1	0.0	0.0	0.0
0.0	0.58		0.0	0	0.0	0.0	0.0
0.0	3.91	15	0.0	1	1.0	0.0	0.0
0.0	1.02	71	0.0	1	0.0	0.0	0.0
0.0	#NULL!		1.0	0	0.0	0.0	0.0
0.0	0.89	62	1.0	1	0.0	0.0	0.0
0.0	1.16	62	0.0	0	0.0	0.0	0.0
0.0	0.68	85	1.0	0	0.0	0.0	1.0
0.0	0.68	85	0.0	0	0.0	0.0	0.0
0.0	1.09	49	1.0	0	1.0	1.0	0.0
0.0	0.87	64	0.0	1	0.0	0.0	0.0
0.0	0.74	77	#NULL!	1	0.0	0.0	0.0
0.0	0.99	55	0.0	0	0.0	0.0	0.0
0.0	0.66	88	1.0	1	0.0	0.0	0.0
0.0	#NULL!		0.0	0	0.0	0.0	0.0
0.0	1.00	54	0.0	0	0.0	0.0	1.0
0.0	1.13	47	0.0	0	0.0	0.0	0.0
0.0	0.84	66	0.0	1	0.0	0.0	0.0
0.0	0.49		0.0	1	0.0	0.0	0.0
0.0	2.49	19	0.0	0	0.0	0.0	0.0

0.0	0.55	0.0	0	0.0	0.0	0.0
0.0	1.11 48	0.0	1	1.0	0.0	0.0
0.0	0.77 73	#NULL!	0	0.0	0.0	0.0
0.0	0.43	0.0	0	0.0	0.0	0.0
0.0	0.77 73	0.0	0	0.0	0.0	0.0
0.0	0.58	1.0	0	1.0	0.0	0.0
0.0	0.75 75	0.0	0	0.0	0.0	1.0
0.0	0.74 77	0.0	0	0.0	0.0	0.0
0.0	3.23 19	0.0	1	0.0	0.0	0.0
0.0	0.70 82	#NULL!	0	0.0	0.0	0.0
0.0	0.88 63	#NULL!	0	0.0	0.0	0.0
0.0	0.65 89	0.0	1	0.0	0.0	0.0
0.0	1.44 35	0.0	0	1.0	0.0	0.0
0.0	1.10 48	0.0	1	0.0	0.0	0.0
0.0	1.00 54	1.0	0	1.0	0.0	1.0
0.0	0.76 74	0.0	0	0.0	0.0	0.0
0.0	2.69 17	0.0	0	0.0	0.0	0.0
0.0	0.80 70	1.0	0	0.0	0.0	1.0
0.0	0.65	0.0	0	0.0	0.0	0.0
0.0	0.69	0.0	0	0.0	0.0	0.0
1.0	1.46 47	0.0	0	1.0	0.0	0.0
0.0	8.01 5	#NULL!	0	0.0	0.0	0.0
0.0	0.73 78	0.0	0	0.0	0.0	0.0
0.0	0.94 1	0.0	0	0.0	0.0	0.0
0.0	0.36	1.0	1	0.0	0.0	0.0
0.0	1.12 64	1.0	0	0.0	0.0	0.0
0.0	0.96 76	0.0	0	0.0	0.0	0.0
0.0	3.06 20	#NULL!	1	0.0	1.0	0.0
0.0	0.67 86	0.0	1	1.0	0.0	0.0
0.0	1.42 48	#NULL!	1	0.0	0.0	0.0
0.0	0.72 79	0.0	1	0.0	0.0	0.0
0.0	0.88 62	0.0	1	0.0	0.0	0.0
0.0	0.64	0.0	0	0.0	0.0	0.0
0.0	0.76 74	0.0	0	0.0	0.0	0.0
0.0	0.98 74	0.0	1	1.0	0.0	0.0
0.0	1.14 53	0.0	0	0.0	0.0	0.0
0.0	6.18 7	1.0	1	0.0	0.0	0.0
0.0	1.47 46	0.0	0	0.0	0.0	0.0
0.0	0.70	0.0	1	0.0	0.0	0.0
0.0	0.81	0.0	0	0.0	0.0	0.0
0.0	2.57 18	0.0	1	1.0	0.0	0.0
0.0	0.59	0.0	0	0.0	0.0	0.0
0.0	1.28 42	1.0	0	0.0	0.0	0.0

0.0	1.64 47	0.0 0	0.0	0.0	0.0
0.0	8.01 5	0.0 0	0.0	0.0	0.0
0.0	0.57	#NULL! 1	0.0	0.0	0.0
0.0	0.64	0.0 1	0.0	0.0	0.0
0.0	7.51 5	0.0 1	1.0	0.0	0.0
0.0	0.58	1.0 0	0.0	0.0	1.0
0.0	4.75 12	0.0 0	0.0	0.0	0.0
0.0	0.68	0.0 1	0.0	0.0	0.0
0.0	1.26 55	1.0 1	0.0	1.0	0.0
1.0	0.70 81	#NULL! 0	0.0	0.0	0.0
0.0	1.03 70	#NULL! 0	0.0	1.0	0.0
0.0	0.76 61	0.0 0	1.0	0.0	0.0
0.0	1.14 46	0.0 1	0.0	0.0	0.0
0.0	0.52	0.0 1	1.0	0.0	0.0
0.0	1.49 34	0.0 1	0.0	0.0	0.0
0.0	0.79 70	0.0 1	0.0	0.0	0.0
0.0	0.88 62	0.0 0	0.0	0.0	0.0
0.0	1.05 38	#NULL! 0	0.0	0.0	0.0
0.0	1.23 42	0.0 1	0.0	0.0	0.0
0.0	0.79 70	0.0 1	0.0	0.0	0.0
0.0	0.88 74	0.0 1	0.0	0.0	0.0
0.0	0.84 88	1.0 0	0.0	0.0	0.0
0.0	#NULL!	#NULL! 1	0.0	0.0	0.0
0.0	0.51	0.0 0	0.0	0.0	0.0
0.0	0.67 85	1.0 0	0.0	1.0	0.0
0.0	0.88 62	1.0 0	0.0	1.0	0.0
0.0	0.96 56	#NULL! 1	0.0	0.0	0.0
0.0	0.64	0.0 1	0.0	0.0	0.0
0.0	0.70	0.0 1	0.0	0.0	0.0
0.0	2.37 27	1.0 0	0.0	0.0	0.0
0.0	0.89 82	0.0 0	0.0	0.0	0.0
0.0	0.96 56	0.0 0	0.0	0.0	0.0
0.0	8.05 10	0.0 1	1.0	0.0	0.0
0.0	1.02 70	0.0 0	0.0	0.0	0.0
0.0	0.70 81	0.0 0	0.0	0.0	0.0
0.0	1.36 51	0.0 0	1.0	0.0	1.0
0.0	1.48 46	0.0 0	0.0	0.0	0.0
0.0	0.74 76	0.0 1	0.0	0.0	0.0
0.0	1.14 62	1.0 1	0.0	0.0	0.0
0.0	0.98 55	0.0 0	0.0	0.0	0.0
0.0	0.77 72	#NULL! 1	0.0	0.0	0.0
0.0	0.54	1.0 0	0.0	0.0	1.0
0.0	0.65 88	0.0 1	0.0	0.0	0.0

0.0	0.94	77	#NULL!	0	0.0	0.0	0.0
0.0	1.65	30	0.0	1	0.0	0.0	0.0
0.0	4.06	11	0.0	0	1.0	0.0	0.0
0.0	1.31	39	0.0	0	0.0	0.0	0.0
0.0	0.62		0.0	1	0.0	0.0	0.0
0.0	#NULL!		0.0	1	0.0	0.0	0.0
0.0	0.78	71	0.0	0	0.0	0.0	0.0
0.0	5.36	8	0.0	0	0.0	0.0	0.0
0.0	1.56	32	0.0	0	0.0	0.0	0.0
0.0	2.55	24	1.0	1	1.0	0.0	0.0
0.0	2.46	25	0.0	0	0.0	0.0	0.0
0.0	0.00	0	0.0	0	0.0	0.0	0.0
0.0	1.12	80	1.0	1	0.0	0.0	1.0
0.0	1.62	30	#NULL!	0	1.0	0.0	0.0
0.0	0.68	83	#NULL!	1	0.0	0.0	0.0
0.0	1.00	53	0.0	1	0.0	0.0	0.0
0.0	0.96	56	#NULL!	1	0.0	0.0	0.0
0.0	1.05	50	1.0	0	0.0	1.0	0.0
0.0	1.07	48	0.0	0	0.0	0.0	0.0
0.0	1.18	44	0.0	0	0.0	0.0	0.0
0.0	0.77	72	0.0	0	1.0	0.0	0.0
0.0	1.06	54	1.0	1	0.0	0.0	0.0
0.0	0.70	80	1.0	1	0.0	0.0	0.0
0.0	0.72	78	0.0	0	0.0	0.0	0.0
0.0	1.31	53	0.0	0	0.0	0.0	0.0
0.0	1.82	36	#NULL!	1	0.0	0.0	0.0
0.0	1.21	43	0.0	0	0.0	0.0	0.0
0.0	1.74	28	#NULL!	1	0.0	0.0	0.0
0.0	1.55	32	0.0	1	0.0	1.0	0.0
0.0	0.69	81	0.0	0	0.0	1.0	0.0
0.0	0.75	74	0.0	1	0.0	0.0	0.0
0.0	0.83	66	0.0	1	0.0	0.0	0.0
0.0	1.76	28	#NULL!	0	0.0	0.0	0.0
0.0	1.12	47	#NULL!	0	0.0	0.0	0.0
0.0	2.20	21	#NULL!	0	0.0	0.0	0.0
0.0	3.71	12	0.0	1	0.0	0.0	0.0
0.0	1.45	47	#NULL!	0	1.0	0.0	0.0
0.0	0.44	90	0.0	1	0.0	0.0	0.0
0.0	0.86	63	0.0	0	0.0	1.0	0.0
0.0	20.90	78	0.0	0	0.0	0.0	0.0
0.0	1.23	42	0.0	1	0.0	0.0	0.0
0.0	7.53	7	1.0	0	0.0	1.0	0.0
0.0	0.52		0.0	0	0.0	1.0	0.0

0.0	1.11 47	0.0 0	0.0	0.0	0.0
0.0	0.64 89	1.0 0	0.0	1.0	0.0
0.0	1.15 45	0.0 1	0.0	0.0	0.0
0.0	0.96 75	1.0 0	0.0	1.0	0.0
0.0	0.56	1.0 0	1.0	0.0	0.0
0.0	0.94 57	0.0 0	0.0	0.0	0.0
0.0	1.85 26	0.0 1	0.0	0.0	0.0
0.0	1.45 37	0.0 0	1.0	0.0	0.0
0.0	0.76 73	0.0 0	0.0	0.0	0.0
0.0	0.00 0	0.0 1	0.0	0.0	0.0
0.0	0.00 0	0.0 0	0.0	0.0	0.0
0.0	#NULL!	0.0 0	1.0	0.0	0.0
0.0	1.99 36	#NULL! 1	0.0	0.0	0.0
0.0	0.91 80	0.0 0	0.0	0.0	0.0
0.0	0.89 82	0.0 1	0.0	0.0	0.0
0.0	0.92 58	#NULL! 0	0.0	0.0	0.0
0.0	8.43 5	1.0 1	0.0	0.0	0.0
0.0	1.13 62	1.0 1	0.0	0.0	0.0
0.0	0.97 74	0.0 0	0.0	0.0	0.0
0.0	0.47	0.0 0	0.0	0.0	0.0
0.0	0.00 0	0.0 1	0.0	0.0	0.0
0.0	2.72 22	1.0 0	1.0	0.0	0.0
0.0	0.89 60	0.0 0	0.0	0.0	0.0
0.0	0.93 77	0.0 0	0.0	0.0	0.0
0.0	1.22 57	0.0 1	0.0	0.0	0.0
0.0	0.94 57	0.0 0	1.0	0.0	0.0
0.0	0.95 76	0.0 0	0.0	0.0	0.0
0.0	0.44	0.0 0	0.0	0.0	0.0
0.0	1.67 29	0.0 1	0.0	0.0	0.0
0.0	0.93 57	#NULL! 1	0.0	0.0	0.0
0.0	1.00 48	1.0 1	1.0	0.0	0.0
0.0	0.69 81	#NULL! 0	0.0	0.0	0.0
0.0	0.61	1.0 1	0.0	0.0	1.0
0.0	0.58 90	1.0 1	0.0	0.0	0.0
0.0	1.01 52	0.0 0	0.0	0.0	0.0
0.0	0.94 57	0.0 0	0.0	0.0	0.0
0.0	1.40 36	#NULL! 0	1.0	0.0	0.0
0.0	0.87 83	0.0 1	0.0	0.0	0.0
0.0	0.64 88	#NULL! 1	0.0	0.0	0.0
0.0	3.02 15	0.0 0	0.0	0.0	0.0
0.0	0.70	0.0 0	0.0	0.0	0.0
0.0	0.58	0.0 0	0.0	0.0	0.0
0.0	0.53	0.0 1	0.0	0.0	0.0

0.0	0.93	57	1.0	0	0.0	0.0	1.0
0.0	1.19	43	1.0	0	0.0	0.0	1.0
0.0	1.00	53	0.0	0	0.0	0.0	0.0
0.0	1.07	48	0.0	1	1.0	0.0	0.0
0.0	0.71		0.0	0	0.0	0.0	0.0
0.0	1.09	47	0.0	0	0.0	0.0	0.0
0.0	0.81	67	1.0	1	1.0	0.0	0.0
0.0	1.11	46	0.0	0	0.0	0.0	0.0
0.0	0.75	73	1.0	0	0.0	0.0	0.0
0.0	0.87	62	1.0	1	0.0	0.0	0.0
0.0	1.69	38	0.0	0	0.0	0.0	0.0
0.0	1.00	52	#NULL!	0	0.0	0.0	0.0
0.0	0.66	85	0.0	0	0.0	0.0	0.0
0.0	0.77		1.0	1	0.0	0.0	0.0
0.0	0.58		#NULL!	1	1.0	0.0	0.0
0.0	0.84	64	0.0	0	0.0	1.0	0.0
0.0	0.81	66	0.0	0	0.0	0.0	0.0
0.0	1.34	65	0.0	1	0.0	0.0	0.0
0.0	0.71	77	#NULL!	0	0.0	0.0	0.0
0.0	0.52		#NULL!	1	0.0	0.0	0.0
0.0	0.93	57	#NULL!	0	0.0	0.0	0.0
0.0	0.69	80	0.0	0	0.0	0.0	0.0
0.0	0.49		0.0	0	0.0	0.0	0.0
0.0	1.11	62	#NULL!	1	0.0	0.0	0.0
0.0	1.18	58	0.0	1	0.0	0.0	0.0
0.0	1.59	29	0.0	1	0.0	0.0	0.0
0.0	0.69		0.0	1	0.0	0.0	0.0
0.0	0.63	88	1.0	1	0.0	0.0	0.0
0.0	1.01	41	0.0	0	0.0	0.0	1.0
0.0	0.58		0.0	0	0.0	0.0	0.0
0.0	0.00	0	0.0	0	0.0	0.0	0.0

RAS	BB	CCB	Diur	STATIN
1.0	0.0	1.0	1.0	1.0
1.0	1.0	0.0	0.0	1.0
0.0	0.0	0.0	0.0	1.0
0.0	0.0	1.0	0.0	1.0
1.0	1.0	0.0	1.0	1.0
1.0	1.0	1.0	0.0	1.0
0.0	1.0	0.0	1.0	1.0
1.0	1.0	1.0	0.0	1.0
0.0	0.0	0.0	0.0	1.0
0.0	1.0	0.0	0.0	1.0
1.0	0.0	1.0	0.0	1.0
1.0	1.0	1.0	0.0	1.0
0.0	0.0	1.0	1.0	0.0
1.0	0.0	1.0	0.0	1.0
1.0	1.0	0.0	0.0	0.0
0.0	0.0	0.0	1.0	1.0
0.0	0.0	0.0	0.0	0.0
1.0	1.0	1.0	1.0	1.0
1.0	0.0	0.0	1.0	0.0
0.0	0.0	1.0	0.0	1.0
1.0	0.0	0.0	0.0	1.0
0.0	0.0	1.0	0.0	1.0
0.0	1.0	0.0	0.0	1.0
0.0	1.0	1.0	1.0	0.0
1.0	0.0	1.0	0.0	0.0
1.0	1.0	0.0	0.0	1.0
1.0	0.0	1.0	0.0	0.0
1.0	1.0	1.0	0.0	1.0
0.0	0.0	0.0	0.0	0.0
0.0	0.0	0.0	0.0	0.0
0.0	0.0	1.0	1.0	0.0
0.0	1.0	0.0	0.0	1.0
0.0	1.0	1.0	0.0	1.0
1.0	1.0	1.0	1.0	1.0
1.0	1.0	0.0	0.0	0.0
0.0	0.0	0.0	0.0	0.0
0.0	0.0	0.0	0.0	1.0
1.0	1.0	0.0	0.0	0.0
1.0	0.0	1.0	1.0	0.0
1.0	0.0	1.0	1.0	1.0
0.0	0.0	0.0	0.0	0.0
1.0	0.0	1.0	0.0	1.0

1.0	0.0	1.0	0.0	1.0
0.0	0.0	0.0	0.0	0.0
0.0	0.0	0.0	0.0	0.0
0.0	0.0	0.0	0.0	1.0
1.0	1.0	0.0	1.0	1.0
0.0	0.0	0.0	1.0	0.0
0.0	0.0	0.0	1.0	0.0
0.0	0.0	1.0	0.0	1.0
1.0	1.0	1.0	0.0	1.0
1.0	0.0	1.0	0.0	1.0
1.0	1.0	0.0	1.0	1.0
0.0	0.0	1.0	0.0	1.0
0.0	0.0	0.0	0.0	0.0
0.0	0.0	1.0	0.0	1.0
0.0	1.0	1.0	0.0	1.0
0.0	1.0	1.0	0.0	1.0
0.0	1.0	0.0	1.0	0.0
1.0	0.0	0.0	0.0	1.0
1.0	1.0	0.0	0.0	1.0
1.0	0.0	1.0	0.0	0.0
0.0	0.0	1.0	0.0	1.0
0.0	0.0	0.0	1.0	1.0
1.0	0.0	0.0	1.0	0.0
0.0	0.0	1.0	1.0	0.0
1.0	1.0	1.0	1.0	1.0
0.0	0.0	0.0	1.0	1.0
1.0	1.0	0.0	1.0	0.0
1.0	1.0	1.0	1.0	0.0
1.0	1.0	0.0	0.0	0.0
1.0	0.0	1.0	0.0	1.0
1.0	0.0	0.0	1.0	1.0
0.0	0.0	0.0	0.0	0.0
0.0	1.0	1.0	1.0	0.0
1.0	0.0	0.0	0.0	0.0
0.0	1.0	0.0	0.0	0.0
0.0	0.0	1.0	0.0	1.0
0.0	0.0	0.0	0.0	0.0
0.0	0.0	0.0	0.0	1.0
1.0	0.0	0.0	1.0	0.0
1.0	1.0	1.0	0.0	0.0
0.0	1.0	0.0	1.0	1.0
1.0	0.0	0.0	0.0	1.0
1.0	0.0	1.0	0.0	0.0

1.0	1.0	1.0	1.0	1.0
1.0	0.0	1.0	1.0	0.0
1.0	0.0	0.0	0.0	1.0
1.0	1.0	1.0	0.0	0.0
1.0	1.0	1.0	1.0	0.0
0.0	0.0	0.0	1.0	0.0
1.0	1.0	0.0	1.0	0.0
1.0	0.0	0.0	1.0	1.0
1.0	1.0	1.0	1.0	0.0
1.0	1.0	0.0	0.0	1.0
0.0	0.0	0.0	0.0	0.0
0.0	1.0	1.0	0.0	0.0
0.0	0.0	0.0	0.0	0.0
0.0	0.0	1.0	0.0	1.0
0.0	1.0	0.0	1.0	0.0
1.0	0.0	1.0	1.0	0.0
1.0	1.0	1.0	0.0	0.0
0.0	1.0	0.0	0.0	1.0
0.0	0.0	0.0	0.0	0.0
0.0	1.0	1.0	0.0	0.0
0.0	0.0	0.0	0.0	1.0
0.0	0.0	0.0	0.0	0.0
0.0	0.0	0.0	0.0	0.0
0.0	1.0	0.0	0.0	1.0
0.0	1.0	0.0	0.0	1.0
0.0	0.0	0.0	1.0	1.0
0.0	0.0	0.0	0.0	0.0
1.0	1.0	0.0	0.0	1.0
0.0	1.0	1.0	0.0	1.0
1.0	0.0	1.0	0.0	1.0
0.0	0.0	0.0	0.0	0.0
0.0	0.0	1.0	0.0	1.0
0.0	0.0	0.0	0.0	0.0
1.0	1.0	1.0	0.0	1.0
0.0	0.0	0.0	0.0	1.0
1.0	1.0	1.0	0.0	1.0
1.0	1.0	1.0	1.0	0.0
1.0	0.0	1.0	0.0	1.0
1.0	1.0	0.0	1.0	1.0
1.0	0.0	1.0	0.0	1.0
0.0	0.0	1.0	0.0	1.0
0.0	1.0	0.0	0.0	1.0
0.0	0.0	0.0	0.0	0.0

1.0	1.0	1.0	0.0	1.0
1.0	1.0	0.0	0.0	1.0
0.0	1.0	1.0	0.0	1.0
1.0	1.0	0.0	0.0	1.0
0.0	0.0	1.0	0.0	0.0
0.0	0.0	0.0	0.0	1.0
1.0	1.0	1.0	0.0	1.0
0.0	0.0	0.0	0.0	0.0
0.0	0.0	0.0	0.0	0.0
0.0	0.0	0.0	0.0	0.0
0.0	0.0	0.0	0.0	0.0
1.0	0.0	0.0	0.0	0.0
0.0	0.0	0.0	0.0	0.0
1.0	0.0	0.0	1.0	1.0
0.0	0.0	0.0	0.0	1.0
1.0	1.0	1.0	0.0	1.0
1.0	0.0	0.0	1.0	0.0
1.0	1.0	0.0	1.0	1.0
1.0	1.0	1.0	0.0	0.0
1.0	0.0	0.0	1.0	1.0
1.0	0.0	1.0	0.0	1.0
1.0	0.0	1.0	0.0	1.0
0.0	0.0	0.0	0.0	1.0
0.0	0.0	0.0	0.0	1.0
0.0	1.0	0.0	1.0	0.0
0.0	0.0	1.0	0.0	1.0
1.0	1.0	0.0	0.0	0.0
0.0	1.0	0.0	0.0	1.0
1.0	1.0	1.0	1.0	1.0
0.0	0.0	0.0	0.0	1.0
1.0	0.0	1.0	0.0	0.0
1.0	1.0	1.0	1.0	1.0
1.0	1.0	0.0	0.0	1.0
1.0	0.0	0.0	1.0	1.0
0.0	0.0	0.0	0.0	0.0
1.0	0.0	0.0	1.0	0.0
1.0	0.0	0.0	1.0	0.0
0.0	0.0	0.0	0.0	0.0
1.0	0.0	1.0	0.0	1.0
1.0	1.0	1.0	1.0	0.0
1.0	1.0	1.0	1.0	1.0
0.0	0.0	0.0	0.0	0.0
1.0	0.0	1.0	0.0	1.0

0.0	0.0	0.0	0.0	0.0
0.0	0.0	0.0	0.0	0.0
1.0	0.0	0.0	1.0	0.0
0.0	0.0	1.0	0.0	1.0
1.0	0.0	1.0	0.0	0.0
1.0	0.0	0.0	1.0	0.0
1.0	1.0	1.0	1.0	0.0
1.0	0.0	0.0	0.0	1.0
1.0	1.0	0.0	1.0	1.0
0.0	0.0	1.0	0.0	0.0
0.0	0.0	0.0	0.0	0.0
0.0	0.0	0.0	0.0	0.0
0.0	0.0	1.0	0.0	1.0
1.0	0.0	1.0	0.0	0.0
1.0	1.0	1.0	1.0	1.0
0.0	0.0	0.0	0.0	0.0
1.0	1.0	0.0	1.0	0.0
0.0	0.0	0.0	0.0	0.0
1.0	1.0	0.0	0.0	1.0
0.0	0.0	1.0	0.0	1.0
1.0	0.0	1.0	0.0	0.0
1.0	0.0	1.0	0.0	1.0
0.0	0.0	1.0	1.0	0.0
1.0	0.0	0.0	0.0	1.0
1.0	1.0	0.0	1.0	1.0
1.0	1.0	1.0	0.0	0.0
0.0	0.0	1.0	0.0	1.0
0.0	0.0	0.0	0.0	1.0
0.0	0.0	0.0	1.0	1.0
1.0	0.0	1.0	1.0	0.0
0.0	0.0	0.0	0.0	1.0
1.0	0.0	1.0	1.0	1.0
0.0	0.0	0.0	0.0	0.0
0.0	0.0	0.0	0.0	0.0
0.0	0.0	1.0	0.0	0.0
1.0	1.0	0.0	1.0	1.0
0.0	0.0	0.0	0.0	0.0
1.0	0.0	0.0	0.0	0.0
0.0	0.0	0.0	0.0	0.0
0.0	1.0	0.0	0.0	1.0
0.0	1.0	1.0	0.0	0.0
1.0	1.0	0.0	1.0	0.0
1.0	0.0	0.0	0.0	1.0

0.0	0.0	0.0	0.0	0.0
0.0	0.0	0.0	0.0	0.0
0.0	0.0	1.0	0.0	1.0
0.0	0.0	1.0	1.0	1.0
0.0	0.0	1.0	0.0	1.0
1.0	0.0	1.0	1.0	0.0
1.0	0.0	0.0	0.0	0.0
0.0	0.0	0.0	0.0	0.0
0.0	1.0	1.0	0.0	1.0
1.0	1.0	1.0	0.0	1.0
0.0	0.0	0.0	0.0	1.0
1.0	1.0	1.0	0.0	0.0
1.0	0.0	1.0	0.0	1.0
1.0	0.0	0.0	1.0	1.0
0.0	0.0	1.0	0.0	1.0
1.0	1.0	0.0	1.0	1.0
1.0	0.0	0.0	1.0	0.0
0.0	0.0	0.0	0.0	0.0
0.0	0.0	1.0	0.0	1.0
0.0	0.0	0.0	0.0	1.0
0.0	0.0	0.0	1.0	0.0
0.0	1.0	0.0	1.0	1.0
1.0	1.0	1.0	0.0	1.0
1.0	0.0	1.0	0.0	1.0
0.0	1.0	1.0	0.0	0.0
1.0	1.0	0.0	0.0	1.0
1.0	1.0	1.0	1.0	1.0
0.0	0.0	1.0	1.0	1.0
0.0	1.0	1.0	1.0	0.0
1.0	0.0	0.0	0.0	0.0
0.0	0.0	0.0	1.0	1.0
1.0	0.0	0.0	0.0	0.0
1.0	0.0	1.0	1.0	1.0
1.0	1.0	0.0	0.0	1.0
0.0	0.0	0.0	0.0	1.0
0.0	1.0	1.0	1.0	0.0
0.0	0.0	1.0	1.0	0.0
0.0	0.0	0.0	0.0	0.0
0.0	1.0	0.0	1.0	0.0
1.0	0.0	0.0	0.0	0.0
1.0	1.0	0.0	0.0	1.0
1.0	0.0	1.0	1.0	1.0
1.0	1.0	1.0	1.0	1.0

1.0	0.0	0.0	1.0	0.0
1.0	0.0	0.0	0.0	1.0
0.0	0.0	1.0	0.0	0.0
0.0	0.0	1.0	0.0	0.0
1.0	1.0	0.0	1.0	0.0
0.0	0.0	1.0	0.0	0.0
1.0	0.0	1.0	1.0	1.0
1.0	0.0	1.0	1.0	0.0
1.0	0.0	1.0	1.0	1.0
1.0	0.0	1.0	0.0	0.0
1.0	0.0	0.0	0.0	1.0
1.0	0.0	1.0	0.0	0.0
0.0	0.0	1.0	1.0	1.0
1.0	0.0	1.0	1.0	0.0
1.0	0.0	1.0	1.0	1.0
0.0	0.0	1.0	0.0	0.0
1.0	0.0	1.0	0.0	0.0
0.0	1.0	0.0	1.0	0.0
0.0	0.0	0.0	0.0	0.0
0.0	0.0	0.0	0.0	0.0
0.0	0.0	1.0	1.0	1.0
0.0	0.0	1.0	0.0	0.0
1.0	1.0	1.0	0.0	1.0
1.0	0.0	1.0	1.0	1.0
0.0	1.0	0.0	0.0	1.0
0.0	1.0	0.0	0.0	1.0
0.0	0.0	1.0	0.0	0.0
0.0	0.0	1.0	1.0	1.0
1.0	1.0	1.0	1.0	1.0
0.0	1.0	0.0	0.0	1.0
1.0	0.0	0.0	0.0	1.0
1.0	0.0	0.0	0.0	1.0
1.0	0.0	1.0	0.0	1.0
0.0	1.0	0.0	1.0	0.0
1.0	0.0	0.0	1.0	1.0
0.0	0.0	0.0	0.0	0.0
0.0	0.0	0.0	0.0	1.0
0.0	0.0	0.0	0.0	0.0
1.0	0.0	1.0	0.0	0.0
0.0	0.0	0.0	0.0	0.0
0.0	0.0	0.0	1.0	1.0
0.0	0.0	0.0	0.0	0.0
0.0	0.0	0.0	0.0	0.0

0.0	0.0	0.0	0.0	0.0
0.0	0.0	0.0	1.0	1.0
0.0	0.0	1.0	0.0	1.0
0.0	0.0	0.0	0.0	1.0
0.0	0.0	1.0	0.0	1.0
0.0	1.0	0.0	1.0	0.0
1.0	1.0	1.0	1.0	1.0
0.0	0.0	0.0	0.0	1.0
1.0	1.0	0.0	1.0	0.0
1.0	1.0	1.0	1.0	1.0
1.0	1.0	1.0	0.0	0.0
0.0	1.0	1.0	1.0	1.0
1.0	1.0	0.0	1.0	0.0
0.0	0.0	0.0	0.0	0.0
0.0	0.0	0.0	0.0	0.0
0.0	1.0	1.0	0.0	0.0
1.0	1.0	1.0	1.0	0.0
0.0	0.0	0.0	0.0	0.0
1.0	0.0	1.0	1.0	0.0
0.0	0.0	0.0	0.0	0.0
1.0	0.0	1.0	0.0	1.0
1.0	1.0	0.0	1.0	0.0
0.0	0.0	0.0	0.0	1.0
0.0	0.0	0.0	0.0	0.0
1.0	1.0	1.0	1.0	0.0
1.0	0.0	0.0	1.0	0.0
1.0	0.0	0.0	0.0	1.0
0.0	0.0	1.0	0.0	1.0
1.0	0.0	0.0	1.0	1.0
0.0	0.0	1.0	0.0	0.0
1.0	0.0	0.0	0.0	0.0
0.0	0.0	0.0	0.0	0.0
0.0	1.0	0.0	1.0	0.0
0.0	0.0	0.0	0.0	0.0
0.0	0.0	0.0	0.0	0.0
0.0	1.0	0.0	1.0	1.0
0.0	0.0	0.0	0.0	0.0
1.0	0.0	1.0	0.0	1.0
1.0	0.0	0.0	0.0	0.0
1.0	0.0	1.0	0.0	0.0
1.0	0.0	0.0	0.0	1.0
1.0	1.0	0.0	1.0	1.0
1.0	0.0	1.0	1.0	1.0

0.0	0.0	0.0	1.0	0.0
0.0	1.0	1.0	1.0	1.0
0.0	1.0	1.0	0.0	0.0
1.0	0.0	1.0	1.0	0.0
0.0	0.0	1.0	1.0	1.0
1.0	1.0	0.0	0.0	1.0
0.0	0.0	0.0	0.0	0.0
0.0	0.0	0.0	0.0	0.0
1.0	0.0	0.0	0.0	1.0
0.0	0.0	0.0	1.0	0.0
0.0	0.0	0.0	0.0	0.0
0.0	0.0	0.0	0.0	0.0
0.0	1.0	1.0	0.0	0.0
1.0	1.0	0.0	0.0	1.0
1.0	1.0	1.0	0.0	1.0
0.0	1.0	1.0	1.0	0.0
1.0	0.0	1.0	0.0	1.0
1.0	0.0	1.0	0.0	1.0
1.0	1.0	1.0	0.0	0.0
1.0	0.0	0.0	0.0	0.0
1.0	1.0	1.0	1.0	1.0
0.0	1.0	0.0	0.0	0.0
0.0	1.0	0.0	0.0	0.0
1.0	0.0	0.0	1.0	0.0
0.0	0.0	0.0	0.0	0.0
1.0	0.0	0.0	1.0	0.0
1.0	0.0	1.0	0.0	1.0
0.0	0.0	0.0	0.0	1.0
0.0	0.0	1.0	1.0	1.0
0.0	0.0	0.0	0.0	1.0
1.0	1.0	0.0	1.0	1.0
1.0	1.0	1.0	0.0	1.0
1.0	0.0	1.0	1.0	0.0
1.0	1.0	1.0	0.0	0.0
0.0	0.0	0.0	0.0	0.0
1.0	1.0	1.0	1.0	1.0
0.0	0.0	0.0	0.0	0.0
1.0	0.0	1.0	0.0	0.0
1.0	0.0	0.0	1.0	1.0
0.0	0.0	0.0	0.0	0.0
1.0	0.0	0.0	1.0	1.0
0.0	0.0	0.0	0.0	0.0
0.0	0.0	0.0	0.0	1.0

1.0	0.0	0.0	1.0	0.0
1.0	1.0	1.0	1.0	1.0
0.0	1.0	1.0	0.0	1.0
1.0	0.0	0.0	1.0	0.0
0.0	1.0	0.0	0.0	0.0
0.0	0.0	0.0	0.0	0.0
0.0	0.0	1.0	1.0	0.0
1.0	1.0	1.0	1.0	0.0
0.0	0.0	0.0	0.0	0.0
0.0	0.0	1.0	0.0	1.0
0.0	0.0	0.0	0.0	0.0
1.0	1.0	1.0	0.0	1.0
1.0	0.0	1.0	1.0	1.0
1.0	0.0	1.0	0.0	0.0
1.0	0.0	1.0	0.0	1.0
0.0	0.0	1.0	1.0	0.0
1.0	0.0	1.0	1.0	1.0
0.0	0.0	0.0	1.0	1.0
0.0	0.0	1.0	0.0	0.0
0.0	0.0	0.0	0.0	1.0
1.0	1.0	0.0	1.0	0.0
1.0	0.0	0.0	0.0	1.0
0.0	0.0	0.0	0.0	0.0
0.0	0.0	0.0	0.0	0.0
1.0	0.0	1.0	0.0	0.0
1.0	1.0	1.0	0.0	1.0
0.0	0.0	0.0	0.0	0.0
1.0	0.0	1.0	1.0	0.0
1.0	1.0	1.0	1.0	1.0
0.0	0.0	0.0	1.0	1.0
1.0	0.0	0.0	1.0	0.0
0.0	0.0	1.0	0.0	0.0
1.0	0.0	1.0	1.0	1.0
1.0	1.0	0.0	1.0	0.0
0.0	0.0	0.0	0.0	0.0
0.0	1.0	0.0	0.0	0.0
0.0	0.0	0.0	0.0	0.0
0.0	0.0	1.0	0.0	1.0
1.0	1.0	1.0	0.0	1.0
1.0	1.0	1.0	1.0	0.0
1.0	0.0	1.0	0.0	0.0
0.0	0.0	1.0	0.0	0.0
1.0	0.0	0.0	1.0	0.0

0.0	1.0	0.0	1.0	0.0
1.0	0.0	1.0	0.0	0.0
1.0	0.0	1.0	1.0	0.0
0.0	1.0	0.0	0.0	1.0
0.0	0.0	0.0	0.0	0.0
0.0	0.0	0.0	1.0	0.0
0.0	1.0	0.0	1.0	1.0
1.0	0.0	0.0	1.0	0.0
1.0	0.0	0.0	1.0	0.0
1.0	0.0	0.0	1.0	0.0
0.0	0.0	1.0	1.0	0.0
0.0	1.0	0.0	0.0	0.0
1.0	1.0	0.0	0.0	0.0
1.0	0.0	0.0	1.0	1.0
1.0	1.0	0.0	0.0	1.0
0.0	0.0	0.0	0.0	0.0
1.0	0.0	0.0	1.0	0.0
1.0	1.0	0.0	1.0	0.0
0.0	0.0	1.0	0.0	1.0
0.0	0.0	1.0	0.0	1.0
1.0	0.0	0.0	1.0	0.0
1.0	1.0	0.0	1.0	0.0
0.0	0.0	0.0	0.0	0.0
1.0	0.0	0.0	0.0	1.0
1.0	1.0	1.0	1.0	0.0
1.0	1.0	0.0	1.0	1.0
0.0	0.0	0.0	1.0	0.0
1.0	0.0	1.0	0.0	0.0
0.0	0.0	0.0	1.0	1.0
0.0	0.0	0.0	0.0	0.0
0.0	0.0	0.0	0.0	0.0
